# Supplementary material for: Prognostic value of ki67 in BCG-treated non-muscle invasive bladder cancer: a meta-analysis and systematic review
Source: BMJ Open. 2018 Apr 17;8(4):e019635. doi: 10.1136/bmjopen-2017-019635 (PMC5905754; doi:10.1136/bmjopen-2017-019635)
Supplement: Supplementary data [file bmjopen-2017-019635supp006.pdf]

Prognostic Value of ki67 in Bacillus Calmette–Guérin-Treated Non-muscle-Invasive Bladder Cancer: a Meta-analysis and Systematic Review

Search strategy in PubMed.

The last quest was updated on May 24, 2017.

#1 Search “Urinary Bladder Neoplasms” [Mesh]

#2 Search bladder cancer

#3 Search bladder carcinoma

#4 Search bladder tumor

#5 Search (#1 or #2 or #3 or #4)

#6 Search "BCG Vaccine" [Mesh]

#7 Search BCG

#8 Search Bacillus Calmette–Guérin

#9 Search (#6 or #7 or #8)

#10 Search "ki-67 antigen" [Mesh]

#11 Search ki-67

#12 Search ki67

#13 Search MBI-1

#14 Search (#10 or #11 or #12 or #13)

#15 Search (#5 and #9 and #14)

The enrolled contents included the reference lists and relevant suggestive references while searching.
