# Supplementary material for: Prognostic value of ki67 in BCG-treated non-muscle invasive bladder cancer: a meta-analysis and systematic review
Source: BMJ Open. 2018 Apr 17;8(4):e019635. doi: 10.1136/bmjopen-2017-019635 (PMC5905754; doi:10.1136/bmjopen-2017-019635)
Supplement: Supplementary data [file bmjopen-2017-019635supp001.pdf]

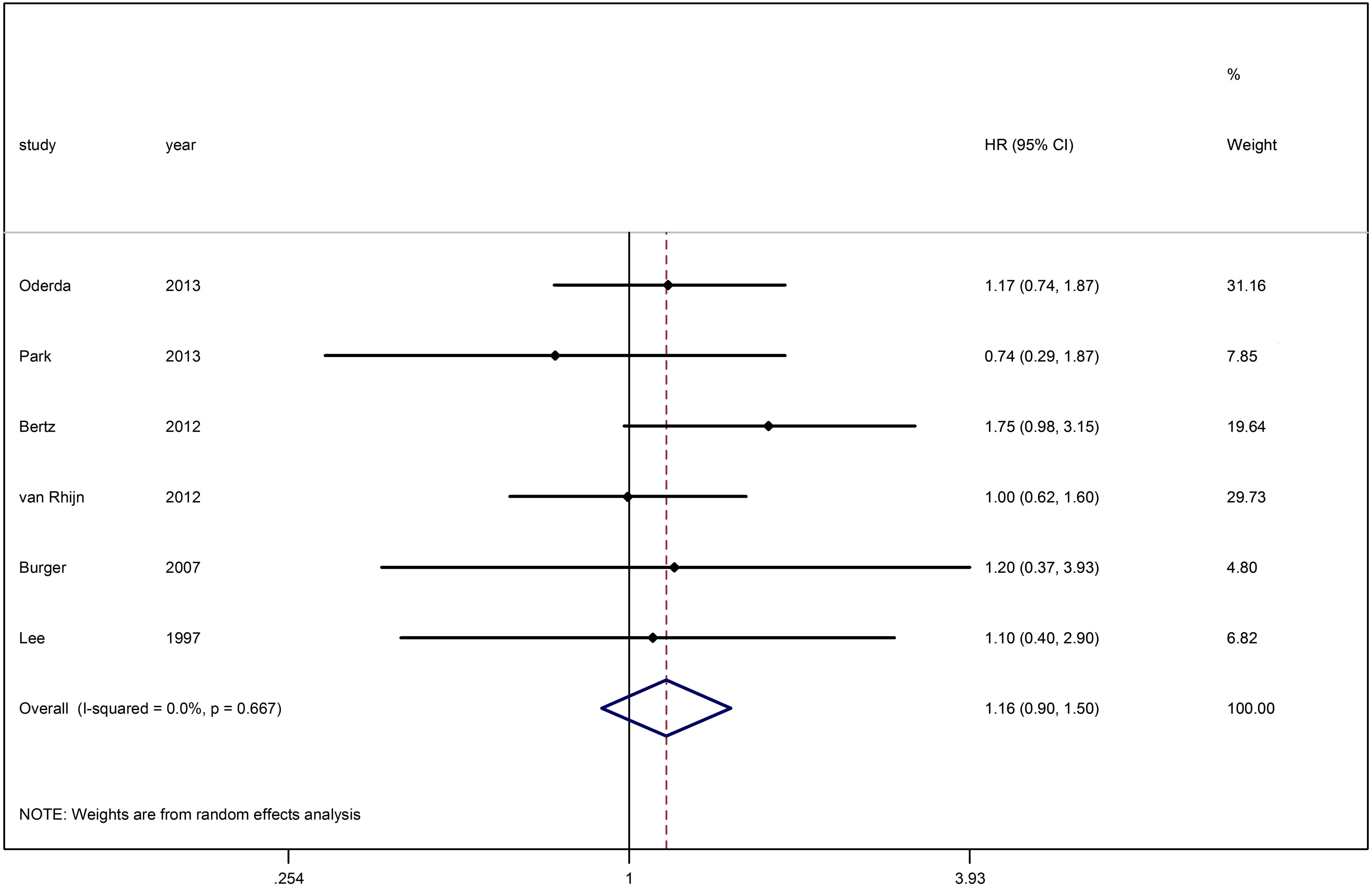

Figure S1. Forest plots of HRs estimated for the relationship between the expression of ki67 and RFS after the aforementioned study was excluded
