# Supplementary material for: Prognostic value of ki67 in BCG-treated non-muscle invasive bladder cancer: a meta-analysis and systematic review
Source: BMJ Open. 2018 Apr 17;8(4):e019635. doi: 10.1136/bmjopen-2017-019635 (PMC5905754; doi:10.1136/bmjopen-2017-019635)
Supplement: Supplementary data [file bmjopen-2017-019635supp004.pdf]

Table S2. Meta-regression analysis of RFS and PFS

PFS

Heterogeneity factor	Coefficient	SE	*t*	*P*
Years	–0.0343	0.0283	–1.21	0.280
Country				
1	–0.3899	0.3716	–1.05	0.404
2	–0.7185	0.3503	–2.05	0.177
3	–0.9900	0.4307	–2.30	0.148
4	–0.8805	0.3541	–2.49	0.131
Numbers of patients	0.0022	0.0020	1.16	0.299
Stage	None			
Cutoff	–0.0098	0.0407	–0.24	0.819
Age	0.0021	0.0395	0.05	0.959
Follow-up	–0.0062	0.0065	–0.96	0.379

PFS

Heterogeneity factor	Coefficient	SE	*t*	*P*
Years	–0.1195	0.0461	–2.59	0.036
Country				

1	0.2080	0.7936	0.26	0.818
2	–0.8062	0.5662	–1.42	0.290
3	–1.4505	0.8858	–1.64	0.243
4	–2.7009	0.9407	–2.87	0.103
5	–1.7766	0.6167	–2.88	0.102
6	–0.8158	0.5281	–1.54	0.262
Numbers of patients	0.0006	0.0036	0.16	0.877
Stage				
1	–1.4505	1.5909	–0.91	0.458
2	–0.8062	1.4108	–0.57	0.625
3	0.2080	1.5332	0.14	0.904
4	–1.7766	1.4353	–1.24	0.341
5	–1.2560	1.2069	–1.04	0.407
6	–0.6170	1.4689	–0.42	0.715
Cutoff	–0.0177	0.0309	–0.57	0.585
Age	–0.0672	0.0757	–0.89	0.404
Follow-up	–0.0118	0.0159	–0.74	0.483
