# Supplementary material for: Prognostic value of ki67 in BCG-treated non-muscle invasive bladder cancer: a meta-analysis and systematic review
Source: BMJ Open. 2018 Apr 17;8(4):e019635. doi: 10.1136/bmjopen-2017-019635 (PMC5905754; doi:10.1136/bmjopen-2017-019635)
Supplement: Supplementary data [file bmjopen-2017-019635supp005.pdf]

Table S3. Risk group stratification in NMIBC

**Low-risk tumors**	Primary, solitary, Ta, LG/G1, <3 cm, no CIS
**Intermediate-risk tumors**	All tumors not defined in the two adjacent categories (between the category of low and high risk)
**High-risk tumors**	Any of the following:<br>T1 tumor<br>HG/G3 tumor<br>CIS<br>Multiple, recurrent, and large (>3 cm) Ta G1G2 tumors (all conditions must be present at this point)

NMIBC, non-muscle-invasive bladder cancer; CIS, carcinoma in situ; HG, high grade; LG, low grade.
