# Supplementary material for: Prognostic value of ki67 in BCG-treated non-muscle invasive bladder cancer: a meta-analysis and systematic review
Source: BMJ Open. 2018 Apr 17;8(4):e019635. doi: 10.1136/bmjopen-2017-019635 (PMC5905754; doi:10.1136/bmjopen-2017-019635)
Supplement: Author's manuscript [file bmjopen-2017-019635.draft_revisions.pdf]

BMJ Open is committed to open peer review. As part of this commitment we make the peer review history of every article we publish publicly available.

When an article is published we post the peer reviewers' comments and the authors' responses online. We also post the versions of the paper that were used during peer review. These are the versions that the peer review comments apply to.

The versions of the paper that follow are the versions that were submitted during the peer review process. They are not the versions of record or the final published versions. They should not be cited or distributed as the published version of this manuscript.

BMJ Open is an open access journal and the full, final, typeset and author-corrected version of record of the manuscript is available on our site with no access controls, subscription charges or pay-per-view fees (http://bmjopen.bmj.com).

If you have any questions on BMJ Open's open peer review process please email
editorial.bmjopen@bmj.com

BMJ Open

Prognostic Value of ki67 in Bacillus Calmette–Guérin-Treated Non-muscle-Invasive Bladder Cancer: a Meta-analysis and Systematic Review

Journal:	*BMJ Open*
Manuscript ID	bmjopen-2017-019635
Article Type:	Research
Date Submitted by the Author:	20-Sep-2017
Complete List of Authors:	He, Yuhui; Peking University China-Japan Friendship School of Clinical Medicine; China-Japan Friendship Hospital, Department of Urology<br>Wang, Ning; North China University of Science and Technology<br>Zhou, Xiaofeng; Peking University China-Japan Friendship School of Clinical Medicine; China-Japan Friendship Hospital, Department of Urology<br>Wang, Jianfeng; China-Japan Friendship Hospital, Department of Urology<br>Ding, Zhenshan; China-Japan Friendship Hospital, Department of Urology<br>Chen, Xing ; China-Japan Friendship Hospital, Department of Urology<br>Deng, Yisen; Peking University China-Japan Friendship School of Clinical Medicine; China-Japan Friendship Hospital, Department of Urology
**Primary Subject Heading**:	Urology
Secondary Subject Heading:	Urology, Addiction
Keywords:	Ki67, meta-analysis, non-muscular-invasive bladder cancer;, prognosis

SCHOLARONE™
Manuscripts

Prognostic Value of ki67 in Bacillus Calmette–Guérin-Treated Non-muscle-Invasive Bladder Cancer: a Meta-analysis and Systematic Review

Yuhui He[1, 2], Ning Wang[3], Xiaofeng Zhou[1, 2*], Jianfeng Wang[2], Zhenshan Ding[2], Xing Chen[2], Yisen Deng[1, 2]

[1] Peking University China-Japan Friendship School of Clinical Medicine, Beijing 100029, China

[2] Department of Urology, China-Japan Friendship Hospital, Beijing 100029, China

[3] North China University of Science and Technology, Tangshan 063013, China

***Corresponding Author**

Xiaofeng Zhou

Peking University China-Japan Friendship School of Clinical Medicine, Beijing 100029, China

Department of Urology, China-Japan Friendship Hospital, Beijing 100029, China

Tel: +86 1590110219

Email： doctorzxf@126.com

Fax: +86 10 6421 7749

Word count

Title page: 105

Abstract: 256

References: 1211

For peer review only - http://bmjopen.bmj.com/site/about/guidelines.xhtml

Figures and tables: 7, 4

For peer review only

For peer review only - http://bmjopen.bmj.com/site/about/guidelines.xhtml

Abstract

Objectives: The aim of this study was to explore the prognostic value of ki67 as a marker in Bacillus Calmette–Guérin (BCG)-treated non-muscle-invasive bladder cancer (NMIBC).

Methods: Studies were systematically retrieved from the relevant databases (Web of Science, PubMed, Cochrane Library, and Embase), and the expiry date was May 2017. The research steps referred to the Preferred Reporting Items for Systematic Reviews and Meta-Analysis Statement.

Results: A total of 11 studies, including 1321 cases, complying with the inclusion criteria were enrolled. The results of the meta-analysis indicated that the expression of ki67 was not statistically significantly correlated with recurrence-free survival (RFS). No significant heterogeneity was found among all included studies. The expression of ki67 was statistically significantly correlated with progression-free survival (PFS), and the overexpression of ki67 was the risk factor for PFS. Statistical heterogeneity was noted among all the included studies. The studies that might cause heterogeneity were excluded using the Galbraith plot, and then the meta-analysis was performed again. The results showed that the expression of ki67 was still correlated with PFS. In the Caucasian subgroup, the overexpression of ki67 was the risk factor for RFS and PFS.

Conclusions: For the patients with NMIBC treated with BCG intravesical immunotherapy, the overexpression of ki67 was the risk factor for PFS, and the relationship between the expression of ki67 and RFS had no statistical significance. In Caucasians, the overexpression of ki67 was the risk factor for RFS and PFS. However, these findings still need well-designed, prospective, randomized controlled trials with a large sample size for validation.

For peer review only - http://bmjopen.bmj.com/site/about/guidelines.xhtml

Key words: Ki67; meta-analysis; non-muscular-invasive bladder cancer; prognosis

For peer review only

For peer review only - http://bmjopen.bmj.com/site/about/guidelines.xhtml

Strengths and limitations of this study

This meta-analysis and systematic review was performed by a strictly literature search and was the first meta-analysis to evaluate the prognostic value of ki67 on patients with NMIBC after transurethral resection and BCG intravesical immunotherapy.

The review found the Prognostic Value of ki67 in BCG-Treated NMIBC, which can guide the follow-up immunohistochemical markers research.

The review only included English published studies and did not consider the surgical skills from published studies.

For peer review only

For peer review only - http://bmjopen.bmj.com/site/about/guidelines.xhtml

Introduction

Bladder cancer is one of the most common clinical urological tumor and is a direct threat to the survival of patients with the disease. The incidence of bladder cancer varies across the world with the highest rate in the developed communities.[1] A total of 429,800 new cases of bladder cancer and 165,100 deaths occurred in 2012 worldwide. The majority of bladder cancer occurs in men, and about a tenfold variation has been reported in incidence rates internationally.[2] About 70% of these patients are non-muscle-invasive bladder cancer (NMIBC).[3] Four major organizational guidelines on NMIBC, including the American Urological Association/Society of Urologic Oncology, European Association of Urology, National Comprehensive Cancer Network, and National Institute for Health and Care Excellence guidelines, recommend that the proper initial transurethral resection (TUR) of bladder tumor is a critical step in the initial management and staging of the disease.[4] However, TUR surgery alone cannot solve the postoperative problems for NMIBC because of high recurrence rate and disease development.[5] Postoperative TUR associated with Bacillus Calmette–Guérin (BCG) intravesical immunotherapy can prevent the postoperative recurrence of NMIBC and significantly reduce the moderate and high development risk of NMIBC.[6, 7] However, the postoperative BCG intravesical immunotherapy still has some problems. The failure rate of BCG intravesical therapy in NMIBC is about 40%–50%.[8] Furthermore, BCG also has toxic side effects, such as hepatitis, pneumonitis, epididymitis/orchitis, abscess formation, bladder contracture, ureteral obstruction, BCG sepsis, leukopenia, and hematuria.[9] Therefore, BCG therapy should be individually performed, and the patients having no effectiveness on BCG therapy should be timely recognized. These patients or those with poor prognosis should receive radical cystectomy or other therapy in time to avoid futile

For peer review only - http://bmjopen.bmj.com/site/about/guidelines.xhtml

treatment and alleviate pain. However, the recognition of patients with no effect of TUR postoperative BCG intravesical immunotherapy is still hard due to the heterogeneity of bladder cancer and individuality of patients.[10] Therefore, finding the prognostic factors for patients with NMIBC receiving TUR and BCG therapy is extremely necessary.

The recurrence rate of bladder cancer treated with different therapies is between 50% and 80%, and about 15% of the low-grade tumor recurrence involves high-grade tumors.[11] The patients need periodical cystoscopy to find the recurrent focus in time. A reliable prognostic molecular marker can reduce the pain caused by cystoscopy. Because NMIBC does not have reliable prognostic markers, it is hard to decide postoperative therapy in the clinic,[12] which depends mainly on the clinical guidelines and physician's experience. Currently, some of the published studies about immunohistochemical markers have evaluated the prognostic value of BCG intravesical immunotherapy on the patients first receiving TUR. The main immunohistochemical markers include ki67, p53, p27, pRb, CD9, CD20, E2F1, and so forth.[13, 14] However, no immunohistochemical marker has been confirmed so far. The prognostic value of ki67 antigen on the survival in patients with NMIBC receiving BCG intravesical immunotherapy has been controversial. For example, Kruger.[15] reported that ki67 antigen was an independent predictive factor of recurrence in pT1 stage tumor, but Oderde[16] believed that ki67 was an independent predictive factor for all the NMIBC recurrence. Zlotta[17] reported that ki67 antigen had no independent prognostic value in patients receiving BCG therapy. Saint[18] retrospected the recent 25-year published studies and believed that the independent prognostic factor for bladder cancer on BCG response was not unclear. An international consensus group listed various bladder cancer prognostic indexes by

For peer review only - http://bmjopen.bmj.com/site/about/guidelines.xhtml

reviewing PubMed and considered that although some markers (such as ki67 and p53) were possible to predict the recurrence and development of bladder cancer, the data still had heterogeneity. Thus, strict test criteria and clear statistical methods should be established for further evaluation.[19]

A meta-analysis can enlarge the sample size by integrating independent studies with small sample size, further increase the statistical efficacy, and reduce the wrong conclusion caused by the small sample size.[20] The aim of this study was to explore the prognostic value of ki67 as a marker in BCG-treated NMIBC. Based on the literature search, this study was the first meta-analysis to evaluate the prognostic value of ki67 on patients with NMIBC after BCG therapy.

Methods

This meta-analysis was performed according to the Preferred Reporting Items for Systematic Reviews and Meta-Analyses (PRISMA) Statement (**Table S1**).[21] Because all the enrolled published studies were approved by the ethics committee in the research institute, the present meta-analysis did not need the approval.

Literature retrieval strategy

Published studies were retrieved from Web of Science, PubMed, Cochrane Library, and Embase databases. Free word retrieval strategy was used. The search terms were "bladder cancer or bladder carcinoma or bladder neoplasm or bladder tumor," "Bacillus Calmette–Guérin or BCG," and "ki67 antigen or ki-67 or ki67 or MBI-1." The retrieval time was until May 24, 2017, assisted with manual retrieval. The enrolled contents included the reference and relevant suggestive references while searching.

Inclusion and exclusion criteria

For peer review only - http://bmjopen.bmj.com/site/about/guidelines.xhtml

Inclusion criteria were as follows: (1) prospective studies or retrospective research published studies evaluating the prognostic relationship between the expression of ki67 and NMIBC treated with BCG; (2) the expression of ki67 in tissues detected by immunohistochemistry; (3) hazard ratio (HR) and 95% confidence interval (95% CI) directly obtained from the published studies; and (4) published English studies. Exclusion criteria were as follows: (1) review, systematic evaluation, case report, editorial, and specialist experience; (2) no human subjects; and (3) published studies in which data could not be extracted or those having wrong data.

Data extraction and evaluation of literature quality

Based on the aforementioned criteria, two reviewers independently screened the published studies by reading titles and abstracts and got preliminary conclusions. If the conclusions were not consistent, the literature was discussed by all the authors to decide its enrollment. The relevant information of the enrolled published studies was extracted, such as first author, publication time, research country, sex, case number, age, follow-up date, disease stage, cutoff values, RFS, and PFS. The Newcastle–Ottawa Scale (NOS) was used to evaluate the quality of all the published studies,[22] scores 0–3, 4–5, and 6–8 were accepted as low, medium, and high quality.

Statistical methods

The study effects of recurrence-free survival (RFS) and progression-free survival (PFS) were reflected by 95% CI and HR. The influence of the expression of ki67 on prognosis was expressed as 95% CI and HR. The values of HR and 95% CI were directly obtained from the original published studies. Besides, the Parmar and Tierney's[23] method was used to extract the data because some of the published studies did not directly provide HR and 95% CI. For example, some studies provided only the survival curve. In this meta-analysis, the DerSimonian–Laird random-effects model[24]

For peer review only - http://bmjopen.bmj.com/site/about/guidelines.xhtml

was used because when the heterogeneity was large, only the random-effects model could be suitable used. Similar to traditional methods, HR ＞1 was considered as the prognostic risk factor for the overexpression of ki67, and HR ＜1 was a protective factor. 95% CI ＜1 indicated a statistical difference in the relationship between the overexpression of ki67 and prognosis.

The heterogeneity was calculated according to chi-square-based Q test and I^2 statistic.[25] The heterogeneity was judged by the I^2 value (low heterogeneity: I^2 ＜ 25%; moderate heterogeneity: I^2 = 25%–50%; large heterogeneity: I^2 ＞50%). Besides, a *P* value >0.05 was also considered as low heterogeneity. Then, the subgroup analysis based on regions, sample size, follow-up period, tumor grading, cutoff value, publication time, and patient age was performed. A Galbraith plot was used to search published studies with heterogeneity,[26] and after excluding these published studies, the meta-analysis was performed again. Meanwhile, the factors causing heterogeneity were also explored by the residual maximum likelihood (REML)-based random-effects meta-regression analysis.[27] All the statistical analyses were performed using the Stata12.0 software (StataCorp, TX, USA), and the two-sided test was used to evaluate the *P* value.

Evaluation of publication bias

Begg’s plot and Egger's test method were used to find the possible publication bias. A *P* value <0.05 was believed to have publication bias.

Results

For peer review only - http://bmjopen.bmj.com/site/about/guidelines.xhtml

Literature screening

A total of 97 published studies were retrieved. Furthermore, 68 of them were excluded after duplicates removed and records screened, and 18 were excluded after reading the full text (10 published studies from which HR and 95% CI could not be obtained, 2 non-English studies, and 6 that did not use ki67 detection). Finally, 11 published studies were enrolled in the meta-analysis (**Figure 1**).

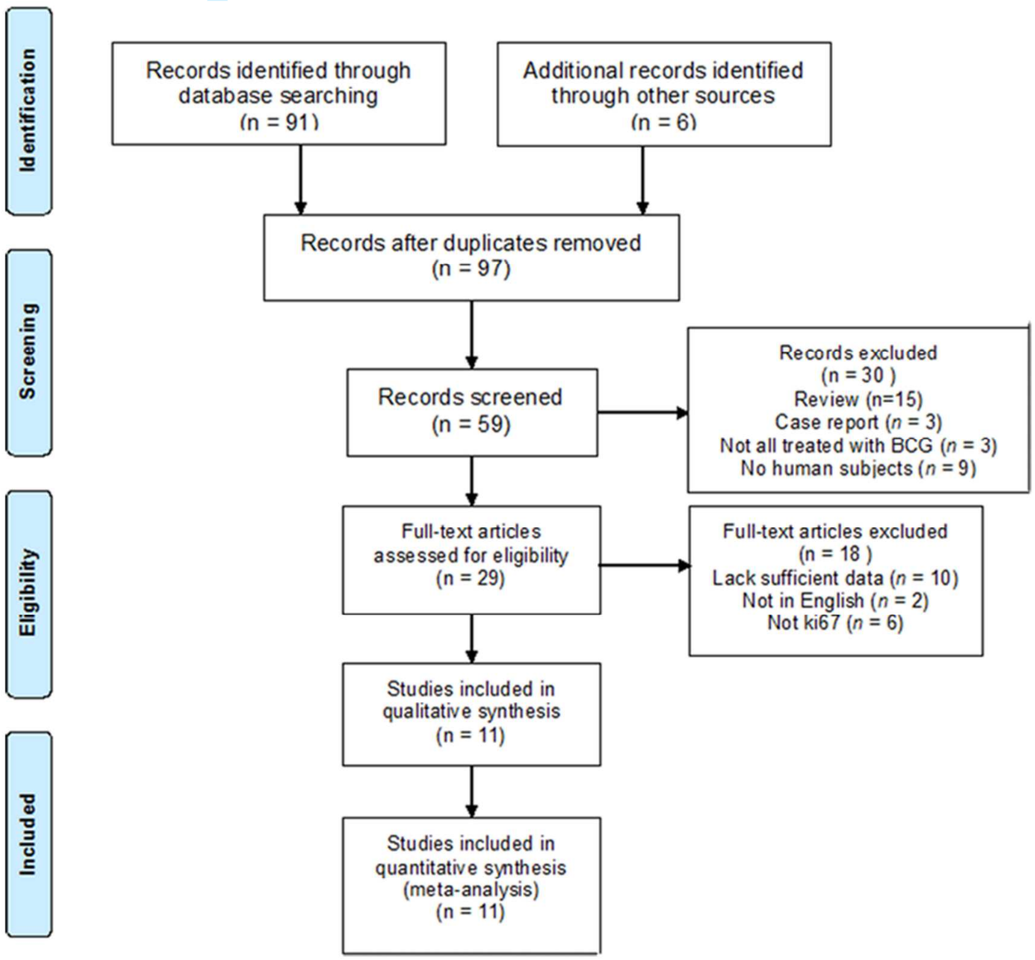

Figure 1. Flow diagram of study selection.

Basic characteristics and quality evaluation of enrolled published studies

The enrolled 11 published studies were published between 1997 and 2013, and the

For peer review only - http://bmjopen.bmj.com/site/about/guidelines.xhtml

countries included Italy, South Korea, Spain, Germany, New Zealand, Canada, Portugal, and France. The largest sample size was 309, and the smallest one was 32. A total of 1321 patients were enrolled in this study. The follow-up period was beyond 36 months, and the longest was 229 months. T1 was the main tumor grading, and the cutoff value ranged from 10.4% to 40%. Seven published studies reported patients' RFS, and nine reported PFS (**Table 1**). One literature was scored as 6 star by NOS, seven as 7 star, and three as 8 star, and the median of the NOS score was 7 (**Table 2**).

For peer review only

For peer review only - http://bmjopen.bmj.com/site/about/guidelines.xhtml

Table 1. Main characteristics of all studies included in this meta-analysis

Study	Year	Country	Male/Female	No. of patients	Age (year)	Follow-up (month)	Stage	Cutoff	Survival analysis
Oderda[16]	2013	Italy	166/26	192	73.2 (SD 11.9)	100 (2–229)	All NMIBC	20%	RFS
Park[14]	2013	Korea	53/8	61	66 (31–85)	60 (6–217)	T1G3	10.4%	RFS/PFS
Quintero[28]	2013	Spain	143/21	164	61 (29–93)	75 (60–144)	Ta	13%	PFS
Bertz[12]	2012	Germany	237/72	309	71.7 (38–87)	49 (5–172)	pT1	15%	RFS/PFS
van Rhijn[29]	2012	Netherlands, Canada	105/24	129	68.8 (SD 9.9)	78 (39.6-110.4)	T1	25%	RFS/PFS
Burger[30]	2007	Germany	45/21	71	71 (52–94)	39 (1–133)	T1/Ta	15%	RFS
Queipo-zaragoza[31]	2007	Spain	71/12	83	68.1 (SD 8.5)	All >36	T1G3	40%	PFS
Lopez-Beltran[32]	2004	Spain	49/2	51	69.96 (49–89)	63.82 (60–144)	T1G3	13%	PFS
Santos[33]	2003	Portugal	115/44	159	66 (21–88)	46.5 (4–123)	pTa/pT1	18%	RFS/PFS
Blanchet[34]	2001	France	-	70	62.6 (21–84)	64 (12–111)	pT1/pTa	13%	PFS
Lee[35]	1997	Korea	28/4	32	57.1 (30–81)	All >24	T1G2-3	20%	RFS

No., number; NMIBC, non-muscle-invasive bladder cancer; RFS, recurrence-free survival; PFS, progression-free survival

For peer review only - http://bmjopen.bmj.com/site/about/guidelines.xhtml

Table 2. Quality of the included studies assessed by NOS

Study	Selection				Comparability	Exposure			Scores
	Adequate definition of cases	Representativeness of cases	Selection of controls	Definition of controls	Control for important factor	Ascertainment of exposure	Same method to ascertain for cases and controls	Non-response rate	
Oderda[16]	—	☆	☆	☆	☆☆	☆	☆	☆	8
Park[14]	—	☆	☆	—	☆☆	☆	☆	☆	7
Quintero[28]	—	☆	☆	—	☆☆	☆	☆	—	6
Bertz[12]	—	☆	☆	—	☆☆	☆	☆	☆	7
van Rhijn[29]	—	☆	☆	—	☆☆	☆	☆	☆	7
Burger[30]	☆	☆	☆	—	☆☆	☆	☆	—	7
Queipo-zaragoza[31]	—	☆	☆	—	☆☆	☆	☆	☆	7
Lopez-Beltran[32]	—	☆	☆	☆	☆☆	☆	☆	☆	8
Santos[33]	—	☆	☆	—	☆☆	☆	☆	☆	7
Blanchet[34]	—	☆	☆	☆	☆☆	☆	☆	☆	8
Lee[35]	—	☆	☆	—	☆☆	☆	☆	☆	7

For peer review only - http://bmjopen.bmj.com/site/about/guidelines.xhtml

Influence of the expression of ki67 on RFS

Seven published studies reported ki67 expression and PFS results of patients with NMIBC receiving BCG. The meta-analysis indicated that ki67 had no statistical significance with RFS (HR = 1.331, 95% CI: 0.980–1.809), and no heterogeneity among the enrolled studies was reported (I^2 = 36.7%, P = 0.148) (**Figure 2A**). The subgroup analysis was performed based on the regions, sample size, follow-up period, stage, cutoff value, publication time, and age. The results indicated that in the Caucasian subgroup, the overexpression of ki67 was the risk factor for RFS (HR = 1.441, 95% CI: 1.014–2.047). In the subgroup with a follow-up period shorter than 6 months, the overexpression of ki67 was the risk factor for RFS (HR = 1.853, 95% CI: 1.316–2.607) (**Table 3**).

For peer review only - http://bmjopen.bmj.com/site/about/guidelines.xhtml

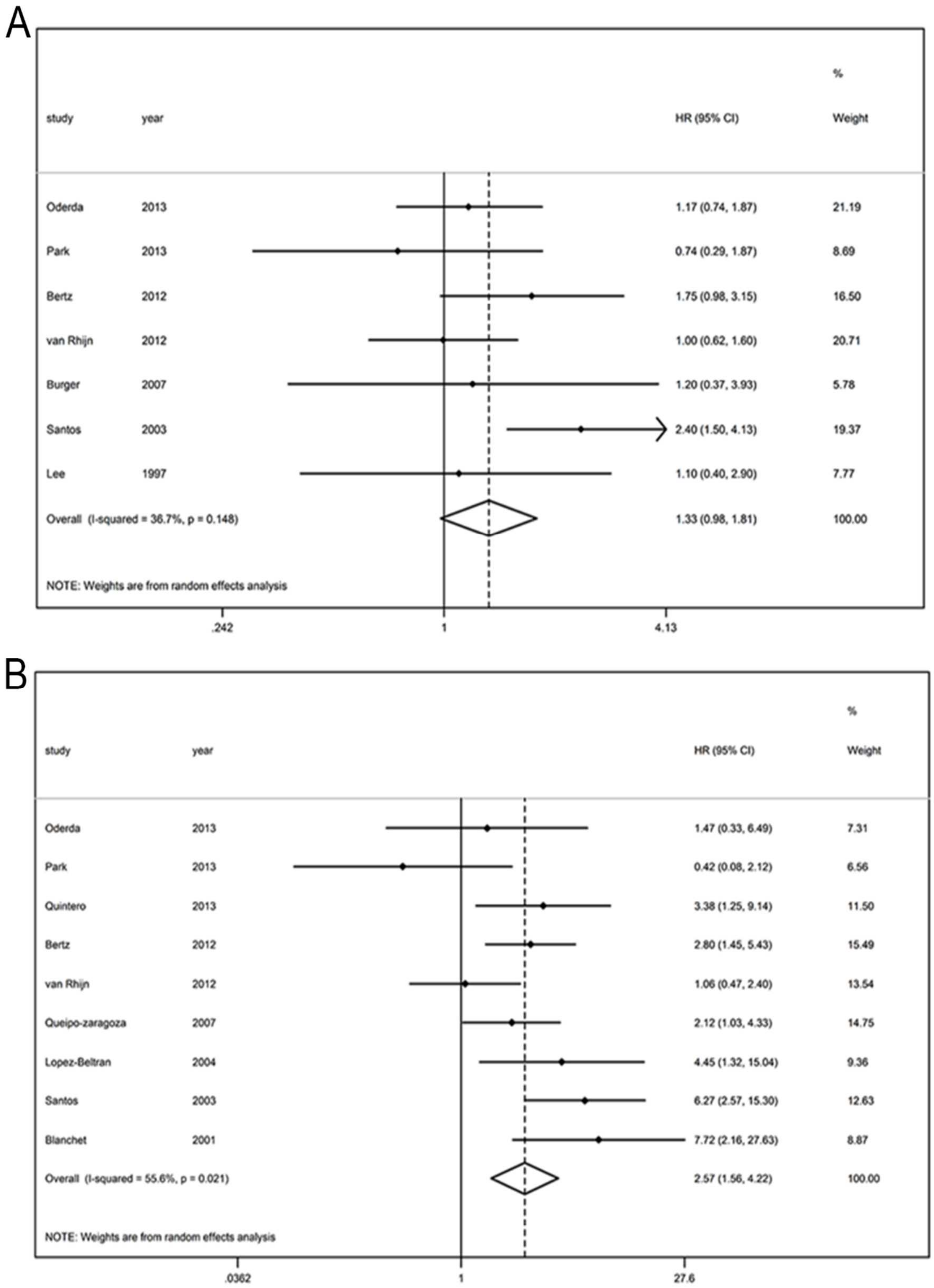

Figure 2. Forest plots of HRs estimated for the relationship between the expression of ki67 and RFS (A) or PFS (B) among patients with NMIBC treated with BCG.

For peer review only - http://bmjopen.bmj.com/site/about/guidelines.xhtml

Table 3. Subgroup results of RFS and heterogeneity test

Variables	Study number	HR (95% CI)	Heterogeneity test		
			Q	*P*	I^2 (%)
Total RFS	7	1.331 (0.980–1.809)	9.48	0.148	36.7
Region					
Asian	2	0.892 (0.454–1.752)	0.32	0.570	0.0
Caucasian	5	1.441 (1.014–2.047)	7.52	0.111	46.8
Sample size					
>100	4	1.466 (0.986–2.181)	7.44	0.059	59.7
≤100	3	0.959 (0.534–1.725)	0.51	0.777	0.0
Follow-up (month)					
≥60	3	1.036 (0.758–1.415)	0.79	0.674	0.0
<60	4	1.853 (1.316–2.607)	2.62	0.453	0.0
Stage					
All NMIBC	3	1.575 (0.915–2.711)	4.44	0.109	54.9
Others	4	1.153 (0.821–1.620)	3.21	0.360	6.6
Cut off					
15%	2	1.625 (0.963–2.743)	0.31	0.575	0.0
Others	5	1.252 (0.839–1.869)	8.56	0.073	53.3
Patient age (year)					
≥70	3	1.352 (0.955–1.913)	1.16	0.559	0.0
<70	4	1.256 (0.717–2198)	8.32	0.040	63.9

NMIBC, non-muscle-invasive bladder cancer; RFS, recurrence-free survival.

For peer review only

For peer review only - http://bmjopen.bmj.com/site/about/guidelines.xhtml

Influence of the expression of ki67 on PFS

A total of nine published studies reported ki67 expression and PFS results of patients with NMIBC receiving BCG. The meta-analysis indicated that ki67 had no statistical significance with RFS (HR = 2.567, 95% CI: 1.562–4.219), and the overexpression of ki67 was the risk factor for PFS. Statistical heterogeneity was found among all the included studies (I^2 = 55.6%, P = 0.021) (**Figure 2B**). The subgroup analysis was performed based on the regions, sample size, follow-up period, stage, cutoff value, publication time, and age. The results indicated that the overexpression of ki67 was the risk factor for PFS in the Caucasian subgroup (HR = 2.883, 95% CI: 1.830–4.544), the subgroup with sample size >100 (HR = 2.559, 95% CI: 1.372–4.774), the subgroup with the follow-up period < 6 months (HR = 3.158, 95% CI: 1.774–5.623), the subgroup with other cutoffs (HR = 2.515, 95% CI: 1.382–4.576), and the two subgroups based on age (HR = 2.800, 95% CI: 1.447–5.418 and HR = 2.654, 95% CI: 1.381–5.100, respectively) (**Table 4**).

For peer review only - http://bmjopen.bmj.com/site/about/guidelines.xhtml

Table 4. Subgroup results of PFS and heterogeneity test

Variables	Study number	HR (95% CI)	Heterogeneity test		
			Q	*P*	I^2 (%)
Total PFS	9	2.567 (1.562–4.219)	18.1	0.021	55.6
Region					
Asian	1	0.421 (0.084–2.114)	0.00		
Caucasian	8	2.883 (1.830–4.544)	12.99	0.072	46.1
Sample size					
>100	5	2.559 (1.372–4.774)	9.26	0.055	56.8
≤100	4	2.536 (0.943–6.818)	8.75	0.033	65.7
Follow-up (month)					
≥60	6	2.153 (0.984–4.710)	13.08	0.023	61.8
<60	3	3.158 (1.774–5.623)	3.56	0.169	43.8
Stage					
All NMIBC	3	4.673 (1.938–11.264)	3.29	0.193	39.2
Others	6	2.044 (1.213–15.040)	9.58	0.088	47.8
Cut off					
15%	1	2.800 (1.447–5.418)	0.00		
Others	8	2.515 (1.382–4.576)	17.92	0.012	60.9
Patient age (year)					
≥70	2	2.519 (1.377–4.606)	0.60	0.438	0.0
<70	7	2.654 (1.381–5.100)	17.40	0.008	65.5

NMIBC, non-muscle-invasive bladder cancer; PFS, progression-free survival.

For peer review only

Galbraith plot

Using Galbraith plot (**Figure 3A**), it was found that Santos[33] was the main reason for the heterogeneity of RFS. After the aforementioned study was excluded, the rest RFS studies had no significant heterogeneity according to the new meta-analysis (I^2 = 0.0%, P = 0.667). However, the expression of ki67 still had no statistical significance with RFS (HR = 1.161, 95% CI: 0.896–1.504) (**Figure S1**). Using the Galbraith plot (**Fig. 3B**), it was found that Santos,[33] Park,[14] and van Rhijn[29] were the main reason for the heterogeneity of PFS. After these studies were excluded, the remaining RFS studies had no significant heterogeneity according to the new meta-analysis (I^2 = 0.0%, P = 0.497). The expression of ki67 still had statistical significance with PFS (HR = 2.922, 95% CI: 2.002–4.266) (**Figure S2**).

For peer review only

For peer review only - http://bmjopen.bmj.com/site/about/guidelines.xhtml

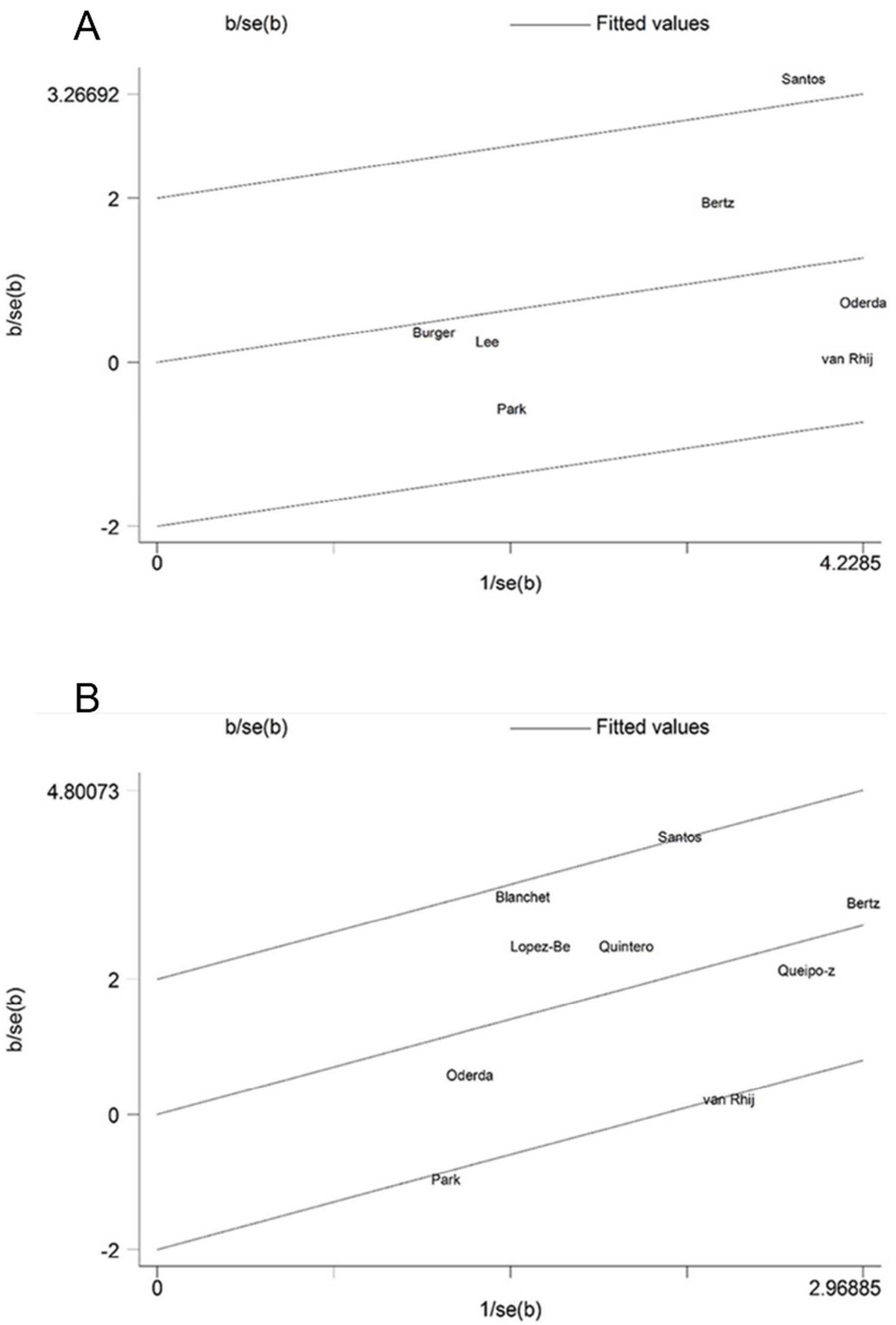

Figure 3. Galbraith plot analysis was used to evaluate heterogeneity. It suggested that two studies were the potential source of heterogeneity for RFS (A), while one was the source of heterogeneity for PFS (B).

Meta-regression analysis

The meta-regression analysis indicated that the factors influencing heterogeneity (publication time, research region, sample size, stage, cutoff value, age, and follow-up period) might not be the reason for RFS heterogeneity. Publication time was the reason for heterogeneity of PFS (P = 0.036), but other factors were not (**Table S2**).

Publication bias

The funnel plot (**Figure 4A**) was basically symmetrical for RFS. The results of Begg's test and Egger's test showed P (Begg's) = 0.761, P (Egger's) = 0.601. The funnel plot (**Figure 4B**) was also basically symmetrical for PFS. The results of Begg's test and Egger's test showed P (Begg's) = 0.917, P (Egger's) = 0.964.

For peer review only

For peer review only - http://bmjopen.bmj.com/site/about/guidelines.xhtml

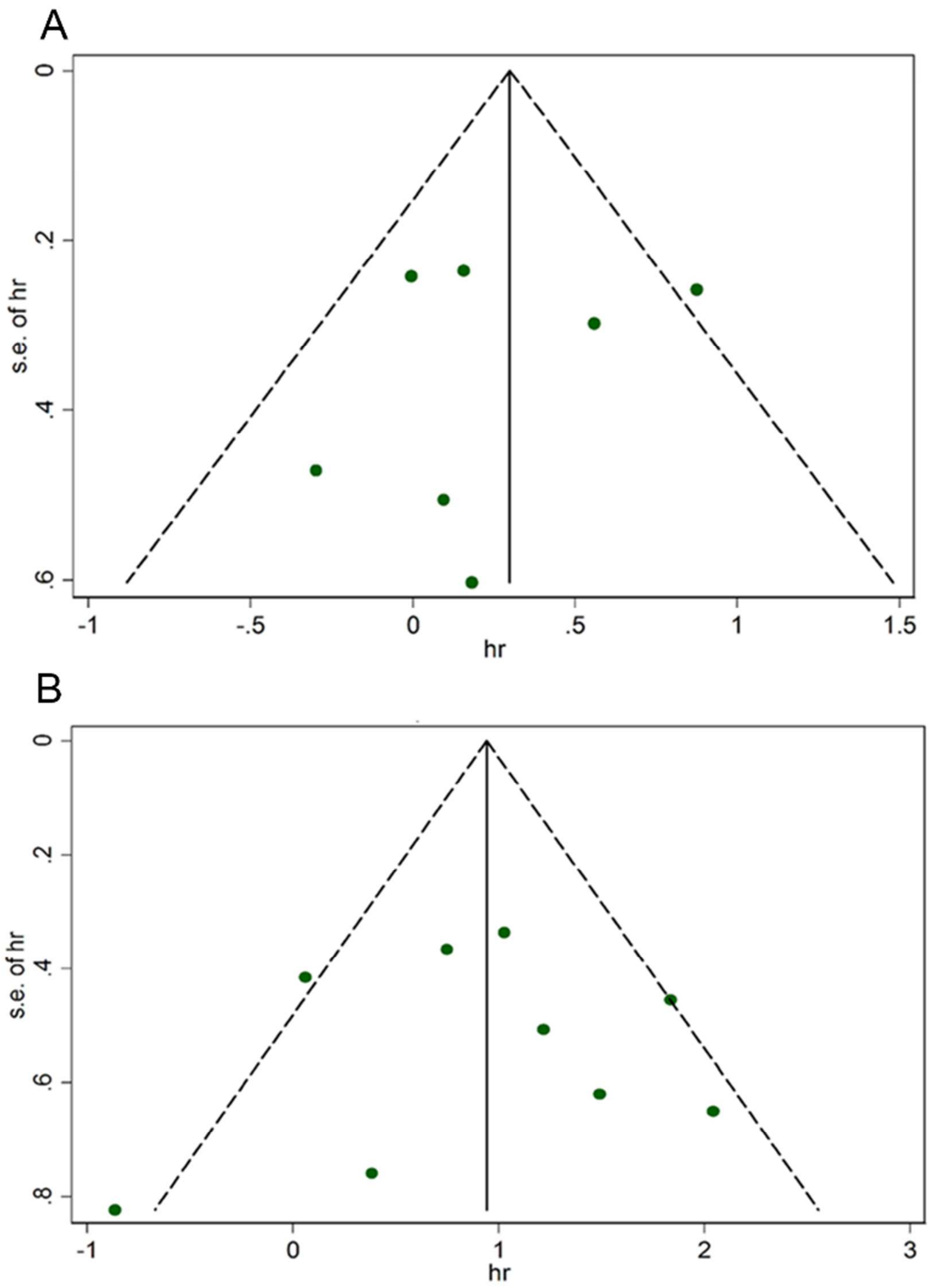

Figure 4. Funnel plots of the expression of ki67 and RFS (A) or PFS (B).

Discussion

A total of 11 published studies with 1321 cases complying with the inclusion criteria

For peer review only - http://bmjopen.bmj.com/site/about/guidelines.xhtml

were enrolled in this meta-analysis. The results of the meta-analysis indicated that the expression of ki67 had no statistical significance with RFS, but it had significance with PFS. The overexpression of ki67 was the risk factor for PFS. It suggested that ki67 was the prognostic predictive marker for patients with NMIBC after BCG therapy. Besides, the aforementioned conditions did not change after excluding the published studies possibly causing heterogeneity and reperforming the meta-analysis. It further proved that the result of the aforementioned meta-analysis was stable, that is, the overexpression of ki67 was the risk factor for PFS. In the Caucasian subgroup, the overexpression of ki67 was the risk factor for PFS and RFS, suggesting the racial classification and regional factor might play important roles in the prognosis of patients with NMIBC after BCG therapy. In the two subgroups based on age, the overexpression of ki67 was the risk factor for PFS, suggesting that age was the important factor influencing the prognosis of bladder cancer. The elder the patient, the worse the prognosis would be. Besides, the meta-regression analysis indicated publication time as the reason for PFS heterogeneity. The cumulative meta-analysis indicated that the expression of ki67 had statistical significance with RFS and PFS. It suggested that the correlation of the expression of ki67 with RFS and PFS needed further exploration to observe the following changes. Besides, according to the funnel plot, Begg's test, and Egger's test, the enrolled studies had no significant publication bias. Thus, the reliability of the present meta-analysis was high.

In 2016, the European Association of Urology (EUA)[36] recommended a scoring system for the prognostic evaluation of NMIBC based on six clinical and pathological factors built by European Organization for the Research and Treatment of Cancer-Genito-Urinary Cancer Group (EORTC), including number of tumors, tumor size, prior recurrence rate, T category, presence of concurrent carcinoma in situ, and

For peer review only - http://bmjopen.bmj.com/site/about/guidelines.xhtml

tumor grade (**Table S3**). The patients were categorized into low-risk tumors, intermediate-risk tumors, and high-risk tumors using this assessment system to evaluate the prognosis. For the patients after BCG therapy, the EUA recommended another risk calculator developed by the Club Urologico Espanol de Tratamiento Oncologico (CUETO) and the EORTC. This calculator based on gender, age, recurrent tumor, number of tumors, T category, associated Tis, and grade. The CUETO risk calculator can be achieved at http://www.aeu.es/Cueto.html. For the two scales for patients with NMIBC, no matter used alone or combined, the recommended level was B grade. The two scales could be used together in the clinic. When using the CUETO scale, the calculated recurrent risk was lower than that from the EORTC scale,[37] which might be related to the special design in the CUETO scale for the patients receiving BCG intravesical immunotherapy. However, the scoring system only depending on clinical and pathological factors could not accurately evaluate the prognosis of bladder cancer patients in T1 stage due to the independence of disease condition in each patient.[38] The markers regulated at the genetic level may judge the prognosis of bladder cancer patients with the development of precision medicine. A reliable marker helps in recognizing the patients who failed in BCG intravesical immunotherapy with high risk in time. Hence, these patients can undergo radical cystectomy or other treatments in time. Unfortunately, no prognostic marker has been applied in clinic currently.

Ki67 is a nucleoprotein that can be detected in the cell cycles except G0 phase.[39] The expression of human ki67 protein is closely related to proliferation. Therefore, it is an ideal marker to confirm the growth fraction of specific cell colony.[40] Ki67 is a widely known amplified biomarker. The ki67 monoclonal antibody can be detected by the immunohistochemical method.[41] Ki67 has been proved to be a good proliferation

For peer review only - http://bmjopen.bmj.com/site/about/guidelines.xhtml

marker in different cancers, including bladder cancer.[42]

So far, some meta-analyses have studied the effect of ki67 on the prognostic quality of life of esophageal cancer, breast cancer, epithelial ovarian cancer, and so on.[43-45] Some studies have also focused on the other aspects of bladder cancer. Using meta-analysis, Luo[46] believed that a high reactivity of ki67 could predict the poor prognosis in patients with bladder cancer. The univariate analysis showed that cancer-specific survival, disease-free survival, overall survival , PFS, and RFS had a significant correlation with poor prognosis in patients with a high reactivity of ki67. However, this study enrolled all types of bladder tumors and all the therapies for NMIBC. Currently, the treated bladder cancer in the clinic is mainly NMIBC. Thus, most of the applied therapy is TUR combined with installations of chemotherapy or BCG intravesical immunotherapy based on the patients' conditions. Therefore, this analysis had a certain limitation in the prognosis judgment on the patients with NMIBC after BCG intravesical immunotherapy.

Currently, few evidence-based studies focused on the prognosis of patients with NMIBC after BCG intravesical immunotherapy. Using meta-analysis, Zhou[47] analyzed the correlation between the expression of p53 and quality of life of patients with NMIBC after BCG intravesical immunotherapy. They believed that the overexpression of p53 in patients with NMIBC treated with BCG might be associated with RFS, especially in Asian population. Similarly, Du[48] also performed the meta-analysis on the relationship between p53 status and NMIBC in T1 stage and believed that the overexpression of p53 might be related to the development of NMIBC. The present study indicated that the overexpression of ki67 was the risk factor for PFS, but the expression of ki67 had no statistical significance with RFS. For

For peer review only - http://bmjopen.bmj.com/site/about/guidelines.xhtml

Caucasians, the overexpression of ki67 was the risk factor for PFS and RFS. P53 is the most common inactivated tumor suppressor gene in tumor cells.[49] The inactivation of p53 may cause cell abnormal hyperplasia and cancerization. The variation in p53 results in enhanced proliferation, invasion, and metabolism.[50] The increase in the expression of ki67 ，as cell proliferation marker ，suggests enhanced proliferation.[40] As a tumor suppressor gene with complicated function, the range of effects of p53 is wider. The accuracy in the prediction of quality of life may not be more appropriate compared with ki67. The genetic difference between Asians and Caucasians suggests that different prediction systems should be built for different races. Besides, p27, E2F1, ezrin, and CK20 were also studied in other investigations for predicting NMIBC prognosis, which could be further explored comparing their advantages used alone or combined.

However, this study still had some limitations. First, some of the enrolled published studies were retrospective studies, involving different populations, using different techniques, and with different cutoff values. All these reasons might have caused the heterogeneity. Second, the meta-analysis included English published studies. Although Begg's test and Egger's test did not suggest publication bias, this study could still be influenced by the bias. Finally, the surgical skills were different in different published studies, which might affect effectiveness judgment of BCG.

Conclusions

For the patients with NMIBC treated with BCG intravesical immunotherapy, the overexpression of ki67 was the risk factor for PFS, the overexpression of ki67 was the risk factor for PFS, but the relationship between ki67 expression and PFS had no

For peer review only

statistical significance. In the Caucasian subgroup, the overexpression of ki67 was the risk factor for PFS and RFS. Owing to the aforementioned limitations of the present study, RCTs with large sample size are still required to validate the results.

Contributors

Conceived and designed the experiments: YHH NW XFZ. Extracted the data: XC YSD ZSD JFW YHH NW XFZ. Analyzed the data: YHH NW XFZ. Contributed reagents/materials/analysis tools: ZSD JFW XC. Wrote the paper: YHH NW. Critically revised the report: XFZ.

Funding

This study was supported by grants from the International S&T Cooperation Program of China (ISTCP) (Grant No.2014DFE30010).

Disclaimer

Its contents are solely the responsibility of the author. The funders had no role in study design, data collection and analysis, decision to publish, or preparation of the manuscript.

Competing interests

None declared.

Data sharing statement

No additional data available

For peer review only - http://bmjopen.bmj.com/site/about/guidelines.xhtml

Reference

1. Ploeg M, Aben KK, Kiemeney LA. The present and future burden of urinary bladder cancer in the world. *World J Urol* 2009;**27**:289-93. doi:10.1007/s00345-009-0383-3.

2. Torre LA, Bray F, Siegel RL, *et al.* Global cancer statistics, 2012. *CA Cancer J Clin* 2015;**65**:87-108. doi:10.3322/caac.21262.

3. Ro JY, Staerkel GA, Ayala AG. Cytologic and histologic features of superficial bladder cancer. *Urol Clin North Am* 1992;**19**:435-53.

4. Woldu SL, Bagrodia A, Lotan Y. Guideline of guidelines: non-muscle-invasive bladder cancer. *BJU Int* 2017;**119**:371-80. doi:10.1111/bju.13760.

5. Brausi M, Collette L, Kurth K, *et al.* Variability in the recurrence rate at first follow-up cystoscopy after TUR in stage Ta T1 transitional cell carcinoma of the bladder: a combined analysis of seven EORTC studies. *Eur Urol* 2002;**41**:523-31.

6. Jarvinen R, Kaasinen E, Sankila A*, et al.* Long-term efficacy of maintenance bacillus Calmette-Guerin versus maintenance mitomycin C instillation therapy in frequently recurrent TaT1 tumours without carcinoma in situ: a subgroup analysis of the prospective, randomised FinnBladder I study with a 20-year follow-up. *Eur Urol* 2009;**56**:260-5. doi:10.1016/j.eururo.2009.04.009.

7. Sylvester RJ, Brausi MA, Kirkels WJ*, et al.* Long-term efficacy results of EORTC genito-urinary group randomized phase 3 study 30911 comparing intravesical instillations of epirubicin, bacillus Calmette-Guerin, and bacillus Calmette-Guerin plus isoniazid in patients with intermediate- and high-risk stage Ta T1 urothelial

For peer review only - http://bmjopen.bmj.com/site/about/guidelines.xhtml

carcinoma of the bladder. *Eur Urol* 2010;**57**:766-73. doi:10.1016/j.eururo.2009.12.024.

8. Gual Frau J, Palou J, Rodriguez O, *et al.* Failure of Bacillus Calmette-Guerin therapy in non-muscle-invasive bladder cancer: Definition and treatment options. *Arch Esp Urol* 2016;**69**:423-33.

9. Boyd LA. Intravesical Bacillus Calmette-Guerin for treating bladder cancer. *Urol Nurs* 2003;**23**:189-91, 99; quiz 92.

10. Alkhateeb SS, Neill M, Bar-Moshe S, *et al.* Long-term prognostic value of the combination of EORTC risk group calculator and molecular markers in non-muscle-invasive bladder cancer patients treated with intravesical Bacille Calmette-Guerin. *Urol Ann* 2011;**3**:119-26. doi:10.4103/0974-7796.84954.

11. Mariappan P, Smith G. A surveillance schedule for G1Ta bladder cancer allowing efficient use of check cystoscopy and safe discharge at 5 years based on a 25-year prospective database. *J Urol* 2005;**173**:1108-11. doi:10.1097/01.ju.0000149163.08521.69.

12. Bertz S, Otto W, Denzinger S, *et al.* Combination of CK20 and Ki-67 immunostaining analysis predicts recurrence, progression, and cancer-specific survival in pT1 urothelial bladder cancer. *Eur Urol* 2014;**65**:218-26. doi:10.1016/j.eururo.2012.05.033.

13. Kim K, Cho YM, Park BH, *et al.* Histological and immunohistochemical markers for progression prediction in transurethrally resected high-grade non-muscle invasive bladder cancer. *Int J Clin Exp Pathol* 2015;**8**:743-50.

14. Park J, Song C, Shin E, *et al.* Do molecular biomarkers have prognostic value in primary T1G3 bladder cancer treated with bacillus Calmette-Guerin intravesical therapy? *Urol Oncol* 2013;**31**:849-56. doi:10.1016/j.urolonc.2011.06.004.

For peer review only - http://bmjopen.bmj.com/site/about/guidelines.xhtml

15. Kruger S, Thorns C, Stocker W, *et al.* Prognostic value of MCM2 immunoreactivity in stage T1 transitional cell carcinoma of the bladder. *Eur Urol* 2003;**43**:138-45.

16. Oderda M, Ricceri F, Pisano F, *et al.* Prognostic factors including Ki-67 and p53 in Bacillus Calmette-Guerin-treated non-muscle-invasive bladder cancer: a prospective study. *Urol Int* 2013;**90**:184-90. doi:10.1159/000343431.

17. Zlotta AR, Noel JC, Fayt I, *et al.* Correlation and prognostic significance of p53, p21WAF1/CIP1 and Ki-67 expression in patients with superficial bladder tumors treated with bacillus Calmette-Guerin intravesical therapy. *J Urol* 1999;**161**:792-8.

18. Saint F, Salomon L, Quintela R, *et al.* Do prognostic parameters of remission versus relapse after Bacillus Calmette-Guerin (BCG) immunotherapy exist?. analysis of a quarter century of literature. *Eur Urol* 2003;**43**:351-60; discussion 60-1.

19. Habuchi T, Marberger M, Droller MJ, *et al.* Prognostic markers for bladder cancer: International Consensus Panel on bladder tumor markers. *Urology* 2005;**66**:64-74. doi:10.1016/j.urology.2005.08.065.

20. Button KS, Ioannidis JP, Mokrysz C, *et al.* Power failure: why small sample size undermines the reliability of neuroscience. *Nat Rev Neurosci* 2013;**14**:365-76. doi:10.1038/nrn3475.

21. Liberati A, Altman DG, Tetzlaff J, *et al.* The PRISMA statement for reporting systematic reviews and meta-analyses of studies that evaluate health care interventions: explanation and elaboration. *PLoS Med* 2009;**6**:e1000100. doi:10.1371/journal.pmed.1000100.

22. Stang A. Critical evaluation of the Newcastle-Ottawa scale for the assessment of the quality of nonrandomized studies in meta-analyses. *Eur J Epidemiol* 2010;**25**:603-5. doi:10.1007/s10654-010-9491-z.

For peer review only - http://bmjopen.bmj.com/site/about/guidelines.xhtml

23. Parmar MK, Torri V, Stewart L. Extracting summary statistics to perform meta-analyses of the published literature for survival endpoints. *Stat Med* 1998;**17**:2815-34.

24. DerSimonian R, Laird N. Meta-analysis in clinical trials. *Control Clin Trials* 1986;**7**:177-88.

25. Higgins JP, Thompson SG. Quantifying heterogeneity in a meta-analysis. *Stat Med* 2002;**21**:1539-58. doi:10.1002/sim.1186.

26. Bax L, Ikeda N, Fukui N, *et al.* More than numbers: the power of graphs in meta-analysis. *Am J Epidemiol* 2009;**169**:249-55. doi:10.1093/aje/kwn340.

27. Jin M, Cai S, Guo J, *et al.* Alcohol drinking and all cancer mortality: a meta-analysis. *Ann Oncol* 2013;**24**:807-16. doi:10.1093/annonc/mds508.

28. Quintero A, Alvarez-Kindelan J, Luque RJ, *et al.* Ki-67 MIB1 labelling index and the prognosis of primary TaT1 urothelial cell carcinoma of the bladder. *J Clin Pathol* 2006;**59**:83-8. doi:10.1136/jcp.2004.022939.

29. van Rhijn BW, Liu L, Vis AN, *et al.* Prognostic value of molecular markers, sub-stage and European Organisation for the Research and Treatment of Cancer risk scores in primary T1 bladder cancer. *BJU Int* 2012;**110**:1169-76. doi:10.1111/j.1464-410X.2012.10996.x.

30. Burger M, Denzinger S, Hartmann A, *et al.* Mcm2 predicts recurrence hazard in stage Ta/T1 bladder cancer more accurately than CK20, Ki67 and histological grade. *Br J Cancer* 2007;**96**:1711-5. doi:10.1038/sj.bjc.6603784.

31. Queipo-Zaragoza JA, Ruiz-Cerda JL, Vera-Donoso CD, *et al.* Prognostic value of p53, Ki-67, microstaging and microvessel density in pT1G3 bladder tumors: creation of risk groups for progression. *Scand J Urol Nephrol* 2007;**41**:283-9. doi:10.1080/00365590601183055.

For peer review only - http://bmjopen.bmj.com/site/about/guidelines.xhtml

32. Lopez-Beltran A, Luque RJ, Alvarez-Kindelan J, *et al.* Prognostic factors in stage T1 grade 3 bladder cancer survival: the role of G1-S modulators (p53, p21Waf1, p27kip1, Cyclin D1, and Cyclin D3) and proliferation index (ki67-MIB1). *Eur Urol* 2004;**45**:606-12. doi:10.1016/j.eururo.2003.11.011.

33. Santos L, Amaro T, Costa C, *et al.* Ki-67 index enhances the prognostic accuracy of the urothelial superficial bladder carcinoma risk group classification. *Int J Cancer* 2003;**105**:267-72. doi:10.1002/ijc.11049.

34. Blanchet P, Droupy S, Eschwege P, *et al.* Prospective evaluation of Ki-67 labeling in predicting the recurrence and progression of superficial bladder transitional cell carcinoma. *Eur Urol* 2001;**40**:169-75. doi:49768.

35. Lee E, Park I, Lee C. Prognostic markers of intravesical bacillus Calmette-Guerin therapy for multiple, high-grade, stage T1 bladder cancers. *Int J Urol* 1997;**4**:552-6.

36. Babjuk M, Bohle A, Burger M, *et al.* EAU Guidelines on Non-Muscle-invasive Urothelial Carcinoma of the Bladder: Update 2016. *Eur Urol* 2017;**71**:447-61. doi:10.1016/j.eururo.2016.05.041.

37. Fernandez-Gomez J, Madero R, Solsona E, *et al.* Predicting nonmuscle invasive bladder cancer recurrence and progression in patients treated with bacillus Calmette-Guerin: the CUETO scoring model. *J Urol* 2009;**182**:2195-203. doi:10.1016/j.juro.2009.07.016.

38. Kiemeney LA, Witjes JA, Heijbroek RP, *et al.* Predictability of recurrent and progressive disease in individual patients with primary superficial bladder cancer. *J Urol* 1993;**150**:60-4.

39. Gerdes J, Lemke H, Baisch H, *et al.* Cell cycle analysis of a cell proliferation-associated human nuclear antigen defined by the monoclonal antibody Ki-67. *J Immunol* 1984;**133**:1710-5.

For peer review only - http://bmjopen.bmj.com/site/about/guidelines.xhtml

40. Scholzen T, Gerdes J. The Ki-67 protein: from the known and the unknown. *J Cell Physiol* 2000;**182**:311-22. doi:10.1002/(sici)1097-4652(200003)182:3<311::aid-jcp1>3.0.co;2-9.

41. Gerdes J, Li L, Schlueter C, *et al.* Immunobiochemical and molecular biologic characterization of the cell proliferation-associated nuclear antigen that is defined by monoclonal antibody Ki-67. *Am J Pathol* 1991;**138**:867-73.

42. Rodriguez-Alonso A, Pita-Fernandez S, Gonzalez-Carrero J, *et al.* p53 and ki67 expression as prognostic factors for cancer-related survival in stage T1 transitional cell bladder carcinoma. *Eur Urol* 2002;**41**:182-8; discussion 88-9.

43. McCormick Matthews LH, Noble F, Tod J, *et al.* Systematic review and meta-analysis of immunohistochemical prognostic biomarkers in resected oesophageal adenocarcinoma. *Br J Cancer* 2015;**113**:107-18. doi:10.1038/bjc.2015.179.

44. Gui Y, Xu S, Yang X, *et al.* A meta-analysis of biomarkers for the prognosis of triple-negative breast cancer patients. *Biomark Med* 2016;**10**:771-90. doi:10.2217/bmm-2015-0064.

45. Qiu HL, Deng SZ, Li C, *et al.* High expression of KIF14 is associated with poor prognosis in patients with epithelial ovarian cancer. *Eur Rev Med Pharmacol Sci* 2017;**21**:239-45.

46. Luo Y, Zhang X, Mo M, *et al.* High Ki-67 Immunohistochemical Reactivity Correlates With Poor Prognosis in Bladder Carcinoma: A Comprehensive Meta-Analysis with 13,053 Patients Involved. *Medicine (Baltimore)* 2016;**95**:e3337. doi:10.1097/md.0000000000003337.

47. Zhou X, Zhang G, Tian Y. p53 Status correlates with the risk of recurrence in non-muscle invasive bladder cancers treated with Bacillus Calmette-Guerin: a

For peer review only - http://bmjopen.bmj.com/site/about/guidelines.xhtml

meta-analysis. *PLoS One* 2015;**10**:e0119476. doi:10.1371/journal.pone.0119476.

48. Du J, Wang SH, Yang Q, *et al.* p53 status correlates with the risk of progression in stage T1 bladder cancer: a meta-analysis. *World J Surg Oncol* 2016;**14**:137. doi:10.1186/s12957-016-0890-9.

49. Fridman JS, Lowe SW. Control of apoptosis by p53. *Oncogene* 2003;**22**:9030-40. doi:10.1038/sj.onc.1207116.

50. Muller PA, Vousden KH, Norman JC. p53 and its mutants in tumor cell migration and invasion. *J Cell Biol* 2011;**192**:209-18. doi:10.1083/jcb.201009059.

For peer review only

For peer review only - http://bmjopen.bmj.com/site/about/guidelines.xhtml

Supplementary File

Table S1. PRISMA

Table S2. Meta-regression analysis of RFS and PFS

Table S3. Risk group stratification in NMIBC

Figure S1. Forest plots of HRs estimated for the relationship between the expression of ki67 and RFS after the aforementioned study was excluded.

Figure S2. Forest plots of HRs estimated for the relationship between the expression of ki67 and PFS after the aforementioned study was excluded

For peer review only

For peer review only - http://bmjopen.bmj.com/site/about/guidelines.xhtml

Table 1. Main characteristics of all studies included in this meta-analysis

Study	Year	Country	Male/Female	No. of patients	Age (year)	Follow-up (month)	Stage	Cutoff	Survival analysis
Oderda[16]	2013	Italy	166/26	192	73.2 (SD 11.9)	100 (2–229)	All NMIBC	20%	RFS
Park[14]	2013	Korea	53/8	61	66 (31–85)	60 (6–217)	T1G3	10.4%	RFS/PFS
Quintero[28]	2013	Spain	143/21	164	61 (29–93)	75 (60–144)	Ta	13%	PFS
Bertz[12]	2012	Germany	237/72	309	71.7 (38–87)	49 (5–172)	pT1	15%	RFS/PFS
van Rhijn[29]	2012	Netherlands, Canada	105/24	129	68.8 (SD 9.9)	78 (39.6-110.4)	T1	25%	RFS/PFS
Burger[30]	2007	Germany	45/21	71	71 (52–94)	39 (1–133)	T1/Ta	15%	RFS
Queipo-zaragoza[31]	2007	Spain	71/12	83	68.1 (SD 8.5)	All >36	T1G3	40%	PFS
Lopez-Beltran[32]	2004	Spain	49/2	51	69.96 (49–89)	63.82 (60–144)	T1G3	13%	PFS
Santos[33]	2003	Portugal	115/44	159	66 (21–88)	46.5 (4–123)	pTa/pT1	18%	RFS/PFS
Blanchet[34]	2001	France	-	70	62.6 (21–84)	64 (12–111)	pT1/pTa	13%	PFS
Lee[35]	1997	Korea	28/4	32	57.1 (30–81)	All >24	T1G2-3	20%	RFS

No., number; NMIBC, non-muscle-invasive bladder cancer; RFS, recurrence-free survival; PFS, progression-free surviva

For peer review only

For peer review only

Table 2. Quality of the included studies assessed by NOS

Study	Selection				Comparability	Exposure			Scores
	Adequate definition of cases	Representativeness of cases	Selection of controls	Definition of controls	Control for important factor	Ascertainment of exposure	Same method to ascertain for cases and controls	Non-response rate	
Oderda[16]	—	☆	☆	☆	☆☆	☆	☆	☆	8
Park[14]	—	☆	☆	—	☆☆	☆	☆	☆	7
Quintero[28]	—	☆	☆	—	☆☆	☆	☆	—	6
Bertz[12]	—	☆	☆	—	☆☆	☆	☆	☆	7
van Rhijn[29]	—	☆	☆	—	☆☆	☆	☆	☆	7
Burger[30]	☆	☆	☆	—	☆☆	☆	☆	—	7
Queipo-zaragoza[31]	—	☆	☆	—	☆☆	☆	☆	☆	7
Lopez-Beltran[32]	—	☆	☆	☆	☆☆	☆	☆	☆	8
Santos[33]	—	☆	☆	—	☆☆	☆	☆	☆	7
Blanchet[34]	—	☆	☆	☆	☆☆	☆	☆	☆	8
Lee[35]	—	☆	☆	—	☆☆	☆	☆	☆	7

For peer review only

For peer review only

Table 3. Subgroup results of RFS and heterogeneity test

Variables	Study number	HR (95% CI)	Heterogeneity test		
			Q	*P*	I^2 (%)
Total RFS	7	1.331 (0.980–1.809)	9.48	0.148	36.7
Region					
Asian	2	0.892 (0.454–1.752)	0.32	0.570	0.0
Caucasian	5	1.441 (1.014–2.047)	7.52	0.111	46.8
Sample size					
>100	4	1.466 (0.986–2.181)	7.44	0.059	59.7
≤100	3	0.959 (0.534–1.725)	0.51	0.777	0.0
Follow-up (month)					
≥60	3	1.036 (0.758–1.415)	0.79	0.674	0.0
<60	4	1.853 (1.316–2.607)	2.62	0.453	0.0
Stage					
All NMIBC	3	1.575 (0.915–2.711)	4.44	0.109	54.9
Others	4	1.153 (0.821–1.620)	3.21	0.360	6.6
Cut off					
15%	2	1.625 (0.963–2.743)	0.31	0.575	0.0
Others	5	1.252 (0.839–1.869)	8.56	0.073	53.3
Patient age (year)					
≥70	3	1.352 (0.955–1.913)	1.16	0.559	0.0
<70	4	1.256 (0.717–2198)	8.32	0.040	63.9

NMIBC, non-muscle-invasive bladder cancer; RFS, recurrence-free survival.

For peer review only

Table 4. Subgroup results of PFS and heterogeneity test

Variables	Study number	HR (95% CI)	Heterogeneity test		
			Q	*P*	I^2 (%)
Total PFS	9	2.567 (1.562–4.219)	18.1	0.021	55.6
Region					
Asian	1	0.421 (0.084–2.114)	0.00		
Caucasian	8	2.883 (1.830–4.544)	12.99	0.072	46.1
Sample size					
>100	5	2.559 (1.372–4.774)	9.26	0.055	56.8
≤100	4	2.536 (0.943–6.818)	8.75	0.033	65.7
Follow-up (month)					
≥60	6	2.153 (0.984–4.710)	13.08	0.023	61.8
<60	3	3.158 (1.774–5.623)	3.56	0.169	43.8
Stage					
All NMIBC	3	4.673 (1.938–11.264)	3.29	0.193	39.2
Others	6	2.044 (1.213–15.040)	9.58	0.088	47.8
Cut off					
15%	1	2.800 (1.447–5.418)	0.00		
Others	8	2.515 (1.382–4.576)	17.92	0.012	60.9
Patient age (year)					
≥70	2	2.519 (1.377–4.606)	0.60	0.438	0.0
<70	7	2.654 (1.381–5.100)	17.40	0.008	65.5

NMIBC, non-muscle-invasive bladder cancer; PFS, progression-free survival.

For peer review only

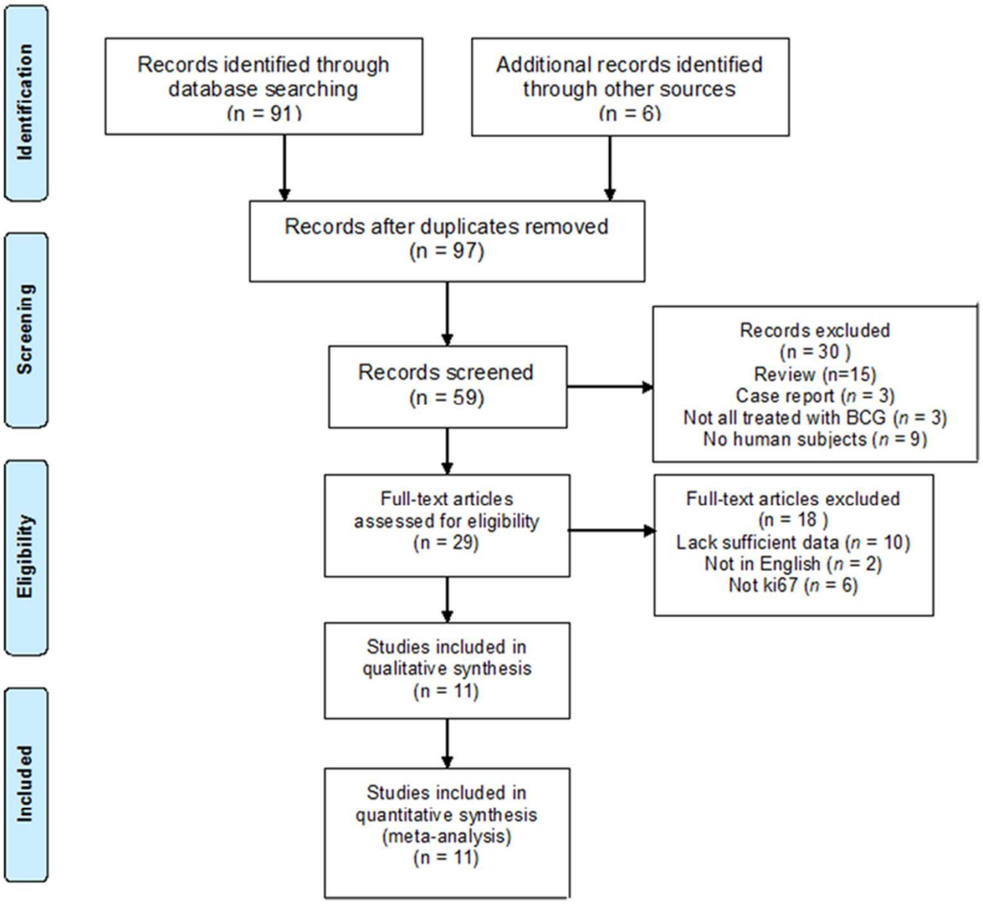

Figure 1. Flow diagram of study selection.

80x72mm (300 x 300 DPI)

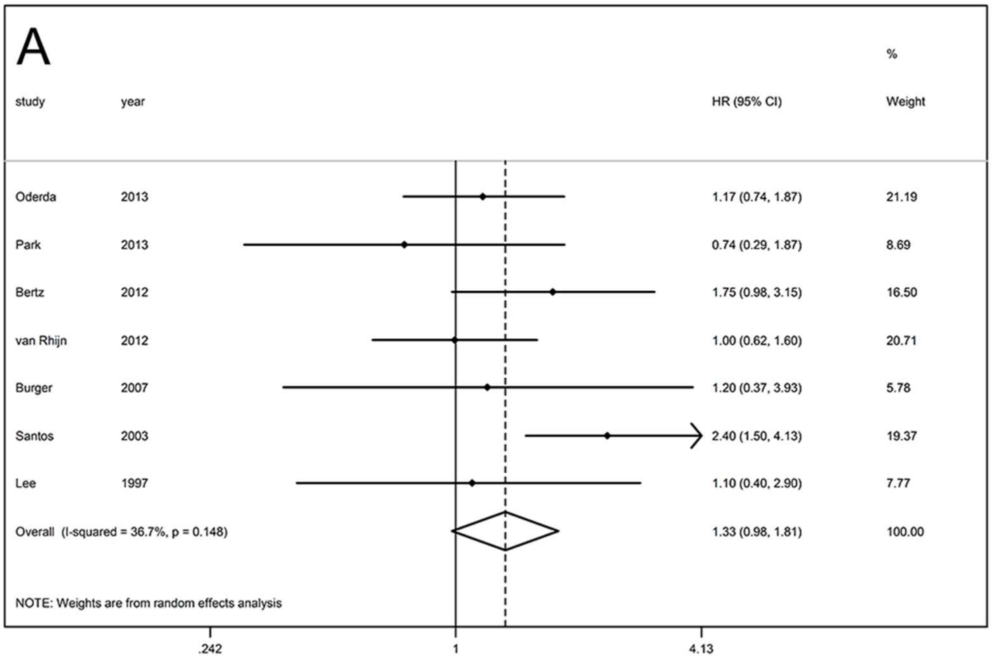

Forest plots of HRs estimated for the relationship between Ki-67 expression and RFS (A) or PFS (B) among NMIBC patients treated with BCG.

80x53mm (300 x 300 DPI)

For peer review only - http://bmjopen.bmj.com/site/about/guidelines.xhtml

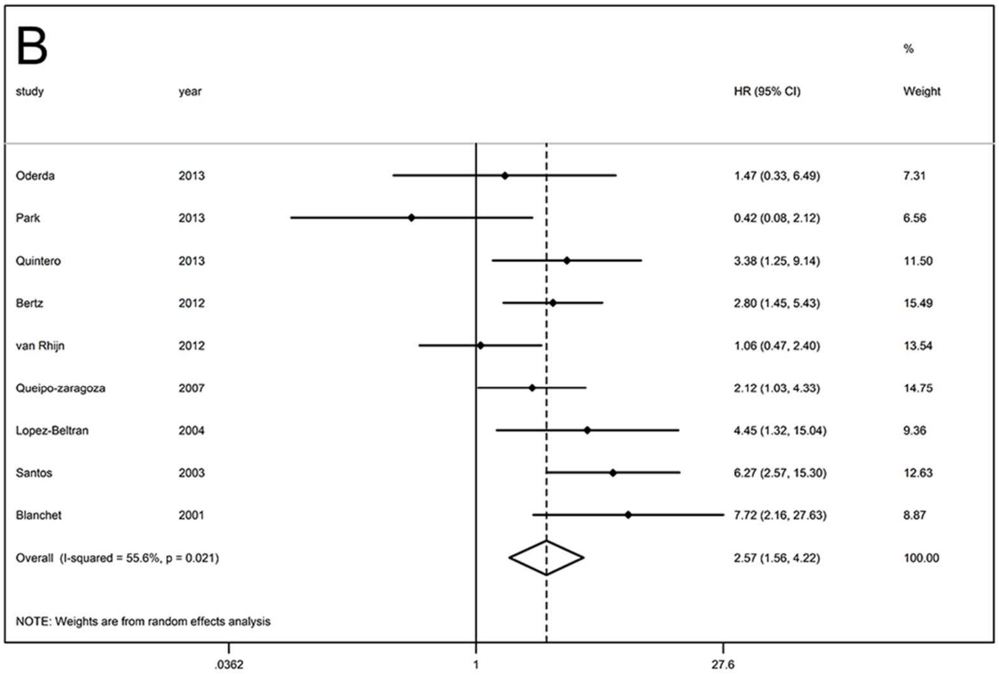

Forest plots of HRs estimated for the relationship between Ki-67 expression and RFS (A) or PFS (B) among NMIBC patients treated with BCG.

80x54mm (300 x 300 DPI)

For peer review only

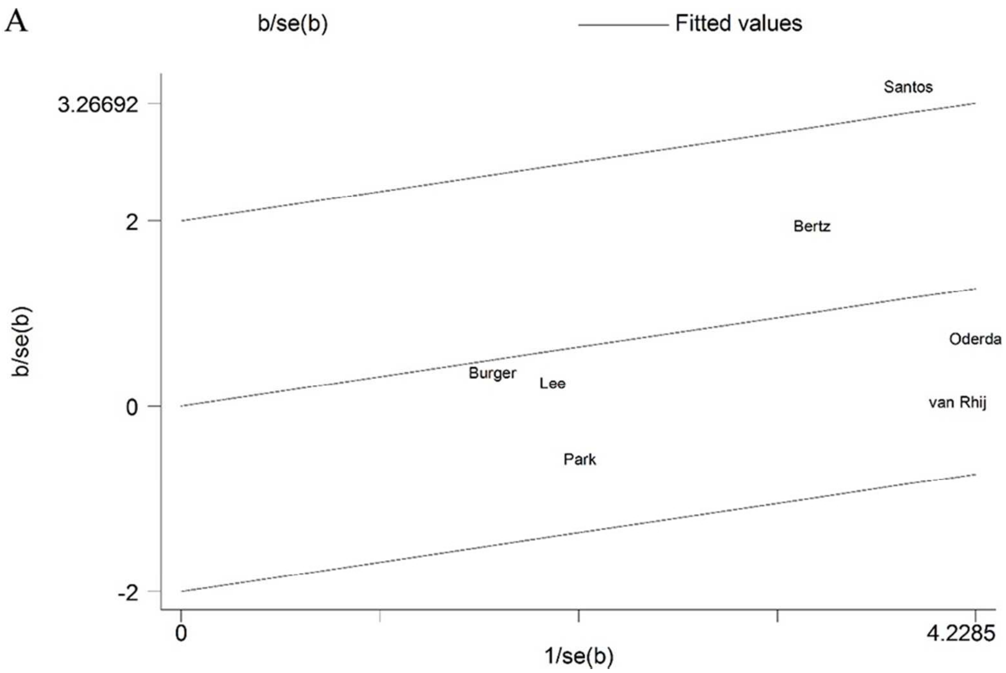

Figure 3. Galbraith plot analysis was used to evaluate heterogeneity. It suggested that two studies were the potential source of heterogeneity for RFS (A), while one was the source of heterogeneity for PFS (B)

80x52mm (300 x 300 DPI)

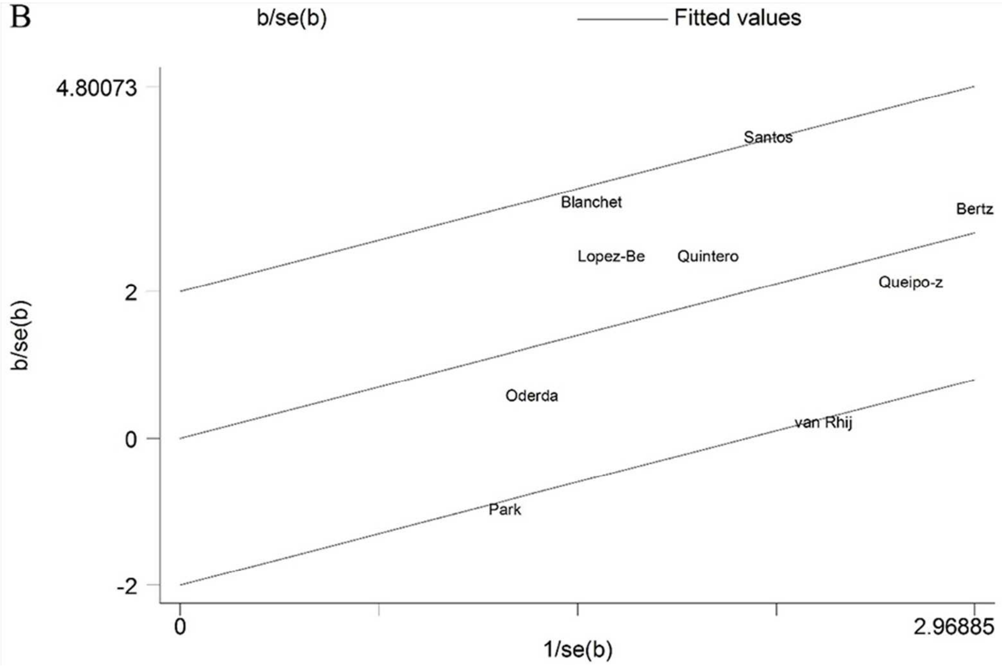

Figure 3. Galbraith plot analysis was used to evaluate heterogeneity. It suggested that two studies were the potential source of heterogeneity for RFS (A), while one was the source of heterogeneity for PFS (B)

80x53mm (300 x 300 DPI)

iew only

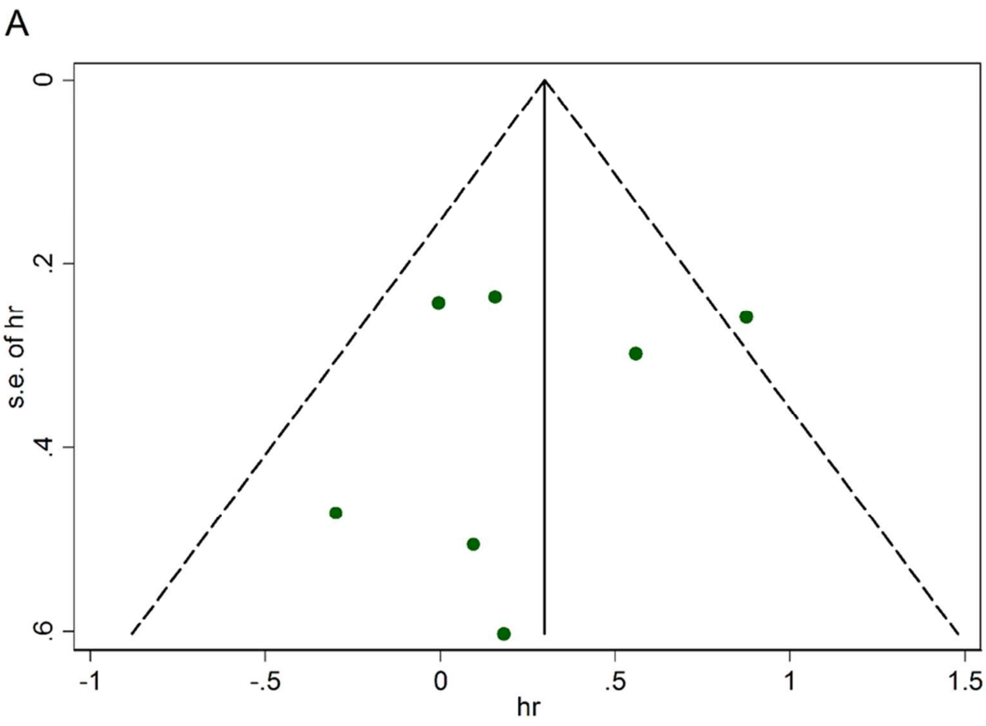

Figure 4. Funnel plots of the expression of ki67 and RFS (A) or PFS (B).

80x57mm (300 x 300 DPI)

ew only

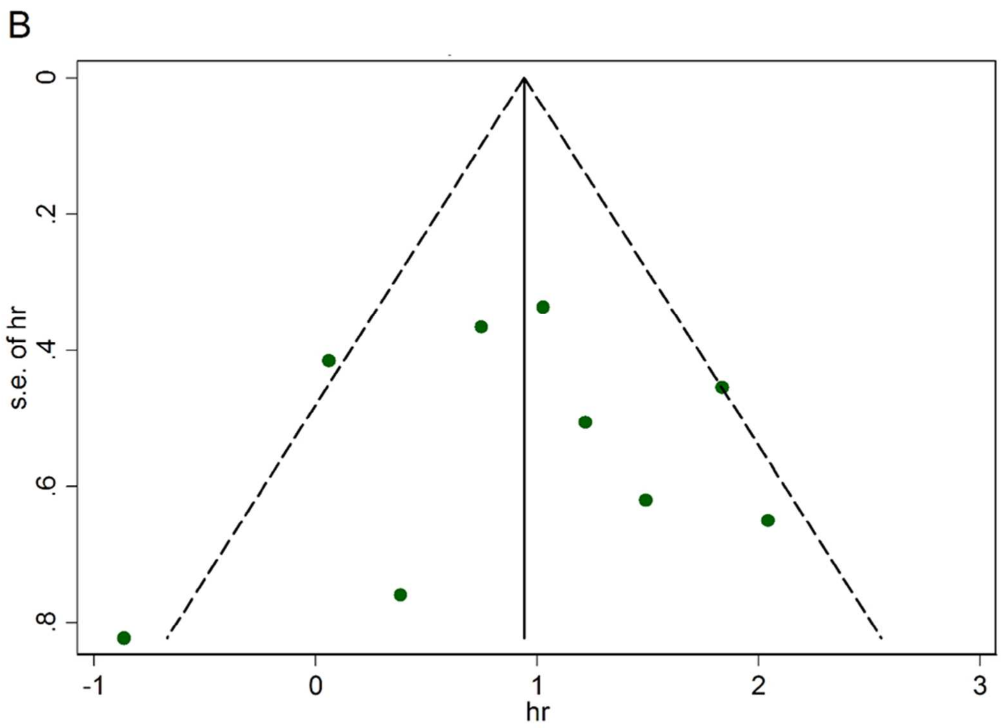

Figure 4. Funnel plots of the expression of ki67 and RFS (A) or PFS (B).

80x57mm (300 x 300 DPI)

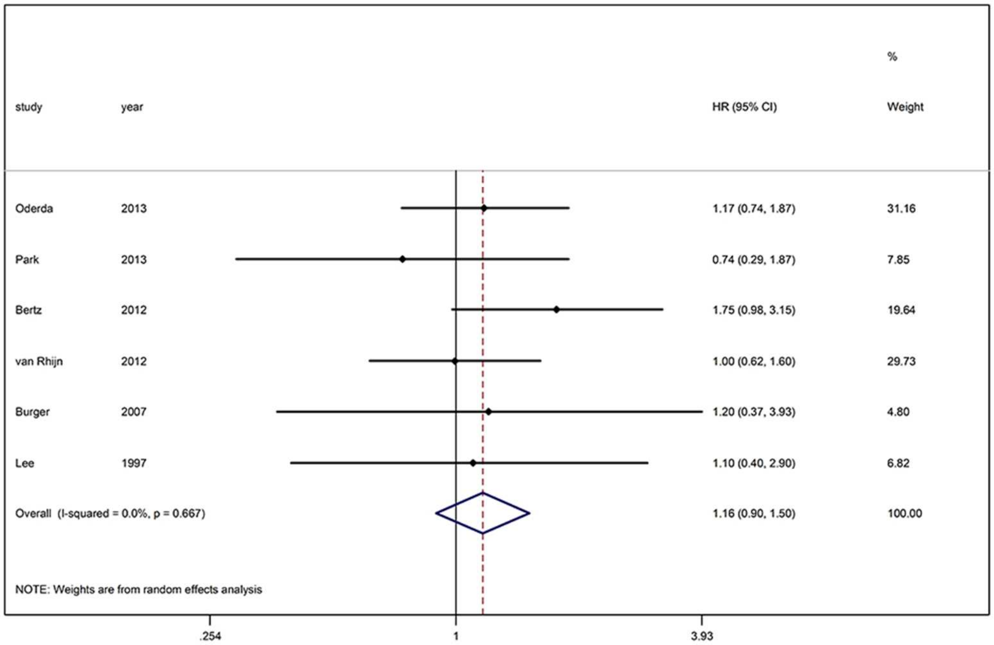

Figure S1. Forest plots of HRs estimated for the relationship between Ki-67 expression and RFS after the aforementioned study was excluded

80x52mm (300 x 300 DPI)

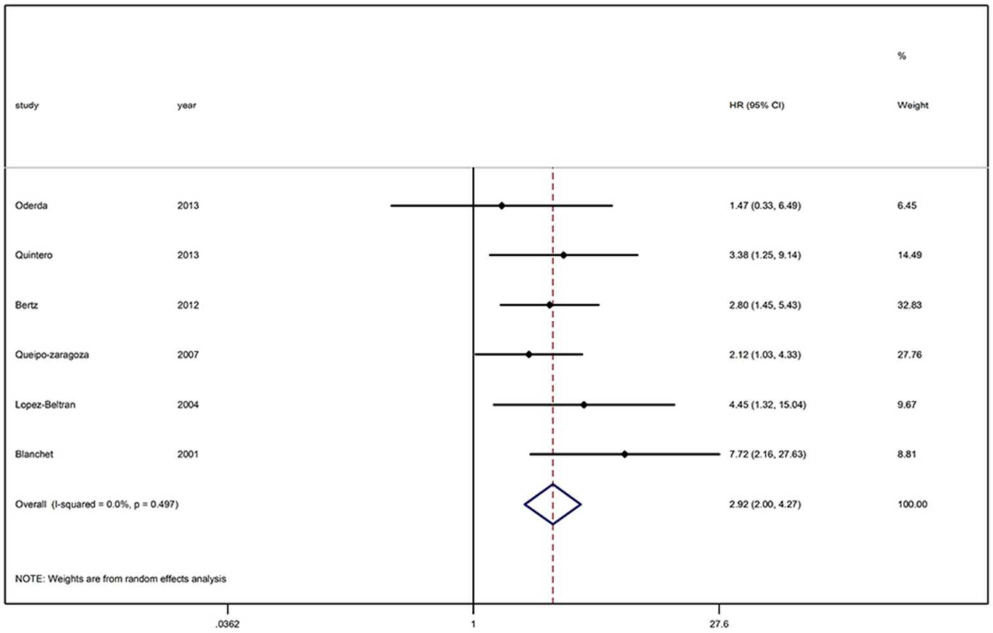

Figure S2. Forest plots of HRs estimated for the relationship between Ki-67 expression and PFS after the aforementioned study was excluded

80x51mm (300 x 300 DPI)

PRISMA 2009 Checklist

Section/topic	#	Checklist item	Reported on page #
**TITLE**			
Title	1	Identify the report as a systematic review, meta-analysis, or both.	1
**ABSTRACT**			
Structured summary	2	Provide a structured summary including, as applicable: background; objectives; data sources; study eligibility criteria, participants, and interventions; study appraisal and synthesis methods; results; limitations; conclusions and implications of key findings; systematic review registration number.	3
**INTRODUCTION**			
Rationale	3	Describe the rationale for the review in the context of what is already known.	6-8
Objectives	4	Provide an explicit statement of questions being addressed with reference to participants, interventions, comparisons, outcomes, and study design (PICOS).	6-8
**METHODS**			
Protocol and registration	5	Indicate if a review protocol exists, if and where it can be accessed (e.g., Web address), and, if available, provide registration information including registration number.	8
Eligibility criteria	6	Specify study characteristics (e.g., PICOS, length of follow-up) and report characteristics (e.g., years considered, language, publication status) used as criteria for eligibility, giving rationale.	8
Information sources	7	Describe all information sources (e.g., databases with dates of coverage, contact with study authors to identify additional studies) in the search and date last searched.	8
Search	8	Present full electronic search strategy for at least one database, including any limits used, such that it could be repeated.	8
Study selection	9	State the process for selecting studies (i.e., screening, eligibility, included in systematic review, and, if applicable, included in the meta-analysis).	8-9
Data collection process	10	Describe method of data extraction from reports (e.g., piloted forms, independently, in duplicate) and any processes for obtaining and confirming data from investigators.	8-9
Data items	11	List and define all variables for which data were sought (e.g., PICOS, funding sources) and any assumptions and simplifications made.	9
Risk of bias in individual studies	12	Describe methods used for assessing risk of bias of individual studies (including specification of whether this was done at the study or outcome level), and how this information is to be used in any data synthesis.	9
Summary measures	13	State the principal summary measures (e.g., risk ratio, difference in means).	9-10
Synthesis of results	14	Describe the methods of handling data and combining results of studies, if done, including measures of consistency (e.g., I^2) for each meta-analysis.	9-10

For peer review only - http://bmjopen.bmj.com/site/about/guidelines.xhtml

PRISMA 2009 Checklist

Section/topic	#	Checklist item	Reported on page #
Risk of bias across studies	15	Specify any assessment of risk of bias that may affect the cumulative evidence (e.g., publication bias, selective reporting within studies).	10
Additional analyses	16	Describe methods of additional analyses (e.g., sensitivity or subgroup analyses, meta-regression), if done, indicating which were pre-specified.	10
**RESULTS**			
Study selection	17	Give numbers of studies screened, assessed for eligibility, and included in the review, with reasons for exclusions at each stage, ideally with a flow diagram.	10-11
Study characteristics	18	For each study, present characteristics for which data were extracted (e.g., study size, PICOS, follow-up period) and provide the citations.	11-13
Risk of bias within studies	19	Present data on risk of bias of each study and, if available, any outcome level assessment (see item 12).	14
Results of individual studies	20	For all outcomes considered (benefits or harms), present, for each study: (a) simple summary data for each intervention group (b) effect estimates and confidence intervals, ideally with a forest plot.	15-21
Synthesis of results	21	Present results of each meta-analysis done, including confidence intervals and measures of consistency.	15-21
Risk of bias across studies	22	Present results of any assessment of risk of bias across studies (see Item 15).	22
Additional analysis	23	Give results of additional analyses, if done (e.g., sensitivity or subgroup analyses, meta-regression [see Item 16]).	22
**DISCUSSION**			
Summary of evidence	24	Summarize the main findings including the strength of evidence for each main outcome; consider their relevance to key groups (e.g., healthcare providers, users, and policy makers).	23-27
Limitations	25	Discuss limitations at study and outcome level (e.g., risk of bias), and at review-level (e.g., incomplete retrieval of identified research, reporting bias).	27
Conclusions	26	Provide a general interpretation of the results in the context of other evidence, and implications for future research.	28
**FUNDING**			
Funding	27	Describe sources of funding for the systematic review and other support (e.g., supply of data); role of funders for the systematic review.	28

From: Moher D, Liberati A, Tetzlaff J, Altman DG, The PRISMA Group (2009). Preferred Reporting Items for Systematic Reviews and Meta-Analyses: The PRISMA Statement. PLoS Med 6(6): e1000097. doi:10.1371/journal.pmed1000097

For more information, visit: www.prisma-statement.org.

For peer review only - http://bmjopen.bmj.com/site/about/guidelines.xhtml

Table S2. Meta-regression analysis of RFS and PFS

PFS

Heterogeneity factor	Coefficient	SE	t	P
Years	–0.0343	0.0283	–1.21	0.280
Country				
1	–0.3899	0.3716	–1.05	0.404
2	–0.7185	0.3503	–2.05	0.177
3	–0.9900	0.4307	–2.30	0.148
4	–0.8805	0.3541	–2.49	0.131
Numbers of patients	0.0022	0.0020	1.16	0.299
Stage	None			
Cutoff	–0.0098	0.0407	–0.24	0.819
Age	0.0021	0.0395	0.05	0.959
Follow-up	–0.0062	0.0065	–0.96	0.379

PFS

Heterogeneity factor	Coefficient	SE	t	P
Years	–0.1195	0.0461	–2.59	0.036
Country				

For peer review only

1	0.2080	0.7936	0.26	0.818
2	–0.8062	0.5662	–1.42	0.290
3	–1.4505	0.8858	–1.64	0.243
4	–2.7009	0.9407	–2.87	0.103
5	–1.7766	0.6167	–2.88	0.102
6	–0.8158	0.5281	–1.54	0.262
Numbers of patients	0.0006	0.0036	0.16	0.877
Stage				
1	–1.4505	1.5909	–0.91	0.458
2	–0.8062	1.4108	–0.57	0.625
3	0.2080	1.5332	0.14	0.904
4	–1.7766	1.4353	–1.24	0.341
5	–1.2560	1.2069	–1.04	0.407
6	–0.6170	1.4689	–0.42	0.715
Cutoff	–0.0177	0.0309	–0.57	0.585
Age	–0.0672	0.0757	–0.89	0.404
Follow-up	–0.0118	0.0159	–0.74	0.483

For peer review only

Table S3. Risk group stratification in NMIBC

**Low-risk tumors**	Primary, solitary, Ta, LG/G1, <3 cm, no CIS
**Intermediate-risk tumors**	All tumors not defined in the two adjacent categories (between the category of low and high risk)
**High-risk tumors**	Any of the following:<br>T1 tumor<br>HG/G3 tumor<br>CIS<br>Multiple, recurrent, and large (>3 cm) Ta G1G2 tumors (all conditions must be present at this point)

NMIBC, non-muscle-invasive bladder cancer; CIS, carcinoma in situ; HG, high grade; LG, low grade.

For peer review only

PRISMA 2009 Checklist

Section/topic	#	Checklist item	Reported on page #
**TITLE**			
Title	1	Identify the report as a systematic review, meta-analysis, or both.	1
**ABSTRACT**			
Structured summary	2	Provide a structured summary including, as applicable: background; objectives; data sources; study eligibility criteria, participants, and interventions; study appraisal and synthesis methods; results; limitations; conclusions and implications of key findings; systematic review registration number.	3
**INTRODUCTION**			
Rationale	3	Describe the rationale for the review in the context of what is already known.	6-8
Objectives	4	Provide an explicit statement of questions being addressed with reference to participants, interventions, comparisons, outcomes, and study design (PICOS).	6-8
**METHODS**			
Protocol and registration	5	Indicate if a review protocol exists, if and where it can be accessed (e.g., Web address), and, if available, provide registration information including registration number.	8
Eligibility criteria	6	Specify study characteristics (e.g., PICOS, length of follow-up) and report characteristics (e.g., years considered, language, publication status) used as criteria for eligibility, giving rationale.	8
Information sources	7	Describe all information sources (e.g., databases with dates of coverage, contact with study authors to identify additional studies) in the search and date last searched.	8
Search	8	Present full electronic search strategy for at least one database, including any limits used, such that it could be repeated.	8
Study selection	9	State the process for selecting studies (i.e., screening, eligibility, included in systematic review, and, if applicable, included in the meta-analysis).	8-9
Data collection process	10	Describe method of data extraction from reports (e.g., piloted forms, independently, in duplicate) and any processes for obtaining and confirming data from investigators.	8-9
Data items	11	List and define all variables for which data were sought (e.g., PICOS, funding sources) and any assumptions and simplifications made.	9
Risk of bias in individual studies	12	Describe methods used for assessing risk of bias of individual studies (including specification of whether this was done at the study or outcome level), and how this information is to be used in any data synthesis.	9
Summary measures	13	State the principal summary measures (e.g., risk ratio, difference in means).	9-10
Synthesis of results	14	Describe the methods of handling data and combining results of studies, if done, including measures of consistency (e.g., I^2) for each meta-analysis.	9-10

For peer review only

For peer review only - http://bmjopen.bmj.com/site/about/guidelines.xhtml

PRISMA 2009 Checklist

Section/topic	#	Checklist item	Reported on page #
Risk of bias across studies	15	Specify any assessment of risk of bias that may affect the cumulative evidence (e.g., publication bias, selective reporting within studies).	10
Additional analyses	16	Describe methods of additional analyses (e.g., sensitivity or subgroup analyses, meta-regression), if done, indicating which were pre-specified.	10
**RESULTS**			
Study selection	17	Give numbers of studies screened, assessed for eligibility, and included in the review, with reasons for exclusions at each stage, ideally with a flow diagram.	10-11
Study characteristics	18	For each study, present characteristics for which data were extracted (e.g., study size, PICOS, follow-up period) and provide the citations.	11-13
Risk of bias within studies	19	Present data on risk of bias of each study and, if available, any outcome level assessment (see item 12).	14
Results of individual studies	20	For all outcomes considered (benefits or harms), present, for each study: (a) simple summary data for each intervention group (b) effect estimates and confidence intervals, ideally with a forest plot.	15-21
Synthesis of results	21	Present results of each meta-analysis done, including confidence intervals and measures of consistency.	15-21
Risk of bias across studies	22	Present results of any assessment of risk of bias across studies (see Item 15).	22
Additional analysis	23	Give results of additional analyses, if done (e.g., sensitivity or subgroup analyses, meta-regression [see Item 16]).	22
**DISCUSSION**			
Summary of evidence	24	Summarize the main findings including the strength of evidence for each main outcome; consider their relevance to key groups (e.g., healthcare providers, users, and policy makers).	23-27
Limitations	25	Discuss limitations at study and outcome level (e.g., risk of bias), and at review-level (e.g., incomplete retrieval of identified research, reporting bias).	27
Conclusions	26	Provide a general interpretation of the results in the context of other evidence, and implications for future research.	28
**FUNDING**			
Funding	27	Describe sources of funding for the systematic review and other support (e.g., supply of data); role of funders for the systematic review.	28

From: Moher D, Liberati A, Tetzlaff J, Altman DG, The PRISMA Group (2009). Preferred Reporting Items for Systematic Reviews and Meta-Analyses: The PRISMA Statement. PLoS Med 6(6): e1000097. doi:10.1371/journal.pmed1000097

For more information, visit: **www.prisma-statement.org**.

For peer review only - http://bmjopen.bmj.com/site/about/guidelines.xhtml

BMJ Open

Prognostic Value of ki67 in Bacillus Calmette–Guérin-Treated Non-muscle-Invasive Bladder Cancer: a Meta-analysis and Systematic Review

Journal:	*BMJ Open*
Manuscript ID	bmjopen-2017-019635.R1
Article Type:	Research
Date Submitted by the Author:	13-Jan-2018
Complete List of Authors:	He, Yuhui; Peking University China-Japan Friendship School of Clinical Medicine; China-Japan Friendship Hospital, Department of Urology<br>Wang, Ning; North China University of Science and Technology<br>Zhou, Xiaofeng; Peking University China-Japan Friendship School of Clinical Medicine; China-Japan Friendship Hospital, Department of Urology<br>Wang, Jianfeng; China-Japan Friendship Hospital, Department of Urology<br>Ding, Zhenshan; China-Japan Friendship Hospital, Department of Urology<br>Chen, Xing ; China-Japan Friendship Hospital, Department of Urology<br>Deng, Yisen; Peking University China-Japan Friendship School of Clinical Medicine; China-Japan Friendship Hospital, Department of Urology
**Primary Subject Heading**:	Urology
Secondary Subject Heading:	Urology
Keywords:	ki67, meta-analysis, non-muscular-invasive bladder cancer, prognosis

SCHOLARONE™
Manuscripts

Prognostic Value of ki67 in Bacillus Calmette–Guérin-Treated Non-muscle-Invasive Bladder Cancer: a Meta-analysis and Systematic Review

Yuhui He[1, 2], Ning Wang[3], Xiaofeng Zhou[1, 2*], Jianfeng Wang[2], Zhenshan Ding[2], Xing Chen[2], Yisen Deng[1, 2]

[1] Peking University China-Japan Friendship School of Clinical Medicine, Beijing 100029, China

[2] Department of Urology, China-Japan Friendship Hospital, Beijing 100029, China

[3] North China University of Science and Technology, Tangshan 063013, China

***Corresponding Author**

Xiaofeng Zhou

Peking University China-Japan Friendship School of Clinical Medicine, Beijing 100029, China

Department of Urology, China-Japan Friendship Hospital, Beijing 100029, China

Tel: +86 13121391766

Email: doctorzxf@126.com

Fax: +86 64217749

Word count

Title page: 98

Abstract: 251

References: 1403

For peer review only - http://bmjopen.bmj.com/site/about/guidelines.xhtml

Number of tables, figures, supplementary files: 4, 4, 5

For peer review only - http://bmjopen.bmj.com/site/about/guidelines.xhtml

Abstract

Objectives: The aim of this study was to explore the prognostic value of ki67 as a marker in non-muscle-invasive bladder cancer (NMIBC) patients treated with Bacillus Calmette–Guérin (BCG).

Methods: Studies were systematically retrieved from the relevant databases (Web of Science, PubMed, Cochrane Library, and Embase), and the expiry date was May 2017. The research steps referred to the Preferred Reporting Items for Systematic Reviews and Meta-Analysis statement.

Results: A total of 11 studies that complied with the inclusion criteria were enrolled. The expression of ki67 was not statistically significantly correlated with recurrence-free survival (RFS) [hazard ratio (HR): 1.331; 95% CI: 0.980–1.809)]. No significant heterogeneity was found among all included studies (I^2 = 36.7%, P = 0.148). The expression of ki67 was statistically significantly correlated with progression-free survival (PFS) (HR: 2.567; 95% CI: 1.562–4.219), and the overexpression of ki67 was the risk factor for PFS. Significant heterogeneity was noted among all the included studies (I^2 = 55.6%, P = 0.021). The studies that might cause heterogeneity were excluded using the Galbraith plot, and then the meta-analysis was performed again. The results showed that the expression of ki67 was still correlated with PFS (HR: 2.922; 95% CI: 2.002–4.266).

Conclusions: The overexpression of ki67 was the risk factor for PFS, and the relationship between the expression of ki67 and RFS was not statistically significant in patients with NMIBC treated with BCG intravesical immunotherapy. Well-designed, prospective, randomized controlled trials with a large sample size are still needed to validate these findings.

Key words: ki67; meta-analysis; non-muscular-invasive bladder cancer; prognosis

For peer review only - http://bmjopen.bmj.com/site/about/guidelines.xhtml

Strengths and limitations of this study

This meta-analysis and systematic review was performed via a strict literature search. It was the first meta-analysis to evaluate the prognostic value of ki67 in patients with NMIBC after transurethral resection and BCG intravesical immunotherapy.

The number of studies considered in the final meta-analysis was 11. This small sample size limited the potential analyses. The research did not consider the surgical skills mentioned in published studies.

Despite a systematic search strategy, the inclusion criteria excluded non-English documents and had language bias. The meta-regression analysis suggested no bias, but a selection bias was likely.

These limitations notwithstanding, the research can guide the follow-up research on immunohistochemical markers and clinical practice in non-muscular-invasive bladder cancer.

Introduction

Bladder cancer is one of the most common clinical urological tumors. It is a direct threat to the survival of patients with the disease. The incidence of bladder cancer varies across the world, with the highest rate in the developed communities.[1] A total of 429,800 new cases of bladder cancer and 165,100 deaths occurred in 2012 worldwide. Bladder cancer occurs mostly in men, and about a tenfold variation in incidence rates has in been reported internationally.[2] About 70% of these patients have non-muscle-invasive bladder cancer (NMIBC).[3] Four major organizational guidelines on NMIBC, including the American Urological Association/Society of Urologic Oncology, the European Association of Urology, the National Comprehensive Cancer

For peer review only - http://bmjopen.bmj.com/site/about/guidelines.xhtml

Network, and the National Institute for Health and Care Excellence guidelines, recommend that the proper initial transurethral resection (TUR) of bladder tumor is a critical step in the initial management and staging of the disease.[4] However, TUR surgery alone cannot solve the postoperative problems for NMIBC because of high recurrence rate and disease development.[5] Postoperative TUR associated with Bacillus Calmette–Guérin (BCG) intravesical immunotherapy can prevent the postoperative recurrence of NMIBC and significantly reduce the moderate and high development risk of NMIBC.[6, 7] However, the postoperative BCG intravesical immunotherapy still has some problems. The failure rate of BCG intravesical therapy in NMIBC is about 40%–50%.[8] Furthermore, BCG also has toxic side effects, such as hepatitis, pneumonitis, epididymitis/orchitis, abscess formation, bladder contracture, ureteral obstruction, BCG sepsis, leukopenia, and hematuria.[9] Therefore, BCG therapy should be individually performed, and the patients in whom BCG therapy is ineffective should be timely recognized. These patients or those with poor prognosis should receive radical cystectomy or any other therapy in time to avoid futile treatment and alleviate pain. However, the recognition of patients with no effect of TUR postoperative BCG intravesical immunotherapy is still hard due to the heterogeneity of bladder cancer and individuality of patients.[10] Therefore, finding the prognostic factors for patients with NMIBC receiving TUR and BCG therapy is extremely necessary.

The recurrence rate of bladder cancer treated with different therapies is between 50% and 80%, and about 15% of the low-grade tumor recurrence involves high-grade tumors.[11] The patients need periodical cystoscopy to find the recurrent focus in time. A reliable prognostic molecular marker can reduce the pain caused by cystoscopy. Because NMIBC does not have reliable prognostic markers, it is hard to decide

For peer review only - http://bmjopen.bmj.com/site/about/guidelines.xhtml

postoperative therapy in the clinic,[12] which depends mainly on the clinical guidelines and physician's experience. Currently, some of the published studies about immunohistochemical markers have evaluated the prognostic value of BCG intravesical immunotherapy on the patients first receiving TUR. The main immunohistochemical markers include ki67, p53, p27, pRb, CD9, CD20, E2F1, and so forth.[13, 14] However, no immunohistochemical marker has been confirmed so far. The prognostic value of ki67 antigen on the survival in patients with NMIBC receiving BCG intravesical immunotherapy has been controversial. For example, Kruger.[15] reported that ki67 antigen was an independent predictive factor for the recurrence of pT1 stage tumor, but Oderde[16] believed that ki67 was an independent predictive factor for the recurrence of all NMIBCs. Zlotta[17] reported that ki67 antigen had no independent prognostic value in patients receiving BCG therapy. Saint[18] retrospected the recent 25-year published studies and believed that the independent prognostic factor for bladder cancer in patients receiving BCG therapy was not clear. An international consensus group listed various bladder cancer prognostic indexes by reviewing PubMed and considered that although some markers (such as ki67 and p53) could predict the recurrence and development of bladder cancer, the data still had heterogeneity. Thus, strict test criteria and clear statistical methods should be established for further evaluation.[19]

A meta-analysis can enlarge the sample size by integrating independent studies with small sample size, further increase the statistical efficacy, and reduce the wrong conclusion caused by the small sample size.[20] The aim of this study was to explore the prognostic value of ki67 as a marker in patients with NMIBC treated with BCG. Based on the literature search, this study was the first meta-analysis to evaluate the prognostic value of ki67 in patients with NMIBC treated with BCG.

For peer review only - http://bmjopen.bmj.com/site/about/guidelines.xhtml

Methods

This meta-analysis was performed according to the Preferred Reporting Items for Systematic Reviews and Meta-Analyses (PRISMA) statement (**Table S1**).[21] The present meta-analysis did not need the approval because all the enrolled published studies were approved by the ethics committee in there research institute.

Literature retrieval strategy

The comprehensive literature search was performed on Web of Science, PubMed, Cochrane Library, and Embase databases for relevant studies. The last quest was updated on May 24, 2017, with hand-searching to identify any potentially eligible studies that might have been missed. The following search strategy was adopted for each database: ("Urinary Bladder Neoplasms"[Mesh] OR "bladder cancer" OR "bladder carcinoma" OR " bladder tumor ") AND ("BCG Vaccine"[Mesh] OR "BCG" OR "Bacillus Calmette–Guérin") AND ("ki67 antigen "[Mesh] OR " ki-67" OR " ki67" OR " MBI-1"). Filters were as follows: retrospective, array research, clinical trial, controlled clinical trial, and randomized controlled trial. Free word retrieval strategy was used. The enrolled contents included the reference lists and relevant suggestive references while searching.

Inclusion and exclusion criteria

The inclusion criteria were as follows: (1) prospective or retrospective published studies evaluating the prognostic relationship between the expression of ki67 and NMIBC treated with BCG; (2) the expression of ki67 in tissues detected by immunohistochemistry analysis; (3) hazard ratio (HR) and 95% confidence interval (95% CI) directly obtained from the published studies; and (4) published English studies. The exclusion criteria were as follows: (1) review, systematic evaluation, case

For peer review only - http://bmjopen.bmj.com/site/about/guidelines.xhtml

report, editorial, and specialist experience; (2) studies with no human subjects; and (3) published studies in which data could not be extracted or those having wrong data.

Data extraction and evaluation of literature quality

Based on the aforementioned criteria, two reviewers independently screened the published studies by reading titles and abstracts and got preliminary conclusions. If the conclusions were not consistent, the literature was discussed by all the authors to decide its enrollment. The relevant information of the enrolled published studies was extracted, such as first author, publication time, research country, sex, case number, age, follow-up date, disease stage, cutoff values, recurrence-free survival (RFS), and progression-free survival (PFS). The Newcastle–Ottawa Scale (NOS) was used to evaluate the quality of all the published studies.[22] Scores 0–3, 4–5, and 6–8 were accepted as low, medium, and high quality, respectively.

Statistical methods

The measuring time and method of ki67 complied with the standard of clinical routine and pathological examination. Tumor tissue samples were taken in accordance with the standard surgical procedure and used for immunohistochemical analysis. RFS and PFS were the traditionally used statistical parameters. PFS was defined as the time from the beginning of treatment to the first progression. RFS was defined as the time from the removal of the lesion (or the randomization of the clinical trial) until the recurrence or death of the tumor. The impact of the expression of ki67 on survival was quantified using the combined HRs and 95% CIs. The HR and 95% CI of each study were directly extracted from each original published study. Besides, the Parmar and Tierney's[23] method was used to extract the data because some of the published studies did not directly provide HR and 95% CI. For example, some studies provided only the survival curve. In this meta-analysis, the DerSimonian–Laird random-effects model[24]

For peer review only - http://bmjopen.bmj.com/site/about/guidelines.xhtml

was used because when the heterogeneity was large, only the random-effects model could be suitably used. Similar to traditional methods, HR >1 was considered as the prognostic risk factor for the overexpression of ki67, and HR <1 was a protective factor. 95% CI <1 indicated a statistically significant difference in the relationship between the overexpression of ki67 and prognosis.

The heterogeneity was calculated according to chi-square-based Q test and I^2 statistic.[25] The heterogeneity was judged using the I^2 value (low heterogeneity: I^2 <25%; moderate heterogeneity: I^2 = 25%–50%; large heterogeneity: I^2 >50%). Besides, A P value >0.05 was considered as low heterogeneity. Then, the subgroup analysis based on regions, sample size, follow-up period, tumor grading, cutoff value, publication time, and patient age was performed. A value of 1% was considered to be a statistically significant level in the subgroup analysis. A Galbraith plot was used to search published studies with heterogeneity[26], and the meta-analysis was performed again after excluding these published studies. Meanwhile, the factors causing heterogeneity were also explored using the residual maximum likelihood (REML)-based random-effects meta-regression analysis.[27] All the statistical analyses were performed using the Stata12.0 software (StataCorp, TX, USA), and the two-sided test was used to evaluate the P value.

Evaluation of publication bias

Begg's plot and Egger's test method were used to find the possible publication bias. A P value <0.05 was considered to indicate publication bias.

Results

Literature screening

A total of 97 published studies were retrieved. Furthermore, 68 of them were excluded

For peer review only - http://bmjopen.bmj.com/site/about/guidelines.xhtml

after duplicates were removed and records screened, and 18 were excluded after reading the full text (10 published studies from which HR and 95% CI could not be obtained, 2 non-English studies, and 6 that did not use ki67 detection). Finally, 11 published studies were enrolled in the meta-analysis (**Figure 1**).

Basic characteristics and quality evaluation of enrolled published studies

The enrolled 11 published studies were published between 1997 and 2013, and the countries included Italy, South Korea, Spain, Germany, New Zealand, Canada, Portugal, and France. The largest sample size was 309, and the smallest one was 32. A total of 1321 patients were enrolled in this study. The follow-up period was beyond 36 months, and the longest was 229 months. T1 was the main tumor grading, and the cutoff value ranged from 10.4% to 40%. Seven published studies reported patients' RFS, and nine reported PFS (**Table 1**). One literature was scored as 6 star by NOS, seven as 7 star, and three as 8 star. The median of the NOS score was 7 (**Table 2**).

For peer review only

Table 1. Main characteristics of all studies included in this meta-analysis

Study	Year	Country	Male/Female	No. of patients	Age (year)	Follow-up (month)	Stage	Cutoff	Survival analysis
Oderda[16]	2013	Italy	166/26	192	73.2 (SD 11.9)	100 (2–229)	All NMIBC	20%	RFS
Park[14]	2013	Korea	53/8	61	66 (31–85)	60 (6–217)	T1G3	10.4%	RFS/PFS
Quintero[28]	2013	Spain	143/21	164	61 (29–93)	75 (60–144)	Ta	13%	PFS
Bertz[12]	2012	Germany	237/72	309	71.7 (38–87)	49 (5–172)	pT1	15%	RFS/PFS
van Rhijn[29]	2012	Netherlands, Canada	105/24	129	68.8 (SD 9.9)	78 (39.6-110.4)	T1	25%	RFS/PFS
Burger[30]	2007	Germany	45/21	71	71 (52–94)	39 (1–133)	T1/Ta	15%	RFS
Queipo-zaragoza[31]	2007	Spain	71/12	83	68.1 (SD 8.5)	All >36	T1G3	40%	PFS
Lopez-Beltran[32]	2004	Spain	49/2	51	69.96 (49–89)	63.82 (60–144)	T1G3	13%	PFS
Santos[33]	2003	Portugal	115/44	159	66 (21–88)	46.5 (4–123)	pTa/pT1	18%	RFS/PFS
Blanchet[34]	2001	France	-	70	62.6 (21–84)	64 (12–111)	pT1/pTa	13%	PFS
Lee[35]	1997	Korea	28/4	32	57.1 (30–81)	All >24	T1G2-3	20%	RFS

NMIBC, Non-muscle-invasive bladder cancer; no., number; PFS, progression-free survival; RFS, recurrence-free survival.

For peer review only - http://bmjopen.bmj.com/site/about/guidelines.xhtml

Table 2. Quality of the included studies assessed by NOS

Study	Selection				Comparability	Exposure			Scores
	Adequate definition of cases	Representativeness of cases	Selection of controls	Definition of controls	Control for important factor	Ascertainment of exposure	Same method to ascertain for cases and controls	Non-response rate	
Oderda[16]	—	☆	☆	☆	☆☆	☆	☆	☆	8
Park[14]	—	☆	☆	—	☆☆	☆	☆	☆	7
Quintero[28]	—	☆	☆	—	☆☆	☆	☆	—	6
Bertz[12]	—	☆	☆	—	☆☆	☆	☆	☆	7
van Rhijn[29]	—	☆	☆	—	☆☆	☆	☆	☆	7
Burger[30]	☆	☆	☆	—	☆☆	☆	☆	—	7
Queipo-zaragoza[31]	—	☆	☆	—	☆☆	☆	☆	☆	7
Lopez-Beltran[32]	—	☆	☆	☆	☆☆	☆	☆	☆	8
Santos[33]	—	☆	☆	—	☆☆	☆	☆	☆	7
Blanchet[34]	—	☆	☆	☆	☆☆	☆	☆	☆	8
Lee[35]	—	☆	☆	—	☆☆	☆	☆	☆	7

For peer review only - http://bmjopen.bmj.com/site/about/guidelines.xhtml

For peer review only

Influence of the expression of ki67 on RFS

Seven published studies reported the expression of ki67 and PFS results of patients with NMIBC treated with BCG. The meta-analysis indicated that ki67 had no statistically significant association with RFS (HR: 1.331; 95% CI: 0.980–1.809), and no heterogeneity among the enrolled studies was reported (I^2 = 36.7%, P = 0.148) (**Figure 2A**). The subgroup analysis was performed based on the regions, sample size, follow-up period, stage, cutoff value, publication time, and age. Meanwhile, all the original published studies on the correlation between ki67 expression and RFS in patients with NMIBC treated with BCG were multivariate, and the HRs were adjusted. The stratification analysis by region indicated that ki67 was also significantly associated with RFS in Caucasians and a follow-up period shorter than 6 months. (HR: 1.441, 95% CI: 1.014–2.047; HR: 1.853, 95% CI: 1.316–2.607) (**Table 3**).

For peer review only - http://bmjopen.bmj.com/site/about/guidelines.xhtml

Table 3. Subgroup results of RFS and heterogeneity test

Variables	Study number	HR (95% CI)	Heterogeneity test		
			Q	*P*	I^2 (%)
Total RFS	7	1.331 (0.980–1.809)	9.48	0.148	36.7
Region					
Asian	2	0.892 (0.454–1.752)	0.32	0.570	0.0
Caucasian	5	1.441 (1.014–2.047)	7.52	0.111	46.8
Sample size					
>100	4	1.466 (0.986–2.181)	7.44	0.059	59.7
≤100	3	0.959 (0.534–1.725)	0.51	0.777	0.0
Follow-up (month)					
≥60	3	1.036 (0.758–1.415)	0.79	0.674	0.0
<60	4	1.853 (1.316–2.607)	2.62	0.453	0.0
Stage					
All NMIBC	3	1.575 (0.915–2.711)	4.44	0.109	54.9
Others	4	1.153 (0.821–1.620)	3.21	0.360	6.6
Cutoff					
15%	2	1.625 (0.963–2.743)	0.31	0.575	0.0
Others	5	1.252 (0.839–1.869)	8.56	0.073	53.3
Publication year					
≥2012	4	1.164 (0.874,1.550)	3.20	0.362	6.3
<2012	3	1.774 (1.046,3.008)	2.57	0.277	22.1
Patient age (year)					
≥70	3	1.352 (0.955–1.913)	1.16	0.559	0.0
<70	4	1.256 (0.717–2198)	8.32	0.040	63.9

NMIBC, Non-muscle-invasive bladder cancer; RFS, recurrence-free survival.

For peer review only

For peer review only - http://bmjopen.bmj.com/site/about/guidelines.xhtml

Influence of the expression of ki67 on PFS

A total of nine published studies reported the expression of ki67 and PFS results of patients in NMIBC treated with BCG. The meta-analysis indicated that ki67 had no statistically significant association with RFS (HR:2.567, 95% CI: 1.562–4.219), and the overexpression of ki67 was the risk factor for PFS. Statistically significant heterogeneity was found among all the included studies (I^2 = 55.6%, P = 0.021) (**Figure 2B**). The subgroup analysis was performed based on the regions, sample size, follow-up period, stage, cutoff value, publication time, and age. However, the data extracted from six original published studies on the correlation between the expression of p53 and PFS in patients with NMIBC treated with BCG were multivariate with adjusted HRs, whereas three original published studies were univariate with unadjusted HRs. In the stratified analyses by the region, sample size, follow-up time, stage, cutoff, publication year, and patient age, significant associations were observed in the studies with Caucasian subgroup, sample size >100, follow-up period <6 months, other cutoffs, and two subgroups based on age (HR: 1.97, 95% CI: 1.04–3.74; HR: 2.37, 95% CI: 1.23–4.55; HR:2.49, 95% CI: 1.19–5.21; HR: 2.515, 95% CI: 1.382–4.576; HR: 2.800, 95% CI: 1.447–5.418; and HR: 2.654, 95% CI: 1.381–5.100, respectively). However, significant associations were also observed in both multivariate and univariate analyses (HR: 2.10, 95% CI: 1.07–1.12; HR: 2.80, 95%CI: 1.65–7.85, respectively), and the effect size suggested the same outcomes (HR: 2.567, 95% CI: 1.562–4.219) (**Table 4**).

For peer review only - http://bmjopen.bmj.com/site/about/guidelines.xhtml

For peer review only

Table 4. Subgroup results of PFS and heterogeneity test

Variables	Study number	HR (95% CI)	Heterogeneity test		
			Q	*P*	I^2 (%)
Total PFS	9	2.567 (1.562–4.219)	18.1	0.021	55.6
Region					
Asian	1	0.421 (0.084–2.114)	0.00		
Caucasian	8	2.883 (1.830–4.544)	12.99	0.072	46.1
Sample size					
>100	5	2.559 (1.372–4.774)	9.26	0.055	56.8
≤100	4	2.536 (0.943–6.818)	8.75	0.033	65.7
Follow-up (month)					
≥60	6	2.153 (0.984–4.710)	13.08	0.023	61.8
<60	3	3.158 (1.774–5.623)	3.56	0.169	43.8
Stage					
All NMIBC	3	4.673 (1.938–11.264)	3.29	0.193	39.2
Others	6	2.044 (1.213–15.040)	9.58	0.088	47.8
Cut off					
15%	1	2.800 (1.447–5.418)	0.00		
Others	8	2.515 (1.382–4.576)	17.92	0.012	60.9
Publication year					
≥2012	5	1.685（0.883,3.215）	8.04	0.090	50.2
<2012	4	4.176（2.209,7.884）	5.00	0.172	40.0
Patient age (year)					
≥70	2	2.519 (1.377–4.606)	0.60	0.438	0.0
<70	7	2.654 (1.381–5.100)	17.40	0.008	65.5
Multivariate/Univariate					
Multivariate	6	2.101 (1.070–1.121)	13.83	0.031	63.8
Univariate	3	2.803 (1.652–7.856)	3.38	0.001	40.8

For peer review only

For peer review only - http://bmjopen.bmj.com/site/about/guidelines.xhtml

NMIBC, Non-muscle-invasive bladder cancer; PFS, progression-free survival.

For peer review only

For peer review only - http://bmjopen.bmj.com/site/about/guidelines.xhtml

Galbraith plot

Using Galbraith plot (**Figure 3A**), it was found that Santos[33] was the main reason for the heterogeneity of RFS. After the aforementioned study was excluded, the remaining RFS studies had no significant heterogeneity according to the new meta-analysis (I^2 = 0.0%, P = 0.667). However, the expression of ki67 still had no statistically significant association with RFS (HR: 1.161, 95% CI: 0.896–1.504) (**Figure S1**). Using the Galbraith plot (**Figure 3B**), it was found that Santos,[33] Park,[14] and van Rhijn[29] were the main reason for the heterogeneity of PFS. After these studies were excluded, the remaining RFS studies had no significant heterogeneity according to the new meta-analysis (I^2 = 0.0%, P = 0.497). The expression of ki67 still had statistically significant association with PFS (HR: 2.922, 95% CI: 2.002–4.266) (**Figure S2**).

Meta-regression analysis

The meta-regression analysis indicated that the factors influencing heterogeneity (publication time, research region, sample size, stage, cutoff value, age, and follow-up period) might not be the reason for RFS heterogeneity. Publication time was the reason for heterogeneity of PFS (P = 0.036), but other factors were not (**Table S2**).

Publication bias

The funnel plot (**Figure 4A**) was basically symmetrical for RFS. The results of Begg's test and Egger's test showed P (Begg's) = 0.761, P (Egger's) = 0.601. The funnel plot (**Figure 4B**) was also basically symmetrical for PFS. The results of Begg's test and Egger's test showed P (Begg's) = 0.917, P (Egger's) = 0.964.

Discussion

A total of 11 published studies with 1321 cases complying with the inclusion criteria

For peer review only - http://bmjopen.bmj.com/site/about/guidelines.xhtml

were enrolled in this meta-analysis. The results of the meta-analysis indicated that the expression of ki67 had no statistically significant association with RFS, but it was significantly correlated with PFS. The overexpression of ki67 was the risk factor for PFS. It suggested that ki67 was the prognostic predictive marker in patients with NMIBC treated with BCG. Besides, the aforementioned conditions did not change after excluding the published studies possibly causing heterogeneity and reperforming the meta-analysis. It further proved that the result of the aforementioned meta-analysis was stable, that is, the overexpression of ki67 was the risk factor for PFS. In the Caucasian subgroup for PFS, racial classification and regional factors might be crucial in the prognosis of patients with NMIBC after BCG therapy. This may be related to the existence of different drug gene susceptibilities in people of different races and living areas. The two subgroups based on age in PFS, suggesting that age might be the important factor influencing the prognosis of bladder cancer. This also comply to our clinical praticse. The elder the patient, the worse the prognosis. There are several sources of heterogeneity in the above-mentioned subgroup analysis: (1) Due to the influence of race and environment, the documents included in this article come from different regions and countries. There are a large number of studies that confirm the differences in disease susceptibility between ethnic groups and regions. (2) Because of different regions and different clinicians, in the TUR and BGC perfusion treatment, there are differences in the operation of health care workers. Such as surgical clearance of the tumor. The tumor with a broad base surface is often not easy to remove completely, which also depends on the surgeon's experience and surgical skills. And the quality of BCG manufacturers may vary from region to region. (3) Different researchers' literature may include the bias of research object, research design, measuring instrument and so on. However, in general, heterogeneity does not

For peer review only - http://bmjopen.bmj.com/site/about/guidelines.xhtml

affect the conclusion. Besides, the meta-regression analysis indicated publication time as the reason for PFS heterogeneity. We consider it is relate to the improvement of testing technology, research level as well as the quality and quantity of published articles, which will be helpful for the follow-up researches. As all the original data extracted from published studies on the correlation between the expression of ki67 and RFS in patients with NMIBC treated with BCG were multivariate, the result was considered to be precise because the HRs were adjusted, excluding the confounding factors such as age and gender. However, the original data extracted from published studies on the correlation between ki67 expression and PFS were both multivariate and univariate. It was believed that the aforementioned adjustments did not have a significant impact on meta-analyses. Besides, according to the funnel plot, Begg's test and Egger's test, the enrolled studies had no statiscally significant publication bias. Thus, the reliability of the present meta-analysis was high.

In 2016, the European Association of Urology (EUA)[36] recommended a scoring system for the prognostic evaluation of NMIBC based on six clinical and pathological factors proposed by the European Organization for the Research and Treatment of Cancer-Genito-Urinary Cancer Group (EORTC), including number of tumors, tumor size, prior recurrence rate, T category, presence of concurrent carcinoma in situ, and tumor grade (**Table S3**). The tumors were categorized into low-risk tumors, intermediate-risk tumors, and high-risk tumors using this assessment system to evaluate the prognosis. For the patients after BCG therapy, the EUA recommended another risk calculator developed by the Club Urologico Espanol de Tratamiento Oncologico (CUETO) and the EORTC. This calculator was based on gender, age, recurrent tumor, number of tumors, T category, associated Tis, and grade. The CUETO risk calculator can be achieved at http://www.aeu.es/Cueto.html. The

For peer review only - http://bmjopen.bmj.com/site/about/guidelines.xhtml

recommended level was B grade for the two scales for patients with NMIBC, whether used alone or combined. The two scales could be used together in the clinic. When using the CUETO scale, the calculated recurrent risk was lower than that from the EORTC scale,[37] which might be related to the special design in the CUETO scale for the patients receiving BCG intravesical immunotherapy. However, the scoring system only depending on clinical and pathological factors could not accurately evaluate the prognosis of patients with bladder cancer in T1 stage due to the independence of disease condition in each patient.[38] The markers regulated at the genetic level may judge the prognosis of patients with bladder cancer with the development of precision medicine. A reliable marker helps in recognizing the patients who have failed in BCG intravesical immunotherapy with high risk in time. Hence, these patients can undergo radical cystectomy or other treatments in time. Unfortunately, no prognostic marker has been applied in clinic currently. The results of this study potentially help to remind clinicians that patients with high expression of ki67 may need to develop more personalized follow-up plans, such as shorter follow-up and cystoscopy cycles. For patients with high risk of clinical evaluation of the guidelines and ki67 overexpression may need to promptly change the treatment strategy.

Ki67 is a nucleoprotein that can be detected in the cell cycles except G0 phase.[39] The expression of human ki67 protein is closely related to proliferation. Therefore, it is an ideal marker to confirm the growth fraction of specific cell colonies.[40] Ki67 is a widely known amplified biomarker. The ki67 monoclonal antibody can be detected by the immunohistochemical method.[41] Ki67 has been proved to be a good proliferation marker in different cancers, including bladder cancer.[42]

So far, some meta-analyses have studied the effect of ki67 on the prognostic quality of life of patients with esophageal cancer, breast cancer, epithelial ovarian cancer, and so

For peer review only - http://bmjopen.bmj.com/site/about/guidelines.xhtml

on.[43-45] Some studies have also focused on the other aspects of bladder cancer. Using meta-analysis, Luo[46] believed that a high reactivity of ki67 could predict the poor prognosis in patients with bladder cancer. The univariate analysis showed that cancer-specific survival, disease-free survival, overall survival , PFS, and RFS had a significant correlation with poor prognosis in patients with a high reactivity of ki67. However, this study enrolled all types of bladder tumors and all the therapies for NMIBC. Currently, the bladder cancer treated in the clinic is mainly NMIBC. Thus, most of the applied therapy is TUR combined with installations of chemotherapy or BCG intravesical immunotherapy based on the patients' conditions. Therefore, this analysis had a certain limitation in the prognosis of patients with NMIBC after BCG intravesical immunotherapy.

Currently, few evidence-based studies focused on the prognosis of patients with NMIBC after BCG intravesical immunotherapy. Using meta-analysis, Zhou[47] analyzed the correlation between the expression of p53 and quality of life of patients with NMIBC after BCG intravesical immunotherapy. They believed that the overexpression of p53 in patients with NMIBC treated with BCG might be associated with RFS, especially in Asian population. Similarly, Du[48] also performed the meta-analysis on the relationship between p53 status and NMIBC in T1 stage and believed that the overexpression of p53 might be related to the development of NMIBC. The present study indicated that the overexpression of ki67 was the risk factor for PFS, but the expression of ki67 had no statistically significant association with RFS. P53 is the most common inactivated tumor suppressor gene in tumor cells.[49] The inactivation of p53 may cause cell abnormal hyperplasia and cancerization. The variation in p53 results in enhanced proliferation, invasion, and

For peer review only - http://bmjopen.bmj.com/site/about/guidelines.xhtml

metabolism.[50] The increase in the expression of ki67 , as cell proliferation marker suggests enhanced proliferation.[40] As a tumor suppressor gene with complicated function, p53 has a wider range of effects. The accuracy in the prediction of quality of life may not be more appropriate compared with ki67. The genetic difference between Asians and Caucasians suggests that different prediction systems should be built for different races. Besides, p27, E2F1, ezrin, and CK20 were also studied in other investigations for predicting NMIBC prognosis, which could be explored further comparing the advantages of using them alone or combined.

However, this study still had some limitations. First, the enrolled published studies involved different populations, used similar detection equipment, and had different cutoff values. All these reasons might have caused the heterogeneity. Further, the sample size of the meta-analysis also limited its significance. Second, the meta-analysis included English published studies. Although Begg's test and Egger's test did not suggest publication bias, this study was still influenced by some bias. Finally, the surgical skills were different in different published studies, affecting the judgment regarding the effectiveness of BCG.

Conclusions

The overexpression of ki67 was the risk factor for PFS in patients with NMIBC after TUR and BCG intravesical immunotherapy, but the relationship between the expression of ki67 and RFS was not statistically significant. Owing to the aforementioned limitations of the present study, RCTs with large sample size are still required to validate the results.

Authors' contributions

For peer review only - http://bmjopen.bmj.com/site/about/guidelines.xhtml

YHH, NW, and XFZ conceived and designed the experiments. XC and YSD extracted the data. YHH, NW, and XFZ analyzed the data. ZSD, JFW, and XC contributed reagents/materials/analysis tools. YHH and NW wrote the paper. XFZ critically revised the report.

Funding

This study was supported by grants from the International S&T Cooperation Program of China (ISTCP) (Grant No.2014DFE30010).

Disclaimer

The contents of the present study are solely the responsibility of the author. The funders had no role in study design, data collection and analysis, decision to publish, or preparation of the manuscript.

Competing interests

None declared.

Data sharing statement

Datasets used and/or analyzed in the present study are available from the corresponding author on reasonable request.

References

1. Ploeg M, Aben KK, Kiemeney LA. The present and future burden of urinary bladder cancer in the world. *World J Urol* 2009;**27**:289-93. doi:10.1007/s00345-009-0383-3.

2. Torre LA, Bray F, Siegel RL, *et al.* Global cancer statistics, 2012. *CA Cancer J Clin* 2015;**65**:87-108. doi:10.3322/caac.21262.

3. Ro JY, Staerkel GA, Ayala AG. Cytologic and histologic features of superficial bladder cancer. *Urol Clin North Am* 1992;**19**:435-53.

For peer review only - http://bmjopen.bmj.com/site/about/guidelines.xhtml

4. Woldu SL, Bagrodia A, Lotan Y. Guideline of guidelines: non-muscle-invasive bladder cancer. *BJU Int* 2017;**119**:371-80. doi:10.1111/bju.13760.

5. Brausi M, Collette L, Kurth K, *et al.* Variability in the recurrence rate at first follow-up cystoscopy after TUR in stage Ta T1 transitional cell carcinoma of the bladder: a combined analysis of seven EORTC studies. *Eur Urol* 2002;**41**:523-31.

6. Jarvinen R, Kaasinen E, Sankila A, *et al.* Long-term efficacy of maintenance bacillus Calmette-Guerin versus maintenance mitomycin C instillation therapy in frequently recurrent TaT1 tumours without carcinoma in situ: a subgroup analysis of the prospective, randomised FinnBladder I study with a 20-year follow-up. *Eur Urol* 2009;**56**:260-5. doi:10.1016/j.eururo.2009.04.009.

7. Sylvester RJ, Brausi MA, Kirkels WJ, *et al.* Long-term efficacy results of EORTC genito-urinary group randomized phase 3 study 30911 comparing intravesical instillations of epirubicin, bacillus Calmette-Guerin, and bacillus Calmette-Guerin plus isoniazid in patients with intermediate- and high-risk stage Ta T1 urothelial carcinoma of the bladder. *Eur Urol* 2010;**57**:766-73. doi:10.1016/j.eururo.2009.12.024.

8. Gual Frau J, Palou J, Rodriguez O*, et al.* Failure of Bacillus Calmette-Guerin therapy in non-muscle-invasive bladder cancer: Definition and treatment options. *Arch Esp Urol* 2016;**69**:423-33.

9. Boyd LA. Intravesical Bacillus Calmette-Guerin for treating bladder cancer. *Urol Nurs* 2003;**23**:189-91, 99; quiz 92.

10. Alkhateeb SS, Neill M, Bar-Moshe S, *et al.* Long-term prognostic value of the combination of EORTC risk group calculator and molecular markers in non-muscle-invasive bladder cancer patients treated with intravesical Bacille Calmette-Guerin. *Urol Ann* 2011;**3**:119-26. doi:10.4103/0974-7796.84954.

For peer review only - http://bmjopen.bmj.com/site/about/guidelines.xhtml

11. Mariappan P, Smith G. A surveillance schedule for G1Ta bladder cancer allowing efficient use of check cystoscopy and safe discharge at 5 years based on a 25-year prospective database. *J Urol* 2005;**173**:1108-11. doi:10.1097/01.ju.0000149163.08521.69.

12. Bertz S, Otto W, Denzinger S, *et al.* Combination of CK20 and Ki-67 immunostaining analysis predicts recurrence, progression, and cancer-specific survival in pT1 urothelial bladder cancer. *Eur Urol* 2014;**65**:218-26. doi:10.1016/j.eururo.2012.05.033.

13. Kim K, Cho YM, Park BH, *et al.* Histological and immunohistochemical markers for progression prediction in transurethrally resected high-grade non-muscle invasive bladder cancer. *Int J Clin Exp Pathol* 2015;**8**:743-50.

14. Park J, Song C, Shin E, *et al.* Do molecular biomarkers have prognostic value in primary T1G3 bladder cancer treated with bacillus Calmette-Guerin intravesical therapy? *Urol Oncol* 2013;**31**:849-56. doi:10.1016/j.urolonc.2011.06.004.

15. Kruger S, Thorns C, Stocker W, *et al.* Prognostic value of MCM2 immunoreactivity in stage T1 transitional cell carcinoma of the bladder. *Eur Urol* 2003;**43**:138-45.

16. Oderda M, Ricceri F, Pisano F, *et al.* Prognostic factors including Ki-67 and p53 in Bacillus Calmette-Guerin-treated non-muscle-invasive bladder cancer: a prospective study. *Urol Int* 2013;**90**:184-90. doi:10.1159/000343431.

17. Zlotta AR, Noel JC, Fayt I, *et al.* Correlation and prognostic significance of p53, p21WAF1/CIP1 and Ki-67 expression in patients with superficial bladder tumors treated with bacillus Calmette-Guerin intravesical therapy. *J Urol* 1999;**161**:792-8.

18. Saint F, Salomon L, Quintela R, *et al.* Do prognostic parameters of remission versus relapse after Bacillus Calmette-Guerin (BCG) immunotherapy exist?. analysis

For peer review only - http://bmjopen.bmj.com/site/about/guidelines.xhtml

of a quarter century of literature. *Eur Urol* 2003;**43**:351-60; discussion 60-1.

19. Habuchi T, Marberger M, Droller MJ, *et al.* Prognostic markers for bladder cancer: International Consensus Panel on bladder tumor markers. *Urology* 2005;**66**:64-74. doi:10.1016/j.urology.2005.08.065.

20. Button KS, Ioannidis JP, Mokrysz C, *et al.* Power failure: why small sample size undermines the reliability of neuroscience. *Nat Rev Neurosci* 2013;**14**:365-76. doi:10.1038/nrn3475.

21. Liberati A, Altman DG, Tetzlaff J, *et al.* The PRISMA statement for reporting systematic reviews and meta-analyses of studies that evaluate health care interventions: explanation and elaboration. *PLoS Med* 2009;**6**:e1000100. doi:10.1371/journal.pmed.1000100.

22. Stang A. Critical evaluation of the Newcastle-Ottawa scale for the assessment of the quality of nonrandomized studies in meta-analyses. *Eur J Epidemiol* 2010;**25**:603-5. doi:10.1007/s10654-010-9491-z.

23. Parmar MK, Torri V, Stewart L. Extracting summary statistics to perform meta-analyses of the published literature for survival endpoints. *Stat Med* 1998;**17**:2815-34.

24. DerSimonian R, Laird N. Meta-analysis in clinical trials. *Control Clin Trials* 1986;**7**:177-88.

25. Higgins JP, Thompson SG. Quantifying heterogeneity in a meta-analysis. *Stat Med* 2002;**21**:1539-58. doi:10.1002/sim.1186.

26. Bax L, Ikeda N, Fukui N, *et al.* More than numbers: the power of graphs in meta-analysis. *Am J Epidemiol* 2009;169:249-55. doi:10.1093/aje/kwn340.

27. Xu X, Cheng Y, Li S, *et al.* Dietary carrot consumption and the risk of prostate cancer. *Eur J Nutr* 2014;53:1615-1623. doi: 10.1007/s00394-014-0667-2.

For peer review only

For peer review only - http://bmjopen.bmj.com/site/about/guidelines.xhtml

28. Quintero A, Alvarez-Kindelan J, Luque RJ, *et al.* Ki-67 MIB1 labelling index and the prognosis of primary TaT1 urothelial cell carcinoma of the bladder. *J Clin Pathol* 2006;**59**:83-8. doi:10.1136/jcp.2004.022939.

29. van Rhijn BW, Liu L, Vis AN, *et al.* Prognostic value of molecular markers, sub-stage and European Organisation for the Research and Treatment of Cancer risk scores in primary T1 bladder cancer. *BJU Int* 2012;**110**:1169-76. doi:10.1111/j.1464-410X.2012.10996.x.

30. Burger M, Denzinger S, Hartmann A, *et al.* Mcm2 predicts recurrence hazard in stage Ta/T1 bladder cancer more accurately than CK20, Ki67 and histological grade. *Br J Cancer* 2007;**96**:1711-5. doi:10.1038/sj.bjc.6603784.

31. Queipo-Zaragoza JA, Ruiz-Cerda JL, Vera-Donoso CD, *et al.* Prognostic value of p53, Ki-67, microstaging and microvessel density in pT1G3 bladder tumors: creation of risk groups for progression. *Scand J Urol Nephrol* 2007;**41**:283-9. doi:10.1080/00365590601183055.

32. Lopez-Beltran A, Luque RJ, Alvarez-Kindelan J, *et al.* Prognostic factors in stage T1 grade 3 bladder cancer survival: the role of G1-S modulators (p53, p21Waf1, p27kip1, Cyclin D1, and Cyclin D3) and proliferation index (ki67-MIB1). *Eur Urol* 2004;**45**:606-12. doi:10.1016/j.eururo.2003.11.011.

33. Santos L, Amaro T, Costa C, *et al.* Ki-67 index enhances the prognostic accuracy of the urothelial superficial bladder carcinoma risk group classification. *Int J Cancer* 2003;**105**:267-72. doi:10.1002/ijc.11049.

34. Blanchet P, Droupy S, Eschwege P, *et al.* Prospective evaluation of Ki-67 labeling in predicting the recurrence and progression of superficial bladder transitional cell carcinoma. *Eur Urol* 2001;**40**:169-75. doi:49768.

35. Lee E, Park I, Lee C. Prognostic markers of intravesical bacillus Calmette-Guerin

For peer review only - http://bmjopen.bmj.com/site/about/guidelines.xhtml

therapy for multiple, high-grade, stage T1 bladder cancers. *Int J Urol* 1997;**4**:552-6.

36. Babjuk M, Bohle A, Burger M*, et al.* EAU Guidelines on Non-Muscle-invasive Urothelial Carcinoma of the Bladder: Update 2016. *Eur Urol* 2017;**71**:447-61. doi:10.1016/j.eururo.2016.05.041.

37. Fernandez-Gomez J, Madero R, Solsona E, *et al.* Predicting nonmuscle invasive bladder cancer recurrence and progression in patients treated with bacillus Calmette-Guerin: the CUETO scoring model. *J Urol* 2009;**182**:2195-203. doi:10.1016/j.juro.2009.07.016.

38. Kiemeney LA, Witjes JA, Heijbroek RP, *et al.* Predictability of recurrent and progressive disease in individual patients with primary superficial bladder cancer. *J Urol* 1993;**150**:60-4.

39. Gerdes J, Lemke H, Baisch H, *et al.* Cell cycle analysis of a cell proliferation-associated human nuclear antigen defined by the monoclonal antibody Ki-67. *J Immunol* 1984;**133**:1710-5.

40. Scholzen T, Gerdes J. The Ki-67 protein: from the known and the unknown. *J Cell Physiol* 2000;**182**:311-22. doi:10.1002/(sici)1097-4652(200003)182:3<311::aid-jcp1>3.0.co;2-9.

41. Gerdes J, Li L, Schlueter C, *et al.* Immunobiochemical and molecular biologic characterization of the cell proliferation-associated nuclear antigen that is defined by monoclonal antibody Ki-67. *Am J Pathol* 1991;**138**:867-73.

42. Rodriguez-Alonso A, Pita-Fernandez S, Gonzalez-Carrero J, *et al.* p53 and ki67 expression as prognostic factors for cancer-related survival in stage T1 transitional cell bladder carcinoma. *Eur Urol* 2002;**41**:182-8; discussion 88-9.

43. McCormick Matthews LH, Noble F, Tod J, *et al.* Systematic review and meta-analysis of immunohistochemical prognostic biomarkers in resected

For peer review only - http://bmjopen.bmj.com/site/about/guidelines.xhtml

oesophageal adenocarcinoma. *Br J Cancer* 2015;**113**:107-18. doi:10.1038/bjc.2015.179.

44. Gui Y, Xu S, Yang X, *et al.* A meta-analysis of biomarkers for the prognosis of triple-negative breast cancer patients. *Biomark Med* 2016;**10**:771-90. doi:10.2217/bmm-2015-0064.

45. Qiu HL, Deng SZ, Li C, *et al.* High expression of KIF14 is associated with poor prognosis in patients with epithelial ovarian cancer. *Eur Rev Med Pharmacol Sci* 2017;**21**:239-45.

46. Luo Y, Zhang X, Mo M, *et al.* High Ki-67 Immunohistochemical Reactivity Correlates With Poor Prognosis in Bladder Carcinoma: A Comprehensive Meta-Analysis with 13,053 Patients Involved. *Medicine (Baltimore)* 2016;**95**:e3337. doi:10.1097/md.0000000000003337.

47. Zhou X, Zhang G, Tian Y. p53 Status correlates with the risk of recurrence in non-muscle invasive bladder cancers treated with Bacillus Calmette-Guerin: a meta-analysis. *PLoS One* 2015;**10**:e0119476. doi:10.1371/journal.pone.0119476.

48. Du J, Wang SH, Yang Q, *et al.* p53 status correlates with the risk of progression in stage T1 bladder cancer: a meta-analysis. *World J Surg Oncol* 2016;**14**:137. doi:10.1186/s12957-016-0890-9.

49. Fridman JS, Lowe SW. Control of apoptosis by p53. *Oncogene* 2003;**22**:9030-40. doi:10.1038/sj.onc.1207116.

50. Muller PA, Vousden KH, Norman JC. p53 and its mutants in tumor cell migration and invasion. *J Cell Biol* 2011;**192**:209-18. doi:10.1083/jcb.201009059.

Figure File

Figure 1. Flow diagram of study selection.

For peer review only - http://bmjopen.bmj.com/site/about/guidelines.xhtml

Figure 2. Forest plots of HRs estimated for the relationship between the expression of ki67 and RFS (A) or PFS (B) among patients in NMIBC treated with BCG.

Figure 3. Galbraith plot analysis was used to evaluate heterogeneity and RFS (A) or PFS (B).

Figure 4. Funnel plots of the expression of ki67 and RFS (A) or PFS (B).

Table File

Table 1. Main characteristics of all studies included in this meta-analysis

Table 2. Quality of the included studies assessed by NOS

Table 3. Subgroup results of RFS and heterogeneity test

Table 4. Subgroup results of PFS and heterogeneity test

Supplementary File

Table S1. PRISMA

Table S2. Meta-regression analysis of RFS and PFS

Table S3. Risk group stratification in NMIBC

Figure S1. Forest plots of HRs estimated for the relationship between the expression of ki67 and RFS after the aforementioned study was excluded

Figure S2. Forest plots of HRs estimated for the relationship between the expression of ki67 and PFS after the aforementioned study was excluded

For peer review only - http://bmjopen.bmj.com/site/about/guidelines.xhtml

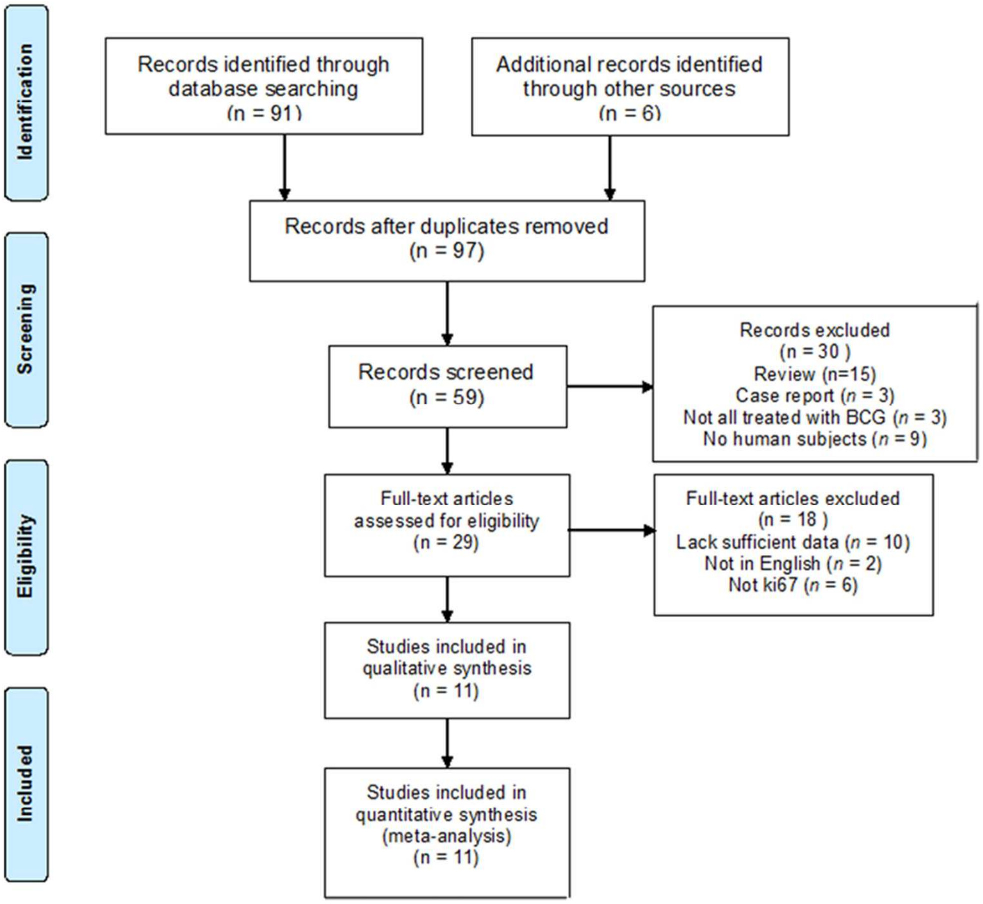

Figure 1. Flow diagram of study selection.

80x72mm (300 x 300 DPI)

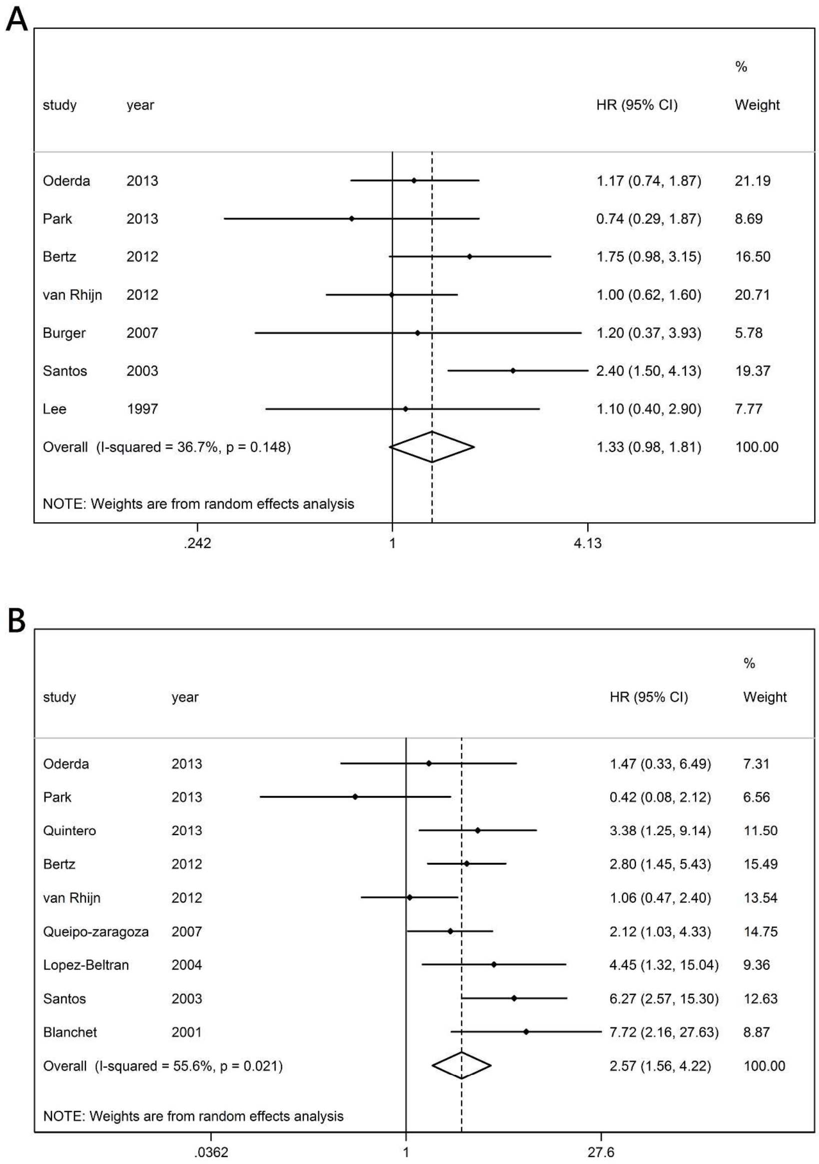

Forest plots of HRs estimated for the relationship between the expression of ki67 and RFS (A) or PFS (B) among patients in NMIBC treated with BCG.

267x381mm (300 x 300 DPI)

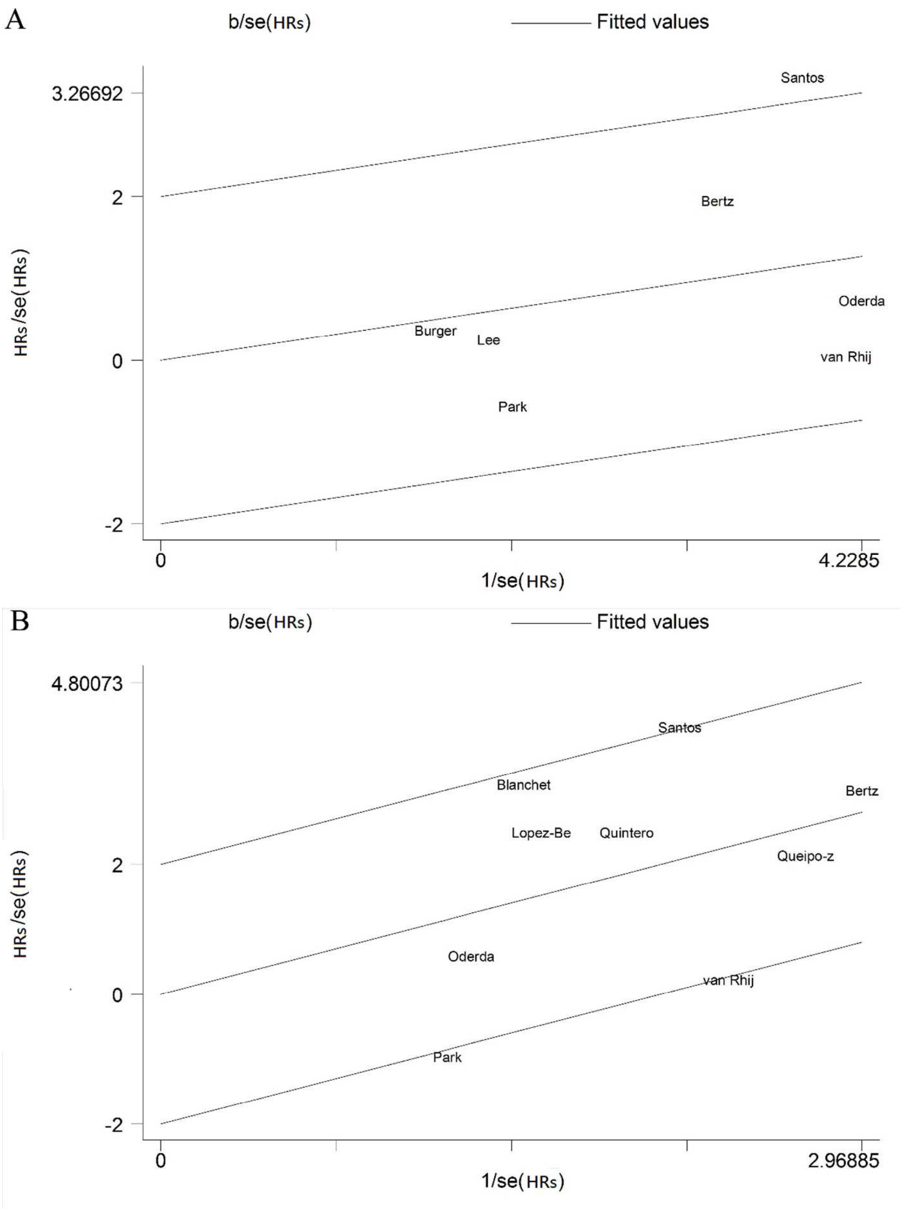

Galbraith plot analysis was used to evaluate heterogeneity and RFS (A) or PFS (B).

268x358mm (300 x 300 DPI)

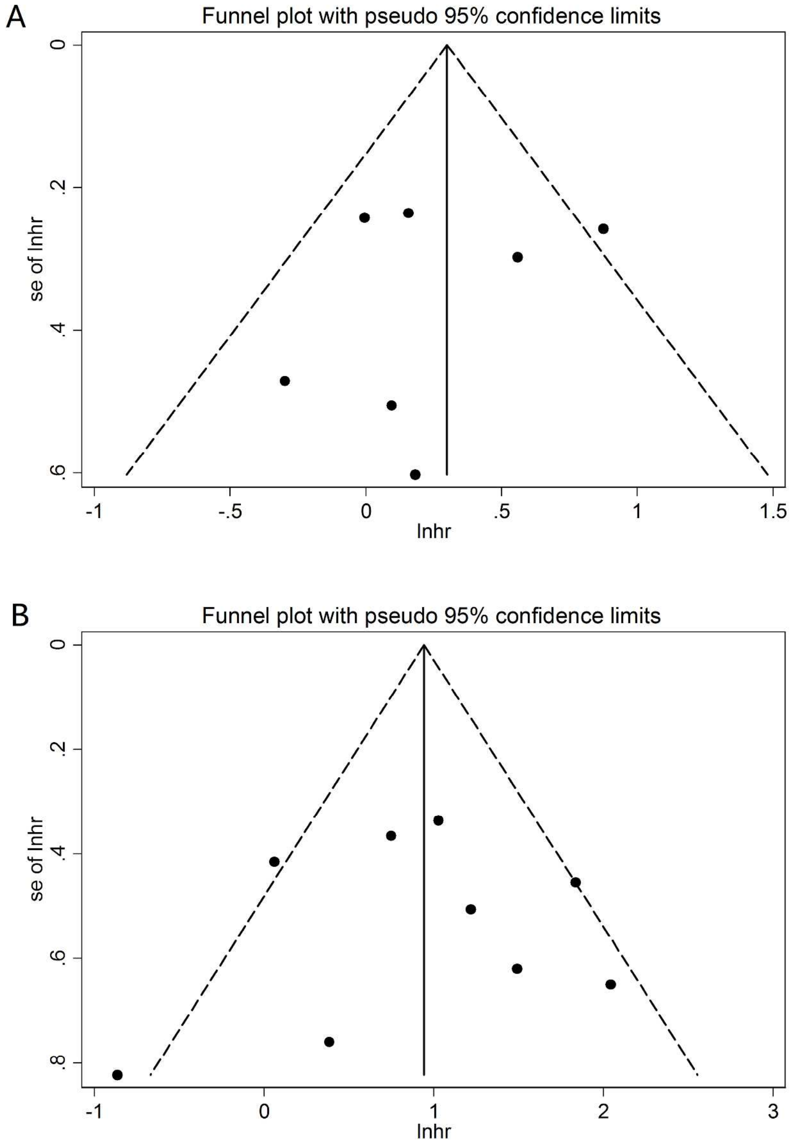

Funnel plots of the expression of ki67 and RFS (A) or PFS (B).

267x392mm (300 x 300 DPI)

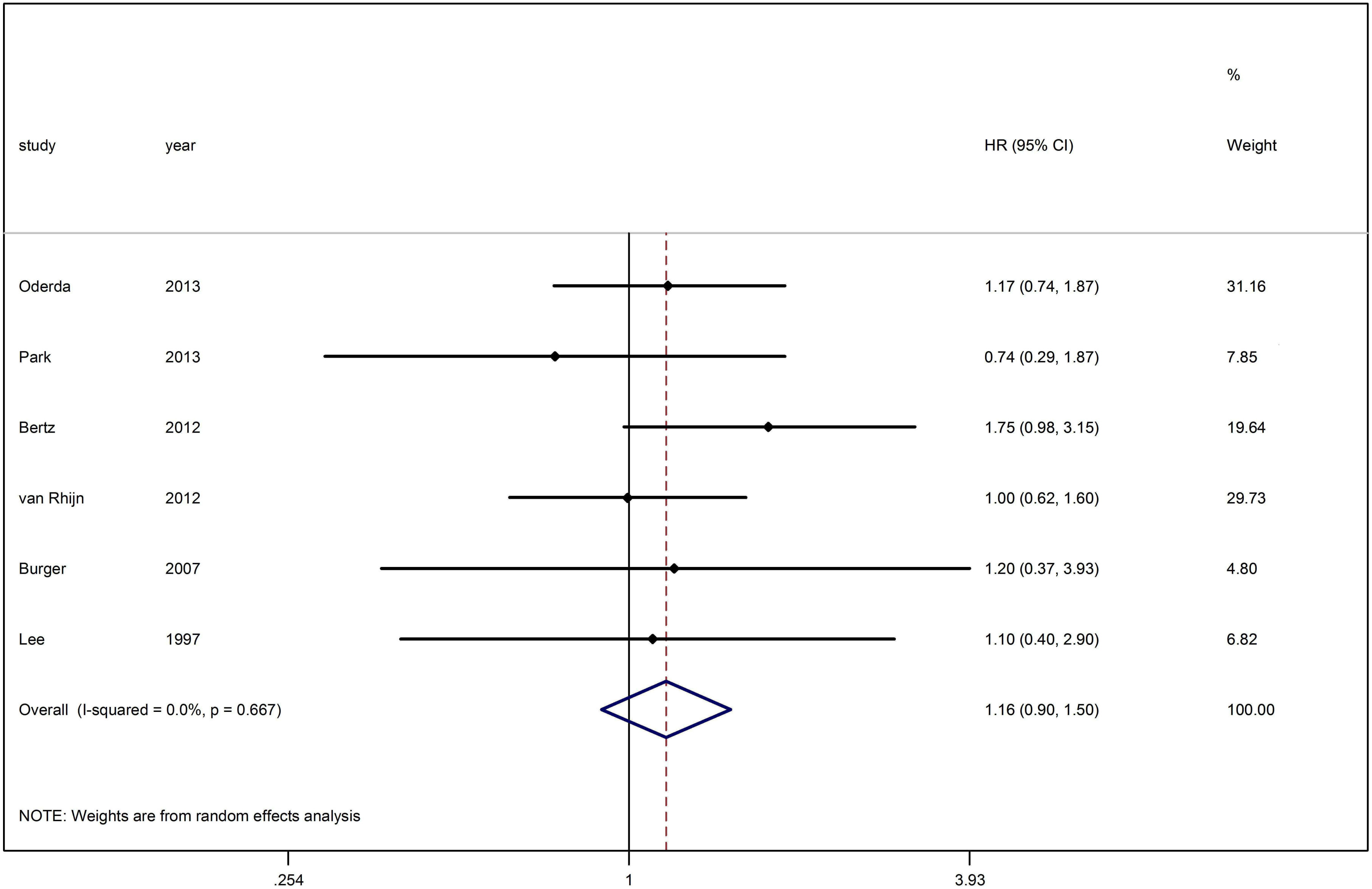

Figure S1. Forest plots of HRs estimated for the relationship between the expression of ki67 and RFS after the aforementioned study was excluded

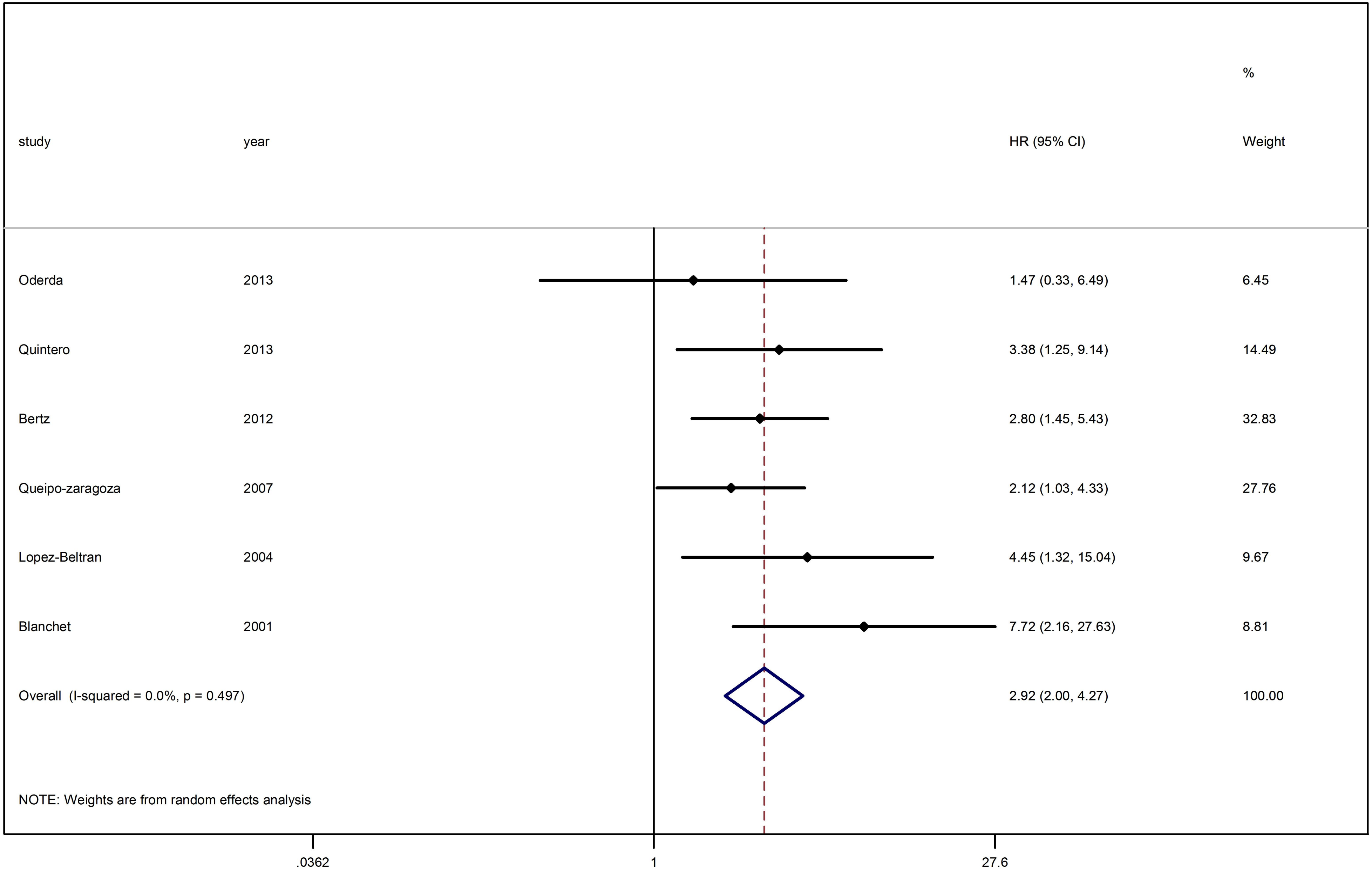

Figure S2. Forest plots of HRs estimated for the relationship between the expression of ki67 and PFS after the aforementioned study was excluded

PRISMA 2009 Checklist

Section/topic	#	Checklist item	Reported on page #
**TITLE**			
Title	1	Identify the report as a systematic review, meta-analysis, or both.	1
**ABSTRACT**			
Structured summary	2	Provide a structured summary including, as applicable: background; objectives; data sources; study eligibility criteria, participants, and interventions; study appraisal and synthesis methods; results; limitations; conclusions and implications of key findings; systematic review registration number.	3
**INTRODUCTION**			
Rationale	3	Describe the rationale for the review in the context of what is already known.	4-6
Objectives	4	Provide an explicit statement of questions being addressed with reference to participants, interventions, comparisons, outcomes, and study design (PICOS).	4-6
**METHODS**			
Protocol and registration	5	Indicate if a review protocol exists, if and where it can be accessed (e.g., Web address), and, if available, provide registration information including registration number.	7
Eligibility criteria	6	Specify study characteristics (e.g., PICOS, length of follow-up) and report characteristics (e.g., years considered, language, publication status) used as criteria for eligibility, giving rationale.	7
Information sources	7	Describe all information sources (e.g., databases with dates of coverage, contact with study authors to identify additional studies) in the search and date last searched.	7
Search	8	Present full electronic search strategy for at least one database, including any limits used, such that it could be repeated.	7
Study selection	9	State the process for selecting studies (i.e., screening, eligibility, included in systematic review, and, if applicable, included in the meta-analysis).	7-8
Data collection process	10	Describe method of data extraction from reports (e.g., piloted forms, independently, in duplicate) and any processes for obtaining and confirming data from investigators.	7-8
Data items	11	List and define all variables for which data were sought (e.g., PICOS, funding sources) and any assumptions and simplifications made.	8-9
Risk of bias in individual studies	12	Describe methods used for assessing risk of bias of individual studies (including specification of whether this was done at the study or outcome level), and how this information is to be used in any data synthesis.	9
Summary measures	13	State the principal summary measures (e.g., risk ratio, difference in means).	8-9
Synthesis of results	14	Describe the methods of handling data and combining results of studies, if done, including measures of consistency (e.g., I^2) for each meta-analysis.	8-9

For peer review only - http://bmjopen.bmj.com/site/about/guidelines.xhtml

PRISMA 2009 Checklist

Section/topic	#	Checklist item	Reported on page #
Risk of bias across studies	15	Specify any assessment of risk of bias that may affect the cumulative evidence (e.g., publication bias, selective reporting within studies).	9
Additional analyses	16	Describe methods of additional analyses (e.g., sensitivity or subgroup analyses, meta-regression), if done, indicating which were pre-specified.	9
**RESULTS**			
Study selection	17	Give numbers of studies screened, assessed for eligibility, and included in the review, with reasons for exclusions at each stage, ideally with a flow diagram.	9-10
Study characteristics	18	For each study, present characteristics for which data were extracted (e.g., study size, PICOS, follow-up period) and provide the citations.	10-12
Risk of bias within studies	19	Present data on risk of bias of each study and, if available, any outcome level assessment (see item 12).	17
Results of individual studies	20	For all outcomes considered (benefits or harms), present, for each study: (a) simple summary data for each intervention group (b) effect estimates and confidence intervals, ideally with a forest plot.	13-16
Synthesis of results	21	Present results of each meta-analysis done, including confidence intervals and measures of consistency.	9-17
Risk of bias across studies	22	Present results of any assessment of risk of bias across studies (see Item 15).	17
Additional analysis	23	Give results of additional analyses, if done (e.g., sensitivity or subgroup analyses, meta-regression [see Item 16]).	17
**DISCUSSION**			
Summary of evidence	24	Summarize the main findings including the strength of evidence for each main outcome; consider their relevance to key groups (e.g., healthcare providers, users, and policy makers).	17-22
Limitations	25	Discuss limitations at study and outcome level (e.g., risk of bias), and at review-level (e.g., incomplete retrieval of identified research, reporting bias).	22
Conclusions	26	Provide a general interpretation of the results in the context of other evidence, and implications for future research.	22
**FUNDING**			
Funding	27	Describe sources of funding for the systematic review and other support (e.g., supply of data); role of funders for the systematic review.	23

From: Moher D, Liberati A, Tetzlaff J, Altman DG, The PRISMA Group (2009). Preferred Reporting Items for Systematic Reviews and Meta-Analyses: The PRISMA Statement. PLoS Med 6(6): e1000097. doi:10.1371/journal.pmed1000097

For more information, visit: **www.prisma-statement.org**.

For peer review only - http://bmjopen.bmj.com/site/about/guidelines.xhtml

Table S2. Meta-regression analysis of RFS and PFS

PFS

Heterogeneity factor	Coefficient	SE	*t*	*P*
Years	–0.0343	0.0283	–1.21	0.280
Country				
1	–0.3899	0.3716	–1.05	0.404
2	–0.7185	0.3503	–2.05	0.177
3	–0.9900	0.4307	–2.30	0.148
4	–0.8805	0.3541	–2.49	0.131
Numbers of patients	0.0022	0.0020	1.16	0.299
Stage	None			
Cutoff	–0.0098	0.0407	–0.24	0.819
Age	0.0021	0.0395	0.05	0.959
Follow-up	–0.0062	0.0065	–0.96	0.379

PFS

Heterogeneity factor	Coefficient	SE	*t*	*P*
Years	–0.1195	0.0461	–2.59	0.036
Country				

For peer review only

1	0.2080	0.7936	0.26	0.818
2	–0.8062	0.5662	–1.42	0.290
3	–1.4505	0.8858	–1.64	0.243
4	–2.7009	0.9407	–2.87	0.103
5	–1.7766	0.6167	–2.88	0.102
6	–0.8158	0.5281	–1.54	0.262
Numbers of patients	0.0006	0.0036	0.16	0.877
Stage				
1	–1.4505	1.5909	–0.91	0.458
2	–0.8062	1.4108	–0.57	0.625
3	0.2080	1.5332	0.14	0.904
4	–1.7766	1.4353	–1.24	0.341
5	–1.2560	1.2069	–1.04	0.407
6	–0.6170	1.4689	–0.42	0.715
Cutoff	–0.0177	0.0309	–0.57	0.585
Age	–0.0672	0.0757	–0.89	0.404
Follow-up	–0.0118	0.0159	–0.74	0.483

For peer review only

Table S3. Risk group stratification in NMIBC

**Low-risk tumors**	Primary, solitary, Ta, LG/G1, <3 cm, no CIS
**Intermediate-risk tumors**	All tumors not defined in the two adjacent categories (between the category of low and high risk)
**High-risk tumors**	Any of the following:<br>T1 tumor<br>HG/G3 tumor<br>CIS<br>Multiple, recurrent, and large (>3 cm) Ta G1G2 tumors (all conditions must be present at this point)

NMIBC, non-muscle-invasive bladder cancer; CIS, carcinoma in situ; HG, high grade; LG, low grade.

For peer review only

PRISMA 2009 Checklist

Section/topic	#	Checklist item	Reported on page #
**TITLE**			
Title	1	Identify the report as a systematic review, meta-analysis, or both.	1
**ABSTRACT**			
Structured summary	2	Provide a structured summary including, as applicable: background; objectives; data sources; study eligibility criteria, participants, and interventions; study appraisal and synthesis methods; results; limitations; conclusions and implications of key findings; systematic review registration number.	3
**INTRODUCTION**			
Rationale	3	Describe the rationale for the review in the context of what is already known.	4-6
Objectives	4	Provide an explicit statement of questions being addressed with reference to participants, interventions, comparisons, outcomes, and study design (PICOS).	4-6
**METHODS**			
Protocol and registration	5	Indicate if a review protocol exists, if and where it can be accessed (e.g., Web address), and, if available, provide registration information including registration number.	7
Eligibility criteria	6	Specify study characteristics (e.g., PICOS, length of follow-up) and report characteristics (e.g., years considered, language, publication status) used as criteria for eligibility, giving rationale.	7
Information sources	7	Describe all information sources (e.g., databases with dates of coverage, contact with study authors to identify additional studies) in the search and date last searched.	7
Search	8	Present full electronic search strategy for at least one database, including any limits used, such that it could be repeated.	7
Study selection	9	State the process for selecting studies (i.e., screening, eligibility, included in systematic review, and, if applicable, included in the meta-analysis).	7-8
Data collection process	10	Describe method of data extraction from reports (e.g., piloted forms, independently, in duplicate) and any processes for obtaining and confirming data from investigators.	7-8
Data items	11	List and define all variables for which data were sought (e.g., PICOS, funding sources) and any assumptions and simplifications made.	8-9
Risk of bias in individual studies	12	Describe methods used for assessing risk of bias of individual studies (including specification of whether this was done at the study or outcome level), and how this information is to be used in any data synthesis.	9
Summary measures	13	State the principal summary measures (e.g., risk ratio, difference in means).	8-9
Synthesis of results	14	Describe the methods of handling data and combining results of studies, if done, including measures of consistency (e.g., I^2) for each meta-analysis.	8-9

For peer review only

For peer review only - http://bmjopen.bmj.com/site/about/guidelines.xhtml

PRISMA 2009 Checklist

Section/topic	#	Checklist item	Reported on page #
Risk of bias across studies	15	Specify any assessment of risk of bias that may affect the cumulative evidence (e.g., publication bias, selective reporting within studies).	9
Additional analyses	16	Describe methods of additional analyses (e.g., sensitivity or subgroup analyses, meta-regression), if done, indicating which were pre-specified.	9
**RESULTS**			
Study selection	17	Give numbers of studies screened, assessed for eligibility, and included in the review, with reasons for exclusions at each stage, ideally with a flow diagram.	9-10
Study characteristics	18	For each study, present characteristics for which data were extracted (e.g., study size, PICOS, follow-up period) and provide the citations.	10-12
Risk of bias within studies	19	Present data on risk of bias of each study and, if available, any outcome level assessment (see item 12).	17
Results of individual studies	20	For all outcomes considered (benefits or harms), present, for each study: (a) simple summary data for each intervention group (b) effect estimates and confidence intervals, ideally with a forest plot.	13-16
Synthesis of results	21	Present results of each meta-analysis done, including confidence intervals and measures of consistency.	9-17
Risk of bias across studies	22	Present results of any assessment of risk of bias across studies (see Item 15).	17
Additional analysis	23	Give results of additional analyses, if done (e.g., sensitivity or subgroup analyses, meta-regression [see Item 16]).	17
**DISCUSSION**			
Summary of evidence	24	Summarize the main findings including the strength of evidence for each main outcome; consider their relevance to key groups (e.g., healthcare providers, users, and policy makers).	17-22
Limitations	25	Discuss limitations at study and outcome level (e.g., risk of bias), and at review-level (e.g., incomplete retrieval of identified research, reporting bias).	22
Conclusions	26	Provide a general interpretation of the results in the context of other evidence, and implications for future research.	22
**FUNDING**			
Funding	27	Describe sources of funding for the systematic review and other support (e.g., supply of data); role of funders for the systematic review.	23

From: Moher D, Liberati A, Tetzlaff J, Altman DG, The PRISMA Group (2009). Preferred Reporting Items for Systematic Reviews and Meta-Analyses: The PRISMA Statement. PLoS Med 6(6): e1000097. doi:10.1371/journal.pmed1000097

For more information, visit: **www.prisma-statement.org**.

For peer review only - http://bmjopen.bmj.com/site/about/guidelines.xhtml

BMJ Open

Prognostic Value of ki67 in Bacillus Calmette–Guérin-Treated Non-muscle-Invasive Bladder Cancer: a Meta-analysis and Systematic Review

Journal:	*BMJ Open*
Manuscript ID	bmjopen-2017-019635.R2
Article Type:	Research
Date Submitted by the Author:	14-Feb-2018
Complete List of Authors:	He, Yuhui; Peking University China-Japan Friendship School of Clinical Medicine; China-Japan Friendship Hospital, Department of Urology<br>Wang, Ning; North China University of Science and Technology<br>Zhou, Xiaofeng; Peking University China-Japan Friendship School of Clinical Medicine; China-Japan Friendship Hospital, Department of Urology<br>Wang, Jianfeng; China-Japan Friendship Hospital, Department of Urology<br>Ding, Zhenshan; China-Japan Friendship Hospital, Department of Urology<br>Chen, Xing ; China-Japan Friendship Hospital, Department of Urology<br>Deng, Yisen; Peking University China-Japan Friendship School of Clinical Medicine; China-Japan Friendship Hospital, Department of Urology
**Primary Subject Heading**:	Urology
Secondary Subject Heading:	Urology
Keywords:	ki67, meta-analysis, non-muscular-invasive bladder cancer, prognosis

SCHOLARONE™ Manuscripts

Prognostic Value of ki67 in Bacillus Calmette–Guérin-Treated Non-muscle-Invasive Bladder Cancer: a Meta-analysis and Systematic Review

Yuhui He[1, 2], Ning Wang[3], Xiaofeng Zhou[1, 2*], Jianfeng Wang[2], Zhenshan Ding[2], Xing Chen[2], Yisen Deng[1, 2]

[1] Peking University China-Japan Friendship School of Clinical Medicine, Beijing 100029, China

[2] Department of Urology, China-Japan Friendship Hospital, Beijing 100029, China

[3] North China University of Science and Technology, Tangshan 063013, China

***Corresponding Author**

Xiaofeng Zhou

Peking University China-Japan Friendship School of Clinical Medicine, Beijing 100029, China

Department of Urology, China-Japan Friendship Hospital, Beijing 100029, China

Tel: +86 13121391766

Email: doctorzxf@126.com

Fax: +86 64217749

Word count

Title page: 98

Abstract: 251

References: 1403

For peer review only - http://bmjopen.bmj.com/site/about/guidelines.xhtml

For peer review only

Number of tables, figures, supplementary files: 4, 4, 6

For peer review only

For peer review only - http://bmjopen.bmj.com/site/about/guidelines.xhtml

Abstract

Objectives: The aim of this study was to explore the prognostic value of ki67 as a marker in non-muscle-invasive bladder cancer (NMIBC) patients treated with Bacillus Calmette–Guérin (BCG).

Methods: Studies were systematically retrieved from the relevant databases (Web of Science, PubMed, Cochrane Library, and Embase), and the expiry date was May 2017. The research steps referred to the Preferred Reporting Items for Systematic Reviews and Meta-Analysis statement.

Results: A total of 11 studies that complied with the inclusion criteria were enrolled. The expression of ki67 was not statistically significantly associated with recurrence-free survival (RFS) [hazard ratio (HR): 1.331; 95% CI: 0.980–1.809)]. No significant heterogeneity was found among all included studies (I^2 = 36.7%, P = 0.148). The expression of ki67 was statistically significantly associated with progression-free survival (PFS) (HR: 2.567; 95% CI: 1.562–4.219), and the overexpression of ki67 was the risk factor for PFS. Significant heterogeneity was noted among all the included studies (I^2 = 55.6%, P = 0.021). The studies that might cause heterogeneity were excluded using the Galbraith plot, and then the meta-analysis was performed again. The results showed that the expression of ki67 was still associated with PFS (HR: 2.922; 95% CI: 2.002–4.266).

Conclusions: The overexpression of ki67 was the risk factor for PFS, and the relationship between the expression of ki67 and RFS was not statistically significant in patients with NMIBC treated with BCG intravesical immunotherapy. Well-designed, prospective, randomized controlled trials with a large sample size are still needed to validate the findings.

Key words: ki67; meta-analysis; non-muscular-invasive bladder cancer; prognosis

For peer review only - http://bmjopen.bmj.com/site/about/guidelines.xhtml

Strengths and limitations of this study

This meta-analysis and systematic review was performed via a strict literature search. It was the first meta-analysis to evaluate the prognostic value of ki67 in patients with NMIBC after transurethral resection and BCG intravesical immunotherapy.

The number of studies considered in the final meta-analysis was 11. This small sample size limited the potential analyses. The research did not consider the surgical skills mentioned in published studies.

Despite a systematic search strategy, the inclusion criteria excluded non-English documents and had language bias. The meta-regression analysis suggested no bias, but a selection bias was likely.

These limitations notwithstanding, the research can guide the follow-up research on immunohistochemical markers and clinical practice in non-muscular-invasive bladder cancer.

Introduction

Bladder cancer is one of the most common clinical urological tumors. It is a direct threat to the survival of patients with the disease. The incidence of bladder cancer varies across the world, with the highest rate in the developed communities.[1] A total of 429,800 new cases of bladder cancer and 165,100 deaths occurred in 2012 worldwide. Bladder cancer occurs mostly in men, and about a tenfold variation in incidence rates has been reported internationally.[2] About 70% of these patients have non-muscle-invasive bladder cancer (NMIBC).[3] Four major organizational guidelines on NMIBC, including the American Urological Association/Society of Urologic Oncology, the European Association of Urology, the National Comprehensive Cancer

For peer review only - http://bmjopen.bmj.com/site/about/guidelines.xhtml

Network, and the National Institute for Health and Care Excellence guidelines, recommend that the proper initial transurethral resection (TUR) of bladder tumor is a critical step in the initial management and staging of the disease.[4] However, TUR surgery alone cannot solve the postoperative problems for NMIBC because of high recurrence rate and disease development.[5] Postoperative TUR associated with Bacillus Calmette–Guérin (BCG) intravesical immunotherapy can prevent the postoperative recurrence of NMIBC and significantly reduce the moderate and high development risk of NMIBC.[6, 7] However, the postoperative BCG intravesical immunotherapy still has some problems. The failure rate of BCG intravesical therapy in NMIBC is about 40%–50%.[8] Furthermore, BCG has toxic side effects, such as hepatitis, pneumonitis, epididymitis/orchitis, abscess formation, bladder contracture, ureteral obstruction, BCG sepsis, leukopenia, and hematuria.[9] Therefore, BCG therapy should be individually performed, and the patients in whom BCG therapy is ineffective should be timely recognized. These patients or those with poor prognosis should receive radical cystectomy or any other therapy in time to avoid futile treatment and alleviate pain. However, the recognition of patients with no effect of TUR postoperative BCG intravesical immunotherapy is still hard due to the heterogeneity of bladder cancer and individuality of patients.[10] Therefore, finding the prognostic factors for patients with NMIBC receiving TUR and BCG therapy is extremely necessary.

The recurrence rate of bladder cancer treated with different therapies is between 50% and 80%, and about 15% of the low-grade tumor recurrence involves high-grade tumors.[11] The patients need periodical cystoscopy to find the recurrent focus in time. A reliable prognostic molecular marker can reduce the pain caused by cystoscopy. Because NMIBC does not have reliable prognostic markers, it is hard to decide

For peer review only

postoperative therapy in the clinic,[12] which depends mainly on the clinical guidelines and physician's experience. Currently, some of the published studies about immunohistochemical markers have evaluated the prognostic value of BCG intravesical immunotherapy on the patients first receiving TUR. The main immunohistochemical markers include ki67, p53, p27, pRb, CD9, CD20, E2F1, and so forth.[13, 14] However, no immunohistochemical marker has been confirmed so far. The prognostic value of ki67 antigen on the survival in patients with NMIBC receiving BCG intravesical immunotherapy has been controversial. For example, Kruger[15] reported that ki67 antigen was an independent predictive factor for the recurrence of pT1 stage tumor, but Oderde[16] believed that ki67 was an independent predictive factor for the recurrence of all NMIBCs. Zlotta[17] reported that ki67 antigen had no independent prognostic value in patients receiving BCG therapy. Saint[18] retrospected the recent 25-year published studies and believed that the independent prognostic factor for bladder cancer in patients receiving BCG therapy was not clear. An international consensus group listed various bladder cancer prognostic indexes by reviewing PubMed and considered that although some markers (such as ki67 and p53) could predict the recurrence and development of bladder cancer, the data still had heterogeneity. Thus, strict test criteria and clear statistical methods should be established for further evaluation.[19]

A meta-analysis can enlarge the sample size by integrating independent studies with small sample size, further increase the statistical efficacy, and reduce the wrong conclusion caused by the small sample size.[20] The aim of this study was to explore the prognostic value of ki67 as a marker in patients with NMIBC treated with BCG. Based on the literature search, this study was the first meta-analysis to evaluate the prognostic value of ki67 in patients with NMIBC treated with BCG.

For peer review only - http://bmjopen.bmj.com/site/about/guidelines.xhtml

Methods

This meta-analysis was performed according to the Preferred Reporting Items for Systematic Reviews and Meta-Analyses (PRISMA) statement (**Table S1**).[21] The present meta-analysis did not need the approval because all the enrolled published studies were approved by the ethics committee in there research institute.

Literature retrieval strategy

The comprehensive literature search was performed on Web of Science, PubMed, Cochrane Library, and Embase databases for relevant studies. The last quest was updated on May 24, 2017, with hand-searching to identify any potentially eligible studies that might have been missed. The following search strategy was adopted for each database: ("Urinary Bladder Neoplasms"[Mesh] OR "bladder cancer" OR "bladder carcinoma" OR " bladder tumor ") AND ("BCG Vaccine"[Mesh] OR "BCG" OR "Bacillus Calmette–Guérin") AND ("ki67 antigen "[Mesh] OR " ki-67" OR " ki67" OR " MBI-1"). Filters were as follows: retrospective, array research, clinical trial, controlled clinical trial, and randomized controlled trial. Free word retrieval strategy was used. The enrolled contents included the reference lists and relevant suggestive references while searching (File S1).

Inclusion and exclusion criteria

The inclusion criteria were as follows: (1) prospective or retrospective published studies evaluating the prognostic relationship between the expression of ki67 and NMIBC treated with BCG; (2) the expression of ki67 in tissues detected by immunohistochemistry analysis; (3) hazard ratio (HR) and 95% confidence interval (95% CI) directly obtained from the published studies; and (4) published English studies. The exclusion criteria were as follows: (1) review, systematic evaluation, case

For peer review only - http://bmjopen.bmj.com/site/about/guidelines.xhtml

report, editorial, and specialist experience; (2) studies with no human subjects; and (3) published studies in which data could not be extracted or those having wrong data.

Data extraction and evaluation of literature quality

Based on the aforementioned criteria, two reviewers independently screened the published studies by reading titles and abstracts and got preliminary conclusions. If the conclusions were not consistent, the literature was discussed by all the authors to decide its enrollment. The relevant information of the enrolled published studies was extracted, such as first author, publication time, research country, sex, case number, age, follow-up date, disease stage, cutoff values, recurrence-free survival (RFS), and progression-free survival (PFS). The Newcastle–Ottawa Scale (NOS) was used to evaluate the quality of all the published studies.[22] Scores 0–3, 4–5, and 6–8 were accepted as low, medium, and high quality, respectively.

Statistical methods

The measuring time and method of ki67 complied with the standard of clinical routine and pathological examination. Tumor tissue samples were taken in accordance with the standard surgical procedure and used for immunohistochemical analysis. RFS and PFS were the traditionally used statistical parameters. PFS was defined as the time from the beginning of treatment to the first progression. RFS was defined as the time from the removal of the lesion (or the randomization of the clinical trial) until the recurrence or death of the tumor. The impact of the expression of ki67 on survival was quantified using the combined HRs and 95% CIs. The HR and 95% CI of each study were directly extracted from each original published study. Besides, the Parmar and Tierney's[23] method was used to extract the data because some of the published studies did not directly provide HR and 95% CI. For example, some studies provided only the survival curve. In this meta-analysis, the DerSimonian–Laird random-effects model[24]

For peer review only - http://bmjopen.bmj.com/site/about/guidelines.xhtml

was used because when the heterogeneity was large, only the random-effects model could be suitably used. Similar to traditional methods, HR >1 was considered as the prognostic risk factor for the overexpression of ki67, and HR <1 was a protective factor. 95% CI <1 indicated a statistically significant difference in the relationship between the overexpression of ki67 and prognosis.

The heterogeneity was calculated according to chi-square-based Q test and I^2 statistic.[25] The heterogeneity was judged using the I^2 value (low heterogeneity: I^2 <25%; moderate heterogeneity: I^2 = 25%–50%; large heterogeneity: I^2 >50%). Besides, A P value >0.05 was considered as low heterogeneity. Then, the subgroup analysis based on regions, sample size, follow-up period, tumor grading, cutoff value, publication time, and patient age was performed. A value of 1% was considered to be a statistically significant level in the subgroup analysis. A Galbraith plot was used to search published studies with heterogeneity[26], and the meta-analysis was performed again after excluding these published studies. Meanwhile, the factors causing heterogeneity were also explored using the residual maximum likelihood (REML)-based random-effects meta-regression analysis.[27] All the statistical analyses were performed using the Stata12.0 software (StataCorp, TX, USA), and the two-sided test was used to evaluate the P value.

Evaluation of publication bias

Begg’s plot and Egger's test method were used to find the possible publication bias. A P value <0.05 was considered to indicate publication bias.

Results

Literature screening

A total of 97 published studies were retrieved. Furthermore, 68 of them were excluded

For peer review only - http://bmjopen.bmj.com/site/about/guidelines.xhtml

after duplicates were removed and records screened, and 18 were excluded after reading the full text (10 published studies from which HR and 95% CI could not be obtained, 2 non-English studies, and 6 that did not use ki67 detection). Finally, 11 published studies were enrolled in the meta-analysis (**Figure 1**).

Basic characteristics and quality evaluation of enrolled published studies

The enrolled 11 published studies were published between 1997 and 2013, and the countries included Italy, South Korea, Spain, Germany, New Zealand, Canada, Portugal, and France. The largest sample size was 309, and the smallest one was 32. A total of 1321 patients were enrolled in this study. The follow-up period was more than 36 months, and the longest was 229 months. T1 was the main tumor grading, and the cutoff value ranged from 10.4% to 40%. Seven published studies reported patients' RFS, and nine reported PFS (**Table 1**). One literature was scored as 6 star by NOS, seven as 7 star, and three as 8 star. The median of the NOS score was 7 (**Table 2**).

For peer review only

Table 1. Main characteristics of all studies included in this meta-analysis

Study	Year	Country	Male/ Female	No. of patients	Age (year)	Follow-up (month)	Stage	Cutoff	Survival analysis
Oderda[16]	2013	Italy	166/26	192	73.2 (SD 11.9)	100 (2–229)	All NMIBC	20%	RFS
Park[14]	2013	Korea	53/8	61	66 (31–85)	60 (6–217)	T1G3	10.4%	RFS/PFS
Quintero[28]	2013	Spain	143/21	164	61 (29–93)	75 (60–144)	Ta	13%	PFS
Bertz[12]	2012	Germany	237/72	309	71.7 (38–87)	49 (5–172)	pT1	15%	RFS/PFS
van Rhijn[29]	2012	Netherlands, Canada	105/24	129	68.8 (SD 9.9)	78 (39.6-110.4)	T1	25%	RFS/PFS
Burger[30]	2007	Germany	45/21	71	71 (52–94)	39 (1–133)	T1/Ta	15%	RFS
Queipo-zaragoza[31]	2007	Spain	71/12	83	68.1 (SD 8.5)	All >36	T1G3	40%	PFS
Lopez-Beltran[32]	2004	Spain	49/2	51	69.96 (49–89)	63.82 (60–144)	T1G3	13%	PFS
Santos[33]	2003	Portugal	115/44	159	66 (21–88)	46.5 (4–123)	pTa/pT1	18%	RFS/PFS
Blanchet[34]	2001	France	-	70	62.6 (21–84)	64 (12–111)	pT1/pTa	13%	PFS
Lee[35]	1997	Korea	28/4	32	57.1 (30–81)	All >24	T1G2-3	20%	RFS

NMIBC, Non-muscle-invasive bladder cancer; no., number; PFS, progression-free survival; RFS, recurrence-free survival.

For peer review only - http://bmjopen.bmj.com/site/about/guidelines.xhtml

Table 2. Quality of the included studies assessed by NOS

Study	Selection				Comparability	Exposure			Scores
	Adequate definition of cases	Representativeness of cases	Selection of controls	Definition of controls	Control for important factor	Ascertainment of exposure	Same method to ascertain for cases and controls	Non-response rate	
Oderda[16]	—	☆	☆	☆	☆☆	☆	☆	☆	8
Park[14]	—	☆	☆	—	☆☆	☆	☆	☆	7
Quintero[28]	—	☆	☆	—	☆☆	☆	☆	—	6
Bertz[12]	—	☆	☆	—	☆☆	☆	☆	☆	7
van Rhijn[29]	—	☆	☆	—	☆☆	☆	☆	☆	7
Burger[30]	☆	☆	☆	—	☆☆	☆	☆	—	7
Queipo-zaragoza[31]	—	☆	☆	—	☆☆	☆	☆	☆	7
Lopez-Beltran[32]	—	☆	☆	☆	☆☆	☆	☆	☆	8
Santos[33]	—	☆	☆	—	☆☆	☆	☆	☆	7
Blanchet[34]	—	☆	☆	☆	☆☆	☆	☆	☆	8
Lee[35]	—	☆	☆	—	☆☆	☆	☆	☆	7

For peer review only

For peer review only - http://bmjopen.bmj.com/site/about/guidelines.xhtml

Influence of the expression of ki67 on RFS

Seven published studies reported the expression of ki67 and PFS results of patients with NMIBC treated with BCG. The meta-analysis indicated that ki67 had no statistically significant association with RFS (HR: 1.331; 95% CI: 0.980–1.809), and no heterogeneity among the enrolled studies was reported (I^2 = 36.7%, P = 0.148) (**Figure 2A**). The subgroup analysis was performed based on the regions, sample size, follow-up period, stage, cutoff value, publication time, and age. Meanwhile, all the original published analyses on the association between the expression of ki67 and RFS in patients with NMIBC treated with BCG were multivariate, and the HRs were adjusted. The stratification analysis by region indicated that ki67 was also significantly associated with RFS in Caucasians and a follow-up period shorter than 6 months. (HR: 1.441, 95% CI: 1.014–2.047; HR: 1.853, 95% CI: 1.316–2.607) (**Table 3**).

For peer review only

For peer review only - http://bmjopen.bmj.com/site/about/guidelines.xhtml

Table 3. Subgroup results of RFS and heterogeneity test

Variables	Analysis number	HR (95% CI)	Heterogeneity test		
			Q	*P*	I^2 (%)
Total RFS	7	1.331 (0.980–1.809)	9.48	0.148	36.7
Region					
Asian	2	0.892 (0.454–1.752)	0.32	0.570	0.0
Caucasian	5	1.441 (1.014–2.047)	7.52	0.111	46.8
Sample size					
>100	4	1.466 (0.986–2.181)	7.44	0.059	59.7
≤100	3	0.959 (0.534–1.725)	0.51	0.777	0.0
Follow-up (month)					
≥60	3	1.036 (0.758–1.415)	0.79	0.674	0.0
<60	4	1.853 (1.316–2.607)	2.62	0.453	0.0
Stage					
All NMIBC	3	1.575 (0.915–2.711)	4.44	0.109	54.9
Others	4	1.153 (0.821–1.620)	3.21	0.360	6.6
Cutoff					
15%	2	1.625 (0.963–2.743)	0.31	0.575	0.0
Others	5	1.252 (0.839–1.869)	8.56	0.073	53.3
Publication year					
≥2012	4	1.164 (0.874,1.550)	3.20	0.362	6.3
<2012	3	1.774 (1.046,3.008)	2.57	0.277	22.1
Patient age (year)					
≥70	3	1.352 (0.955–1.913)	1.16	0.559	0.0
<70	4	1.256 (0.717–2198)	8.32	0.040	63.9

NMIBC, Non-muscle-invasive bladder cancer; RFS, recurrence-free survival.

For peer review only

For peer review only - http://bmjopen.bmj.com/site/about/guidelines.xhtml

Influence of the expression of ki67 on PFS

A total of nine published studies reported the expression of ki67 and PFS results of patients in NMIBC treated with BCG. The meta-analysis indicated that ki67 had no statistically significant association with RFS (HR:2.567, 95% CI: 1.562–4.219), and the overexpression of ki67 was the risk factor for PFS. Statistically significant heterogeneity was found among all the included studies (I^2 = 55.6%, P = 0.021) (**Figure 2B**). The subgroup analysis was performed based on the regions, sample size, follow-up period, stage, cutoff value, publication time, and age. However, the data extracted from six original published analyses on the association between the expression of ki67 and PFS in patients with NMIBC treated with BCG were multivariate with adjusted HRs, whereas the data from three original published analyses were univariate with unadjusted HRs. In the stratified analyses by the region, sample size, follow-up time, stage, cutoff, publication year, and patient age, significant associations were observed in the studies with Caucasian subgroup, sample size >100, follow-up period <6 months, other cutoffs, and two subgroups based on age (HR: 1.97, 95% CI: 1.04–3.74; HR: 2.37, 95% CI: 1.23–4.55; HR:2.49, 95% CI: 1.19–5.21; HR: 2.515, 95% CI: 1.382–4.576; HR: 2.800, 95% CI: 1.447–5.418; and HR: 2.654, 95% CI: 1.381–5.100, respectively). However, significant associations were also observed in both multivariate and univariate analyses (HR: 2.10, 95% CI: 1.07–1.12; HR: 2.80, 95%CI: 1.65–7.85, respectively), and the effect size suggested the same outcomes (HR: 2.567, 95% CI: 1.562–4.219) (**Table 4**).

For peer review only - http://bmjopen.bmj.com/site/about/guidelines.xhtml

Table 4. Subgroup results of PFS and heterogeneity test

Variables	Analysis number	HR (95% CI)	Heterogeneity test		
			Q	*P*	I^2 (%)
Total PFS	9	2.567 (1.562–4.219)	18.1	0.021	55.6
Region					
Asian	1	0.421 (0.084–2.114)	0.00		
Caucasian	8	2.883 (1.830–4.544)	12.99	0.072	46.1
Sample size					
>100	5	2.559 (1.372–4.774)	9.26	0.055	56.8
≤100	4	2.536 (0.943–6.818)	8.75	0.033	65.7
Follow-up (month)					
≥60	6	2.153 (0.984–4.710)	13.08	0.023	61.8
<60	3	3.158 (1.774–5.623)	3.56	0.169	43.8
Stage					
All NMIBC	3	4.673 (1.938–11.264)	3.29	0.193	39.2
Others	6	2.044 (1.213–15.040)	9.58	0.088	47.8
Cut off					
15%	1	2.800 (1.447–5.418)	0.00		
Others	8	2.515 (1.382–4.576)	17.92	0.012	60.9
Publication year					
≥2012	5	1.685（0.883,3.215）	8.04	0.090	50.2
<2012	4	4.176（2.209,7.884）	5.00	0.172	40.0
Patient age (year)					
≥70	2	2.519 (1.377–4.606)	0.60	0.438	0.0
<70	7	2.654 (1.381–5.100)	17.40	0.008	65.5
Multivariate/Univariate					
Multivariate	6	2.101 (1.070–1.121)	13.83	0.031	63.8
Univariate	3	2.803 (1.652–7.856)	3.38	0.001	40.8

For peer review only

NMIBC, Non-muscle-invasive bladder cancer; PFS, progression-free survival.

For peer review only

For peer review only - http://bmjopen.bmj.com/site/about/guidelines.xhtml

Galbraith plot

Using Galbraith plot (**Figure 3A**), it was found that the study by Santos[33] was the main reason for the heterogeneity of RFS. After the aforementioned study was excluded, the remaining RFS studies had no significant heterogeneity according to the new meta-analysis (I^2 = 0.0%, P = 0.667). However, the expression of ki67 still had no statistically significant association with RFS (HR: 1.161, 95% CI: 0.896–1.504) (**Figure S1**). Using the Galbraith plot (**Figure 3B**), it was found that the study by Santos,[33] Park,[14] and van Rhijn[29] were the main reason for the heterogeneity of PFS. After these studies were excluded, the remaining RFS studies had no significant heterogeneity according to the new meta-analysis (I^2 = 0.0%, P = 0.497). The expression of ki67 still had a statistically significant association with PFS (HR: 2.922, 95% CI: 2.002–4.266) (**Figure S2**).

Meta-regression analysis

The meta-regression analysis indicated that the factors influencing heterogeneity (publication time, research region, sample size, stage, cutoff value, age, and follow-up period) might not be the reason for RFS heterogeneity. Publication time was the reason for heterogeneity of PFS (P = 0.036), but other factors were not (**Table S2**).

Publication bias

The funnel plot (**Figure 4A**) was basically symmetrical for RFS. The results of Begg's test and Egger's test showed P (Begg's) = 0.761, P (Egger's) = 0.601. The funnel plot (**Figure 4B**) was also basically symmetrical for PFS. The results of Begg's test and Egger's test showed P (Begg's) = 0.917, P (Egger's) = 0.964.

Discussion

A total of 11 published studies with 1321 cases complying with the inclusion criteria

For peer review only - http://bmjopen.bmj.com/site/about/guidelines.xhtml

were enrolled in this meta-analysis. The results of the meta-analysis indicated that the expression of ki67 had no statistically significant association with RFS, but it was significantly associated with PFS. The overexpression of ki67 was the risk factor for PFS. It suggested that ki67 was the prognostic predictive marker in patients with NMIBC treated with BCG. Besides, the aforementioned conditions did not change after excluding the published studies, possibly leading to heterogeneity and reperforming of the meta-analysis. It further proved that the result of the aforementioned meta-analysis was stable, that is, the overexpression of ki67 was the risk factor for PFS. In the Caucasian subgroup for PFS, racial classification and regional factors might be crucial in the prognosis of patients with NMIBC after BCG therapy. This might be related to the existence of different drug gene susceptibilities in people belonging to different races and living areas. The two subgroups were based on age in PFS, suggesting that age might be the important factor influencing the prognosis of bladder cancer. This also complies with our clinical practice. The elder the patient, the worse the prognosis. Several factors led to heterogeneity in the aforementioned subgroup analysis: (1) Due to the influence of race and environment, the documents included in this study came from different regions and countries. A large number of studies confirmed the differences in disease susceptibility between ethnic groups and regions. (2) Differences existed in the operation of health care workers in TUR and BGC intravesical immunotherapy because of different regions and different clinicians, such as surgical clearance of the tumor. The tumor with a broad base surface is often not easy to remove completely, which also depends on the surgeon's experience and surgical skills. In addition, the quality of BCG manufacturers may vary from region to region. (3) Different literature might include the bias of research object, research design, measuring instrument, and so on.

For peer review only - http://bmjopen.bmj.com/site/about/guidelines.xhtml

However, in general, heterogeneity did not affect the conclusion. Besides, the meta-regression analysis indicated publication time as the reason for PFS heterogeneity. It might be related to the improvement in testing technology, research level, and the quality and number of published studies, facilitating follow-up studies. As all the original data extracted from published studies on the association between the expression of ki67 and RFS in patients with NMIBC treated with BCG were multivariate, the result was considered to be precise because the HRs were adjusted, excluding the confounding factors such as age and gender. However, the original data extracted from published analyses on the association between the expression of ki67 and PFS were both multivariate and univariate. It was believed that the aforementioned adjustments did not have a significant impact on meta-analyses. Besides, according to the funnel plot, Begg's test and Egger's test, the enrolled studies had no statistically significant publication bias. Thus, the reliability of the present meta-analysis was high.

In 2016, the European Association of Urology (EUA)[36] recommended a scoring system for the prognostic evaluation of NMIBC based on six clinical and pathological factors proposed by the European Organization for the Research and Treatment of Cancer-Genito-Urinary Cancer Group (EORTC), including number of tumors, tumor size, prior recurrence rate, T category, presence of concurrent carcinoma in situ, and tumor grade (**Table S3**). The tumors were categorized into low-risk tumors, intermediate-risk tumors, and high-risk tumors using this assessment system to evaluate the prognosis. For the patients after BCG therapy, the EUA recommended another risk calculator developed by the Club Urologico Espanol de Tratamiento Oncologico (CUETO) and the EORTC. This calculator was based on gender, age, recurrent tumor, number of tumors, T category, associated Tis, and grade. The

For peer review only - http://bmjopen.bmj.com/site/about/guidelines.xhtml

CUETO risk calculator can be achieved at http://www.aeu.es/Cueto.html. The recommended level was B grade for the two scales for patients with NMIBC, whether used alone or combined. The two scales could be used together in the clinic. When using the CUETO scale, the calculated recurrent risk was lower than that from the EORTC scale,[37] which might be related to the special design in the CUETO scale for the patients receiving BCG intravesical immunotherapy. However, the scoring system only depending on clinical and pathological factors could not accurately evaluate the prognosis of patients with bladder cancer in T1 stage due to the independence of disease condition in each patient.[38] The markers regulated at the genetic level may judge the prognosis of patients with bladder cancer with the development of precision medicine. A reliable marker helps in recognizing the patients who have failed in BCG intravesical immunotherapy with high risk in time. Hence, these patients can undergo radical cystectomy or other treatments in time. Unfortunately, no prognostic marker has been applied in clinic currently. The results of this study potentially help to remind clinicians that patients with high expression of ki67 may need to develop more personalized follow-up plans, such as shorter follow-up and cystoscopy cycles. Patients with high risk of clinical evaluation of the guidelines and overexpression of ki67 may need to promptly change the treatment strategy.

Ki67 is a nucleoprotein that can be detected in the cell cycles except G0 phase.[39] The expression of human ki67 protein is closely related to proliferation. Therefore, it is an ideal marker to confirm the growth fraction of specific cell colonies.[40] Ki67 is a widely known amplified biomarker. The ki67 monoclonal antibody can be detected by the immunohistochemical method.[41] Ki67 has been proved to be a good proliferation marker in different cancers, including bladder cancer.[42]

So far, some meta-analyses have studied the effect of ki67 on the prognostic quality of

For peer review only - http://bmjopen.bmj.com/site/about/guidelines.xhtml

life of patients with esophageal cancer, breast cancer, epithelial ovarian cancer, and so on.[43-45] Some studies have also focused on the other aspects of bladder cancer. Using meta-analysis, Luo[46] believed that a high reactivity of ki67 could predict the poor prognosis in patients with bladder cancer. The univariate analysis showed that cancer-specific survival, disease-free survival, overall survival , PFS, and RFS had a significant association with poor prognosis in patients with a high reactivity of ki67. However, this study enrolled all types of bladder tumors and all the therapies for NMIBC. Currently, the bladder cancer treated in the clinic is mainly NMIBC. Thus, most of the applied therapy is TUR combined with installations of chemotherapy or BCG intravesical immunotherapy based on the patients' conditions. Therefore, this analysis had a certain limitation in the prognosis of patients with NMIBC after BCG intravesical immunotherapy.

Currently, few evidence-based studies focused on the prognosis of patients with NMIBC after BCG intravesical immunotherapy. Using meta-analysis, Zhou[47] analyzed the association between the expression of p53 and quality of life of patients with NMIBC after BCG intravesical immunotherapy. They believed that the overexpression of p53 in patients with NMIBC treated with BCG might be associated with RFS, especially in Asian population. Similarly, Du[48] also performed the meta-analysis on the relationship between p53 status and NMIBC in T1 stage and believed that the overexpression of p53 might be related to the development of NMIBC. The present study indicated that the overexpression of ki67 was the risk factor for PFS, but the expression of ki67 had no statistically significant association with RFS. P53 is the most common inactivated tumor suppressor gene in tumor cells.[49] The inactivation of p53 may cause cell abnormal hyperplasia and

For peer review only - http://bmjopen.bmj.com/site/about/guidelines.xhtml

cancerization. The variation in p53 results in enhanced proliferation, invasion, and metabolism.[50] The increase in the expression of ki67 , as cell proliferation marker suggests enhanced proliferation.[40] As a tumor suppressor gene with complicated function, p53 has a wider range of effects. The accuracy in the prediction of quality of life may not be more appropriate compared with ki67. The genetic difference between Asians and Caucasians suggests that different prediction systems should be built for different races. Besides, p27, E2F1, ezrin, and CK20 were also studied in other investigations for predicting NMIBC prognosis, which could be explored further comparing the advantages of using them alone or combined.

However, this study still had some limitations. First, the enrolled published studies involved different populations, used similar detection equipment, and had different cutoff values. All these reasons might have led to heterogeneity. Further, the sample size of the meta-analysis also limited its significance. Second, the meta-analysis included English published studies. Although Begg's test and Egger's test did not suggest publication bias, this study was still influenced by some bias. Finally, the surgical skills were different in different published studies, affecting the judgment regarding the effectiveness of BCG.

Conclusions

The overexpression of ki67 was the risk factor for PFS in patients with NMIBC after TUR and BCG intravesical immunotherapy, but the relationship between the expression of ki67 and RFS was not statistically significant. Owing to the aforementioned limitations of the present study, RCTs with large sample size are still required to validate the results.

For peer review only - http://bmjopen.bmj.com/site/about/guidelines.xhtml

Authors' contributions

YHH, NW, and XFZ conceived and designed the experiments. XC and YSD extracted the data. YHH, NW, and XFZ analyzed the data. ZSD, JFW, and XC contributed reagents/materials/analysis tools. YHH and NW wrote the paper. XFZ critically revised the report.

Funding

This study was supported by grants from the International S&T Cooperation Program of China (ISTCP) (Grant No.2014DFE30010).

Disclaimer

The contents of the present study are solely the responsibility of the author. The funders had no role in study design, data collection and analysis, decision to publish, or preparation of the manuscript.

Competing interests

None declared.

Data sharing statement

Extra data can be accessed via the Dryad data repository at http://datadryad.org/ with the https://doi.org/10.5061/dryad.hf06q72

References

1. Ploeg M, Aben KK, Kiemeney LA. The present and future burden of urinary bladder cancer in the world. *World J Urol* 2009;**27**:289-93. doi:10.1007/s00345-009-0383-3.

2. Torre LA, Bray F, Siegel RL, *et al.* Global cancer statistics, 2012. *CA Cancer J Clin* 2015;**65**:87-108. doi:10.3322/caac.21262.

3. Ro JY, Staerkel GA, Ayala AG. Cytologic and histologic features of superficial

For peer review only - http://bmjopen.bmj.com/site/about/guidelines.xhtml

bladder cancer. *Urol Clin North Am* 1992;**19**:435-53.

4. Woldu SL, Bagrodia A, Lotan Y. Guideline of guidelines: non-muscle-invasive bladder cancer. *BJU Int* 2017;**119**:371-80. doi:10.1111/bju.13760.

5. Brausi M, Collette L, Kurth K, *et al.* Variability in the recurrence rate at first follow-up cystoscopy after TUR in stage Ta T1 transitional cell carcinoma of the bladder: a combined analysis of seven EORTC studies. *Eur Urol* 2002;**41**:523-31.

6. Jarvinen R, Kaasinen E, Sankila A, *et al.* Long-term efficacy of maintenance bacillus Calmette-Guerin versus maintenance mitomycin C instillation therapy in frequently recurrent TaT1 tumours without carcinoma in situ: a subgroup analysis of the prospective, randomised FinnBladder I study with a 20-year follow-up. *Eur Urol* 2009;**56**:260-5. doi:10.1016/j.eururo.2009.04.009.

7. Sylvester RJ, Brausi MA, Kirkels WJ, *et al.* Long-term efficacy results of EORTC genito-urinary group randomized phase 3 study 30911 comparing intravesical instillations of epirubicin, bacillus Calmette-Guerin, and bacillus Calmette-Guerin plus isoniazid in patients with intermediate- and high-risk stage Ta T1 urothelial carcinoma of the bladder. *Eur Urol* 2010;**57**:766-73. doi:10.1016/j.eururo.2009.12.024.

8. Gual Frau J, Palou J, Rodriguez O*, et al.* Failure of Bacillus Calmette-Guerin therapy in non-muscle-invasive bladder cancer: Definition and treatment options. *Arch Esp Urol* 2016;**69**:423-33.

9. Boyd LA. Intravesical Bacillus Calmette-Guerin for treating bladder cancer. *Urol Nurs* 2003;**23**:189-91, 99; quiz 92.

10. Alkhateeb SS, Neill M, Bar-Moshe S, *et al.* Long-term prognostic value of the combination of EORTC risk group calculator and molecular markers in non-muscle-invasive bladder cancer patients treated with intravesical Bacille

For peer review only - http://bmjopen.bmj.com/site/about/guidelines.xhtml

Calmette-Guerin. *Urol Ann* 2011;**3**:119-26. doi:10.4103/0974-7796.84954.

11. Mariappan P, Smith G. A surveillance schedule for G1Ta bladder cancer allowing efficient use of check cystoscopy and safe discharge at 5 years based on a 25-year prospective database. *J Urol* 2005;**173**:1108-11. doi:10.1097/01.ju.0000149163.08521.69.

12. Bertz S, Otto W, Denzinger S, *et al.* Combination of CK20 and Ki-67 immunostaining analysis predicts recurrence, progression, and cancer-specific survival in pT1 urothelial bladder cancer. *Eur Urol* 2014;**65**:218-26. doi:10.1016/j.eururo.2012.05.033.

13. Kim K, Cho YM, Park BH, *et al.* Histological and immunohistochemical markers for progression prediction in transurethrally resected high-grade non-muscle invasive bladder cancer. *Int J Clin Exp Pathol* 2015;**8**:743-50.

14. Park J, Song C, Shin E, *et al.* Do molecular biomarkers have prognostic value in primary T1G3 bladder cancer treated with bacillus Calmette-Guerin intravesical therapy? *Urol Oncol* 2013;**31**:849-56. doi:10.1016/j.urolonc.2011.06.004.

15. Kruger S, Thorns C, Stocker W, *et al.* Prognostic value of MCM2 immunoreactivity in stage T1 transitional cell carcinoma of the bladder. *Eur Urol* 2003;**43**:138-45.

16. Oderda M, Ricceri F, Pisano F, *et al.* Prognostic factors including Ki-67 and p53 in Bacillus Calmette-Guerin-treated non-muscle-invasive bladder cancer: a prospective study. *Urol Int* 2013;**90**:184-90. doi:10.1159/000343431.

17. Zlotta AR, Noel JC, Fayt I, *et al.* Correlation and prognostic significance of p53, p21WAF1/CIP1 and Ki-67 expression in patients with superficial bladder tumors treated with bacillus Calmette-Guerin intravesical therapy. *J Urol* 1999;**161**:792-8.

18. Saint F, Salomon L, Quintela R, *et al.* Do prognostic parameters of remission

For peer review only

versus relapse after Bacillus Calmette-Guerin (BCG) immunotherapy exist?. analysis of a quarter century of literature. *Eur Urol* 2003;**43**:351-60; discussion 60-1.

19. Habuchi T, Marberger M, Droller MJ, *et al.* Prognostic markers for bladder cancer: International Consensus Panel on bladder tumor markers. *Urology* 2005;**66**:64-74. doi:10.1016/j.urology.2005.08.065.

20. Button KS, Ioannidis JP, Mokrysz C, *et al.* Power failure: why small sample size undermines the reliability of neuroscience. *Nat Rev Neurosci* 2013;**14**:365-76. doi:10.1038/nrn3475.

21. Liberati A, Altman DG, Tetzlaff J, *et al.* The PRISMA statement for reporting systematic reviews and meta-analyses of studies that evaluate health care interventions: explanation and elaboration. *PLoS Med* 2009;**6**:e1000100. doi:10.1371/journal.pmed.1000100.

22. Stang A. Critical evaluation of the Newcastle-Ottawa scale for the assessment of the quality of nonrandomized studies in meta-analyses. *Eur J Epidemiol* 2010;**25**:603-5. doi:10.1007/s10654-010-9491-z.

23. Parmar MK, Torri V, Stewart L. Extracting summary statistics to perform meta-analyses of the published literature for survival endpoints. *Stat Med* 1998;**17**:2815-34.

24. DerSimonian R, Laird N. Meta-analysis in clinical trials. *Control Clin Trials* 1986;**7**:177-88.

25. Higgins JP, Thompson SG. Quantifying heterogeneity in a meta-analysis. *Stat Med* 2002;**21**:1539-58. doi:10.1002/sim.1186.

26. Bax L, Ikeda N, Fukui N, *et al.* More than numbers: the power of graphs in meta-analysis. *Am J Epidemiol* 2009;169:249-55. doi:10.1093/aje/kwn340.

27. Xu X, Cheng Y, Li S, *et al.* Dietary carrot consumption and the risk of prostate

For peer review only

For peer review only - http://bmjopen.bmj.com/site/about/guidelines.xhtml

cancer. *Eur J Nutr* 2014;53:1615-1623. doi: 10.1007/s00394-014-0667-2.

28. Quintero A, Alvarez-Kindelan J, Luque RJ, *et al.* Ki-67 MIB1 labelling index and the prognosis of primary TaT1 urothelial cell carcinoma of the bladder. *J Clin Pathol* 2006;**59**:83-8. doi:10.1136/jcp.2004.022939.

29. van Rhijn BW, Liu L, Vis AN, *et al.* Prognostic value of molecular markers, sub-stage and European Organisation for the Research and Treatment of Cancer risk scores in primary T1 bladder cancer. *BJU Int* 2012;**110**:1169-76. doi:10.1111/j.1464-410X.2012.10996.x.

30. Burger M, Denzinger S, Hartmann A, *et al.* Mcm2 predicts recurrence hazard in stage Ta/T1 bladder cancer more accurately than CK20, Ki67 and histological grade. *Br J Cancer* 2007;**96**:1711-5. doi:10.1038/sj.bjc.6603784.

31. Queipo-Zaragoza JA, Ruiz-Cerda JL, Vera-Donoso CD, *et al.* Prognostic value of p53, Ki-67, microstaging and microvessel density in pT1G3 bladder tumors: creation of risk groups for progression. *Scand J Urol Nephrol* 2007;**41**:283-9. doi:10.1080/00365590601183055.

32. Lopez-Beltran A, Luque RJ, Alvarez-Kindelan J, *et al.* Prognostic factors in stage T1 grade 3 bladder cancer survival: the role of G1-S modulators (p53, p21Waf1, p27kip1, Cyclin D1, and Cyclin D3) and proliferation index (ki67-MIB1). *Eur Urol* 2004;**45**:606-12. doi:10.1016/j.eururo.2003.11.011.

33. Santos L, Amaro T, Costa C, *et al.* Ki-67 index enhances the prognostic accuracy of the urothelial superficial bladder carcinoma risk group classification. *Int J Cancer* 2003;**105**:267-72. doi:10.1002/ijc.11049.

34. Blanchet P, Droupy S, Eschwege P, *et al.* Prospective evaluation of Ki-67 labeling in predicting the recurrence and progression of superficial bladder transitional cell carcinoma. *Eur Urol* 2001;**40**:169-75. doi:49768.

For peer review only - http://bmjopen.bmj.com/site/about/guidelines.xhtml

35. Lee E, Park I, Lee C. Prognostic markers of intravesical bacillus Calmette-Guerin therapy for multiple, high-grade, stage T1 bladder cancers. *Int J Urol* 1997;**4**:552-6.

36. Babjuk M, Bohle A, Burger M*, et al.* EAU Guidelines on Non-Muscle-invasive Urothelial Carcinoma of the Bladder: Update 2016. *Eur Urol* 2017;**71**:447-61. doi:10.1016/j.eururo.2016.05.041.

37. Fernandez-Gomez J, Madero R, Solsona E, *et al.* Predicting nonmuscle invasive bladder cancer recurrence and progression in patients treated with bacillus Calmette-Guerin: the CUETO scoring model. *J Urol* 2009;**182**:2195-203. doi:10.1016/j.juro.2009.07.016.

38. Kiemeney LA, Witjes JA, Heijbroek RP, *et al.* Predictability of recurrent and progressive disease in individual patients with primary superficial bladder cancer. *J Urol* 1993;**150**:60-4.

39. Gerdes J, Lemke H, Baisch H, *et al.* Cell cycle analysis of a cell proliferation-associated human nuclear antigen defined by the monoclonal antibody Ki-67. *J Immunol* 1984;**133**:1710-5.

40. Scholzen T, Gerdes J. The Ki-67 protein: from the known and the unknown. *J Cell Physiol* 2000;**182**:311-22. doi:10.1002/(sici)1097-4652(200003)182:3<311::aid-jcp1>3.0.co;2-9.

41. Gerdes J, Li L, Schlueter C, *et al.* Immunobiochemical and molecular biologic characterization of the cell proliferation-associated nuclear antigen that is defined by monoclonal antibody Ki-67. *Am J Pathol* 1991;**138**:867-73.

42. Rodriguez-Alonso A, Pita-Fernandez S, Gonzalez-Carrero J, *et al.* p53 and ki67 expression as prognostic factors for cancer-related survival in stage T1 transitional cell bladder carcinoma. *Eur Urol* 2002;**41**:182-8; discussion 88-9.

43. McCormick Matthews LH, Noble F, Tod J, *et al.* Systematic review and

For peer review only

For peer review only - http://bmjopen.bmj.com/site/about/guidelines.xhtml

meta-analysis of immunohistochemical prognostic biomarkers in resected oesophageal adenocarcinoma. *Br J Cancer* 2015;**113**:107-18. doi:10.1038/bjc.2015.179.

44. Gui Y, Xu S, Yang X, *et al.* A meta-analysis of biomarkers for the prognosis of triple-negative breast cancer patients. *Biomark Med* 2016;**10**:771-90. doi:10.2217/bmm-2015-0064.

45. Qiu HL, Deng SZ, Li C, *et al.* High expression of KIF14 is associated with poor prognosis in patients with epithelial ovarian cancer. *Eur Rev Med Pharmacol Sci* 2017;**21**:239-45.

46. Luo Y, Zhang X, Mo M, *et al.* High Ki-67 Immunohistochemical Reactivity Correlates With Poor Prognosis in Bladder Carcinoma: A Comprehensive Meta-Analysis with 13,053 Patients Involved. *Medicine (Baltimore)* 2016;**95**:e3337. doi:10.1097/md.0000000000003337.

47. Zhou X, Zhang G, Tian Y. p53 Status correlates with the risk of recurrence in non-muscle invasive bladder cancers treated with Bacillus Calmette-Guerin: a meta-analysis. *PLoS One* 2015;**10**:e0119476. doi:10.1371/journal.pone.0119476.

48. Du J, Wang SH, Yang Q, *et al.* p53 status correlates with the risk of progression in stage T1 bladder cancer: a meta-analysis. *World J Surg Oncol* 2016;**14**:137. doi:10.1186/s12957-016-0890-9.

49. Fridman JS, Lowe SW. Control of apoptosis by p53. *Oncogene* 2003;**22**:9030-40. doi:10.1038/sj.onc.1207116.

50. Muller PA, Vousden KH, Norman JC. p53 and its mutants in tumor cell migration and invasion. *J Cell Biol* 2011;**192**:209-18. doi:10.1083/jcb.201009059.

Figure File

For peer review only

For peer review only - http://bmjopen.bmj.com/site/about/guidelines.xhtml

Figure 1. Flow diagram of study selection.

Figure 2. Forest plots of HRs estimated for the relationship between the expression of ki67 and RFS (A) or PFS (B) among patients in NMIBC treated with BCG.

Figure 3. Galbraith plot analysis was used to evaluate heterogeneity and RFS (A) or PFS (B).

Figure 4. Funnel plots of the expression of ki67 and RFS (A) or PFS (B).

Table File

Table 1. Main characteristics of all studies included in this meta-analysis.

Table 2. Quality of the included studies assessed by NOS.

Table 3. Subgroup results of RFS and heterogeneity test.

Table 4. Subgroup results of PFS and heterogeneity test.

Supplementary File

File S1. Electronic search strategy in PubMed.

Table S1. PRISMA.

Table S2. Meta-regression analysis of RFS and PFS.

Table S3. Risk group stratification in NMIBC.

Figure S1. Forest plots of HRs estimated for the relationship between the expression of ki67 and RFS after the aforementioned study was excluded.

Figure S2. Forest plots of HRs estimated for the relationship between the expression of ki67 and PFS after the aforementioned study was excluded.

For peer review only

For peer review only - http://bmjopen.bmj.com/site/about/guidelines.xhtml

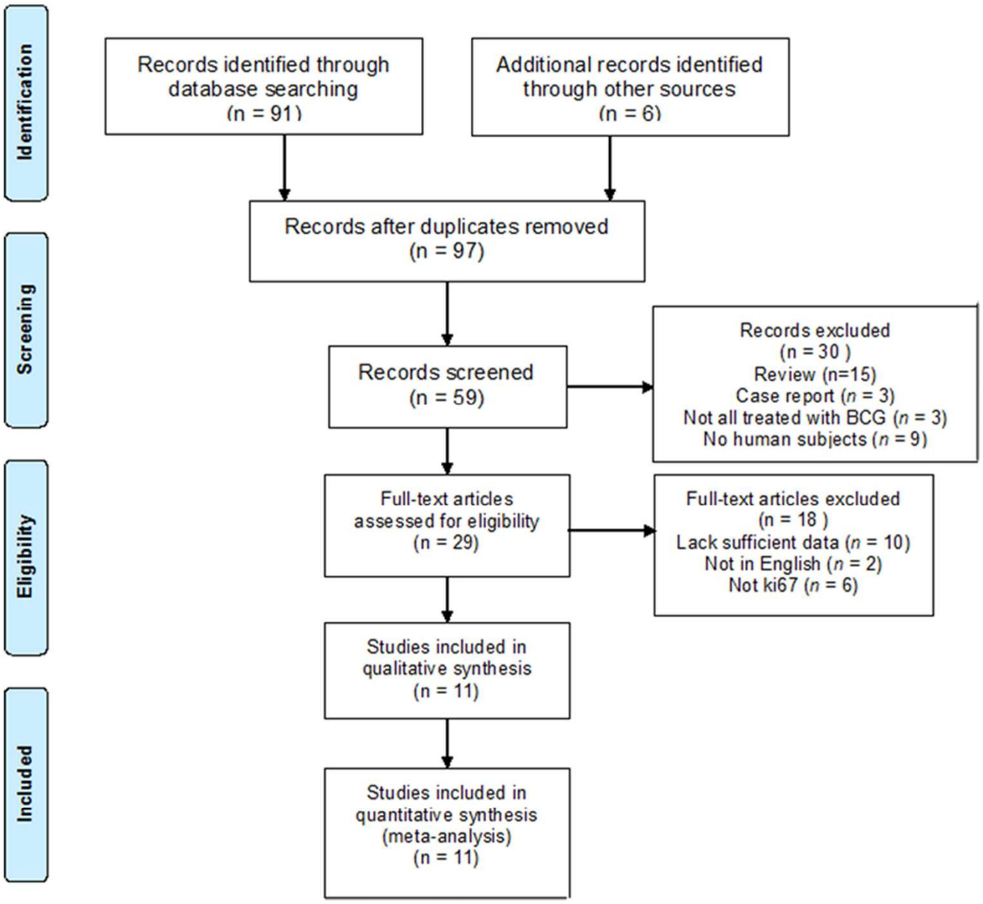

Figure 1. Flow diagram of study selection.

80x72mm (300 x 300 DPI)

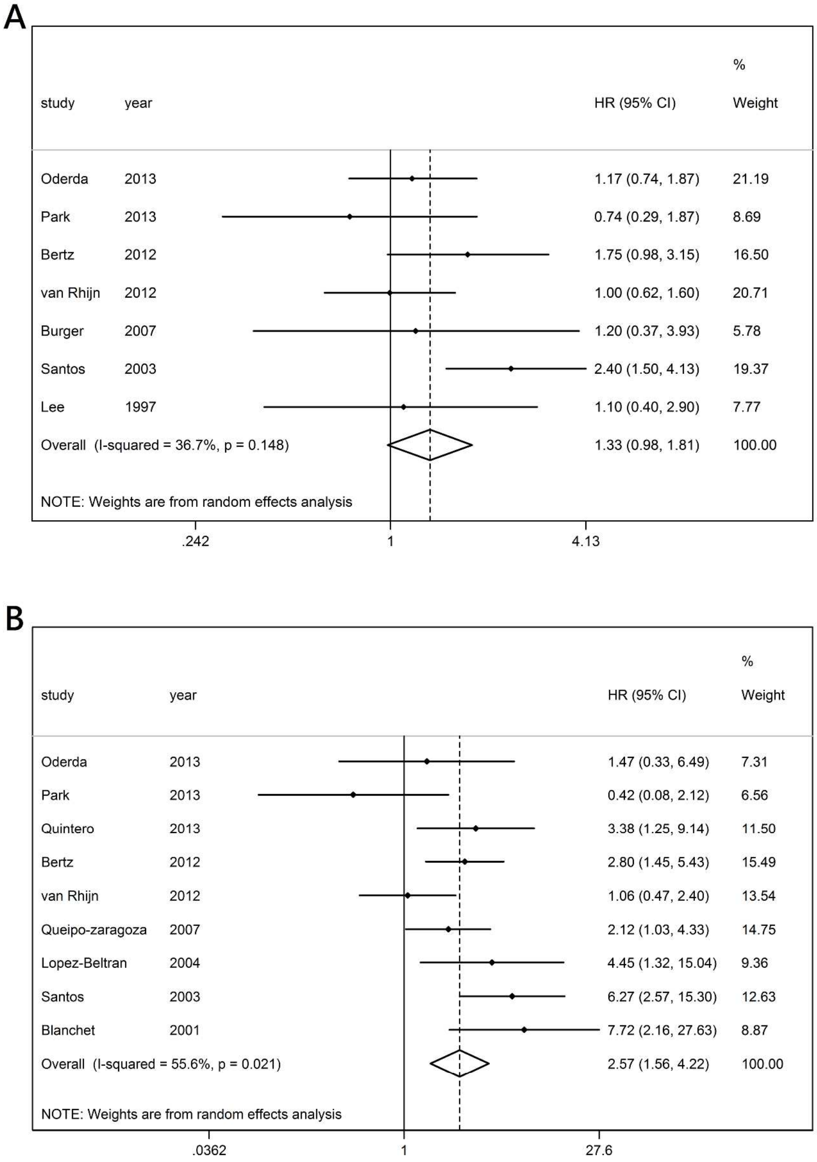

Forest plots of HRs estimated for the relationship between the expression of ki67 and RFS (A) or PFS (B) among patients in NMIBC treated with BCG.

267x381mm (300 x 300 DPI)

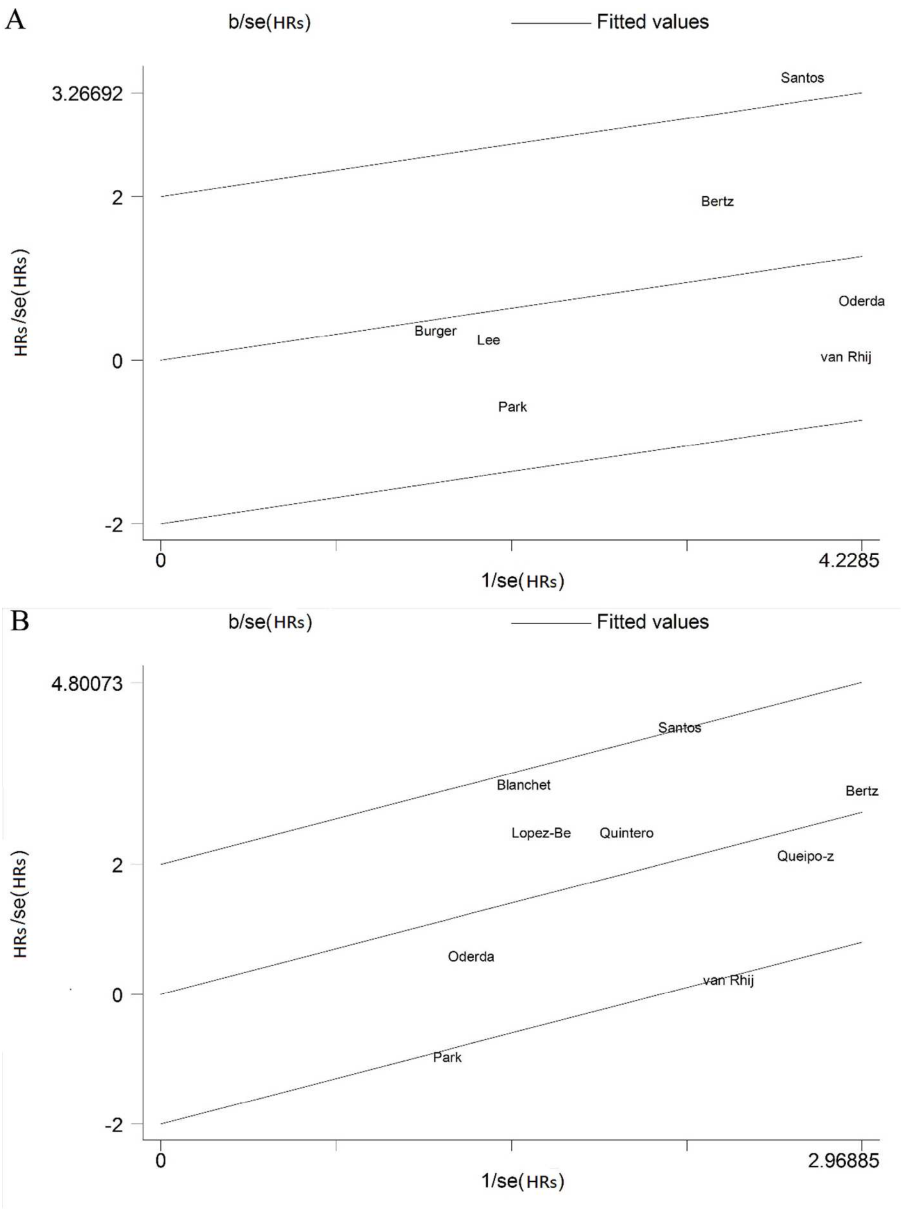

Galbraith plot analysis was used to evaluate heterogeneity and RFS (A) or PFS (B).

268x358mm (300 x 300 DPI)

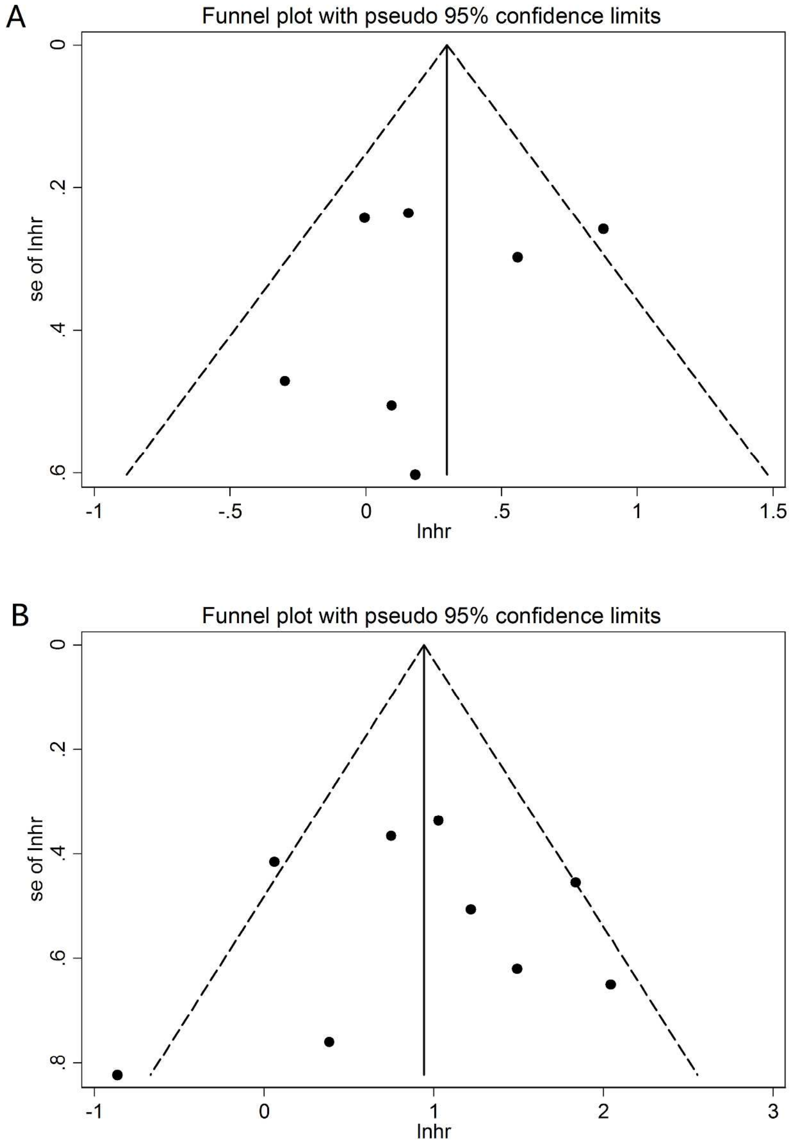

Funnel plots of the expression of ki67 and RFS (A) or PFS (B).

267x392mm (300 x 300 DPI)

Prognostic Value of ki67 in Bacillus Calmette–Guérin-Treated Non-muscle-Invasive Bladder Cancer: a Meta-analysis and Systematic Review

Search strategy in PubMed.

The last quest was updated on May 24, 2017.

#1 Search “Urinary Bladder Neoplasms” [Mesh]

#2 Search bladder cancer

#3 Search bladder carcinoma

#4 Search bladder tumor

#5 Search (#1 or #2 or #3 or #4)

#6 Search "BCG Vaccine" [Mesh]

#7 Search BCG

#8 Search Bacillus Calmette–Guérin

#9 Search (#6 or #7 or #8)

#10 Search "ki-67 antigen" [Mesh]

#11 Search ki-67

#12 Search ki67

#13 Search MBI-1

#14 Search (#10 or #11 or #12 or #13)

#15 Search (#5 and #9 and #14)

The enrolled contents included the reference lists and relevant suggestive references while searching.

For peer review only

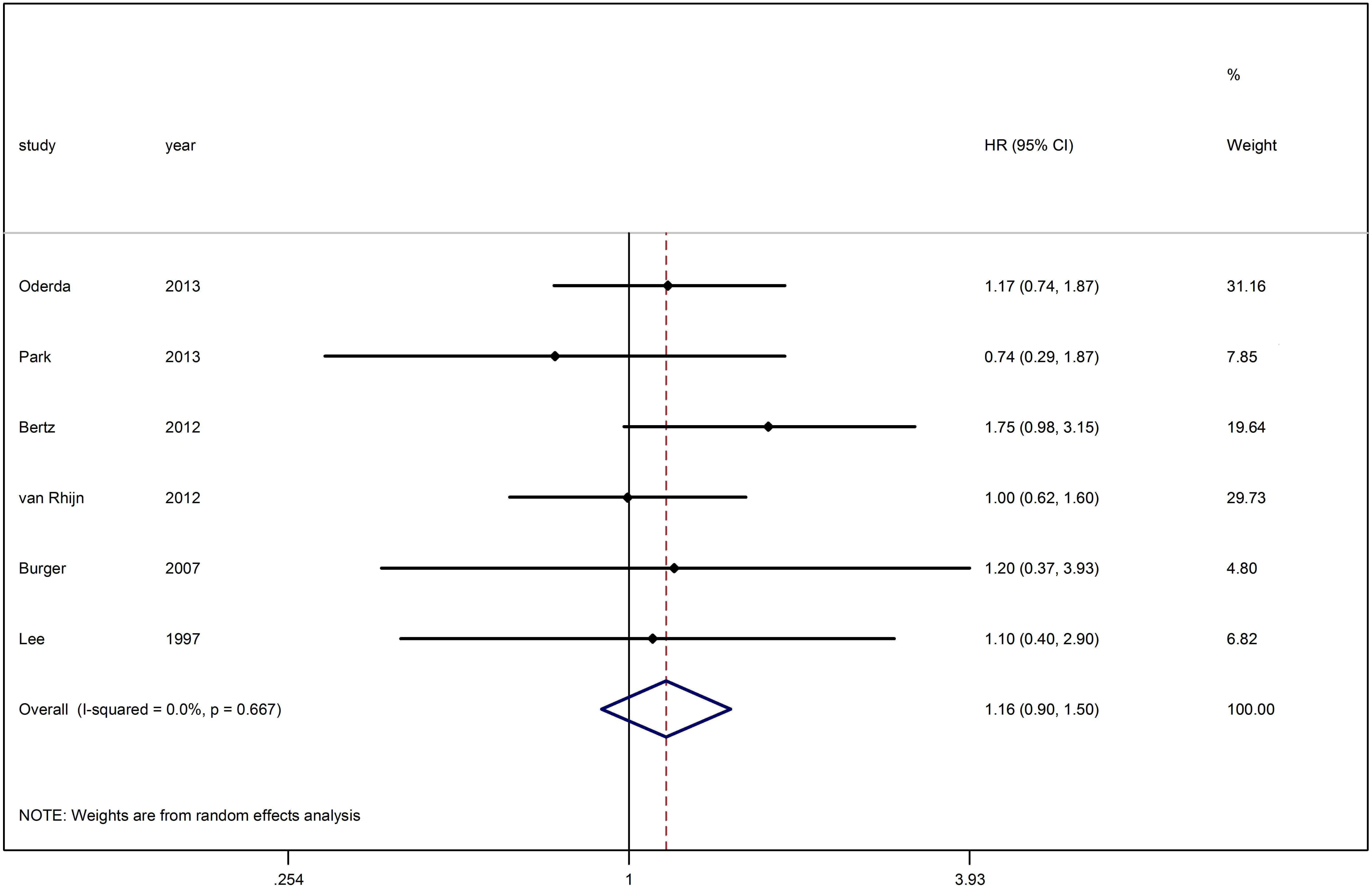

Figure S1. Forest plots of HRs estimated for the relationship between the expression of ki67 and RFS after the aforementioned study was excluded

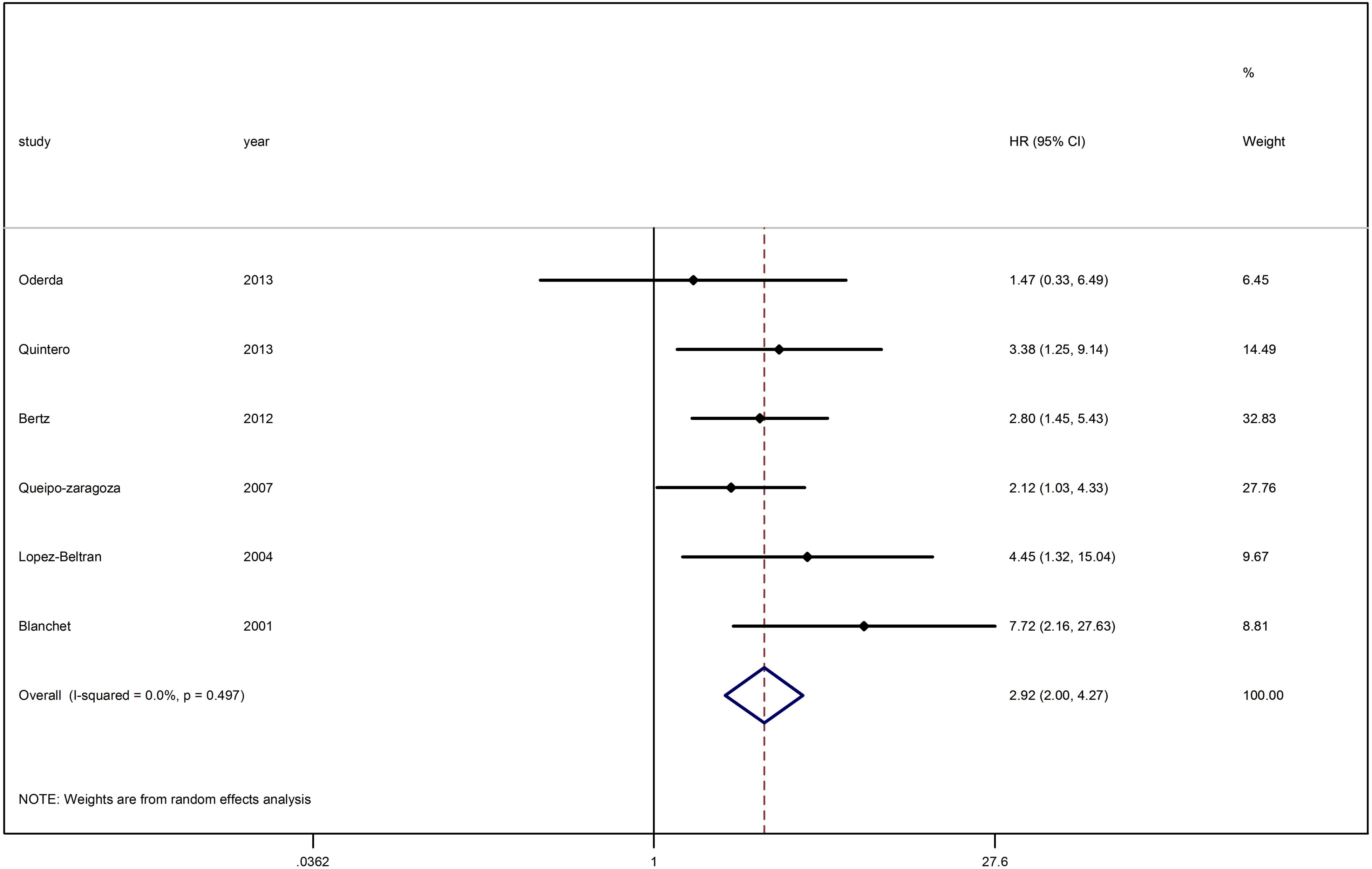

Figure S2. Forest plots of HRs estimated for the relationship between the expression of ki67 and PFS after the aforementioned study was excluded

PRISMA 2009 Checklist

Section/topic	#	Checklist item	Reported on page #
**TITLE**			
Title	1	Identify the report as a systematic review, meta-analysis, or both.	1
**ABSTRACT**			
Structured summary	2	Provide a structured summary including, as applicable: background; objectives; data sources; study eligibility criteria, participants, and interventions; study appraisal and synthesis methods; results; limitations; conclusions and implications of key findings; systematic review registration number.	3
**INTRODUCTION**			
Rationale	3	Describe the rationale for the review in the context of what is already known.	4-6
Objectives	4	Provide an explicit statement of questions being addressed with reference to participants, interventions, comparisons, outcomes, and study design (PICOS).	4-6
**METHODS**			
Protocol and registration	5	Indicate if a review protocol exists, if and where it can be accessed (e.g., Web address), and, if available, provide registration information including registration number.	7
Eligibility criteria	6	Specify study characteristics (e.g., PICOS, length of follow-up) and report characteristics (e.g., years considered, language, publication status) used as criteria for eligibility, giving rationale.	7
Information sources	7	Describe all information sources (e.g., databases with dates of coverage, contact with study authors to identify additional studies) in the search and date last searched.	7
Search	8	Present full electronic search strategy for at least one database, including any limits used, such that it could be repeated.	7
Study selection	9	State the process for selecting studies (i.e., screening, eligibility, included in systematic review, and, if applicable, included in the meta-analysis).	7-8
Data collection process	10	Describe method of data extraction from reports (e.g., piloted forms, independently, in duplicate) and any processes for obtaining and confirming data from investigators.	7-8
Data items	11	List and define all variables for which data were sought (e.g., PICOS, funding sources) and any assumptions and simplifications made.	8-9
Risk of bias in individual studies	12	Describe methods used for assessing risk of bias of individual studies (including specification of whether this was done at the study or outcome level), and how this information is to be used in any data synthesis.	9
Summary measures	13	State the principal summary measures (e.g., risk ratio, difference in means).	8-9
Synthesis of results	14	Describe the methods of handling data and combining results of studies, if done, including measures of consistency (e.g., I^2) for each meta-analysis.	8-9

For peer review only - http://bmjopen.bmj.com/site/about/guidelines.xhtml

PRISMA 2009 Checklist

Section/topic	#	Checklist item	Reported on page #
Risk of bias across studies	15	Specify any assessment of risk of bias that may affect the cumulative evidence (e.g., publication bias, selective reporting within studies).	9
Additional analyses	16	Describe methods of additional analyses (e.g., sensitivity or subgroup analyses, meta-regression), if done, indicating which were pre-specified.	9
**RESULTS**			
Study selection	17	Give numbers of studies screened, assessed for eligibility, and included in the review, with reasons for exclusions at each stage, ideally with a flow diagram.	9-10
Study characteristics	18	For each study, present characteristics for which data were extracted (e.g., study size, PICOS, follow-up period) and provide the citations.	10-12
Risk of bias within studies	19	Present data on risk of bias of each study and, if available, any outcome level assessment (see item 12).	17
Results of individual studies	20	For all outcomes considered (benefits or harms), present, for each study: (a) simple summary data for each intervention group (b) effect estimates and confidence intervals, ideally with a forest plot.	13-16
Synthesis of results	21	Present results of each meta-analysis done, including confidence intervals and measures of consistency.	9-17
Risk of bias across studies	22	Present results of any assessment of risk of bias across studies (see Item 15).	17
Additional analysis	23	Give results of additional analyses, if done (e.g., sensitivity or subgroup analyses, meta-regression [see Item 16]).	17
**DISCUSSION**			
Summary of evidence	24	Summarize the main findings including the strength of evidence for each main outcome; consider their relevance to key groups (e.g., healthcare providers, users, and policy makers).	17-22
Limitations	25	Discuss limitations at study and outcome level (e.g., risk of bias), and at review-level (e.g., incomplete retrieval of identified research, reporting bias).	22
Conclusions	26	Provide a general interpretation of the results in the context of other evidence, and implications for future research.	22
**FUNDING**			
Funding	27	Describe sources of funding for the systematic review and other support (e.g., supply of data); role of funders for the systematic review.	23

From: Moher D, Liberati A, Tetzlaff J, Altman DG, The PRISMA Group (2009). Preferred Reporting Items for Systematic Reviews and Meta-Analyses: The PRISMA Statement. PLoS Med 6(6): e1000097. doi:10.1371/journal.pmed1000097

For more information, visit: www.prisma-statement.org.

For peer review only

For peer review only - http://bmjopen.bmj.com/site/about/guidelines.xhtml

Table S2. Meta-regression analysis of RFS and PFS

PFS

Heterogeneity factor	Coefficient	SE	*t*	*P*
Years	–0.0343	0.0283	–1.21	0.280
Country				
1	–0.3899	0.3716	–1.05	0.404
2	–0.7185	0.3503	–2.05	0.177
3	–0.9900	0.4307	–2.30	0.148
4	–0.8805	0.3541	–2.49	0.131
Numbers of patients	0.0022	0.0020	1.16	0.299
Stage	None			
Cutoff	–0.0098	0.0407	–0.24	0.819
Age	0.0021	0.0395	0.05	0.959
Follow-up	–0.0062	0.0065	–0.96	0.379

PFS

Heterogeneity factor	Coefficient	SE	*t*	*P*
Years	–0.1195	0.0461	–2.59	0.036
Country				

For peer review only

1	0.2080	0.7936	0.26	0.818
2	–0.8062	0.5662	–1.42	0.290
3	–1.4505	0.8858	–1.64	0.243
4	–2.7009	0.9407	–2.87	0.103
5	–1.7766	0.6167	–2.88	0.102
6	–0.8158	0.5281	–1.54	0.262
Numbers of patients	0.0006	0.0036	0.16	0.877
Stage				
1	–1.4505	1.5909	–0.91	0.458
2	–0.8062	1.4108	–0.57	0.625
3	0.2080	1.5332	0.14	0.904
4	–1.7766	1.4353	–1.24	0.341
5	–1.2560	1.2069	–1.04	0.407
6	–0.6170	1.4689	–0.42	0.715
Cutoff	–0.0177	0.0309	–0.57	0.585
Age	–0.0672	0.0757	–0.89	0.404
Follow-up	–0.0118	0.0159	–0.74	0.483

For peer review only

Table S3. Risk group stratification in NMIBC

**Low-risk tumors**	Primary, solitary, Ta, LG/G1, <3 cm, no CIS
**Intermediate-risk tumors**	All tumors not defined in the two adjacent categories (between the category of low and high risk)
**High-risk tumors**	Any of the following:<br>T1 tumor<br>HG/G3 tumor<br>CIS<br>Multiple, recurrent, and large (>3 cm) Ta G1G2 tumors (all conditions must be present at this point)

NMIBC, non-muscle-invasive bladder cancer; CIS, carcinoma in situ; HG, high grade; LG, low grade.

For peer review only

PRISMA 2009 Checklist

Section/topic	#	Checklist item	Reported on page #
**TITLE**			
Title	1	Identify the report as a systematic review, meta-analysis, or both.	1
**ABSTRACT**			
Structured summary	2	Provide a structured summary including, as applicable: background; objectives; data sources; study eligibility criteria, participants, and interventions; study appraisal and synthesis methods; results; limitations; conclusions and implications of key findings; systematic review registration number.	3
**INTRODUCTION**			
Rationale	3	Describe the rationale for the review in the context of what is already known.	4-6
Objectives	4	Provide an explicit statement of questions being addressed with reference to participants, interventions, comparisons, outcomes, and study design (PICOS).	4-6
**METHODS**			
Protocol and registration	5	Indicate if a review protocol exists, if and where it can be accessed (e.g., Web address), and, if available, provide registration information including registration number.	7
Eligibility criteria	6	Specify study characteristics (e.g., PICOS, length of follow-up) and report characteristics (e.g., years considered, language, publication status) used as criteria for eligibility, giving rationale.	7
Information sources	7	Describe all information sources (e.g., databases with dates of coverage, contact with study authors to identify additional studies) in the search and date last searched.	7
Search	8	Present full electronic search strategy for at least one database, including any limits used, such that it could be repeated.	7
Study selection	9	State the process for selecting studies (i.e., screening, eligibility, included in systematic review, and, if applicable, included in the meta-analysis).	7-8
Data collection process	10	Describe method of data extraction from reports (e.g., piloted forms, independently, in duplicate) and any processes for obtaining and confirming data from investigators.	7-8
Data items	11	List and define all variables for which data were sought (e.g., PICOS, funding sources) and any assumptions and simplifications made.	8-9
Risk of bias in individual studies	12	Describe methods used for assessing risk of bias of individual studies (including specification of whether this was done at the study or outcome level), and how this information is to be used in any data synthesis.	9
Summary measures	13	State the principal summary measures (e.g., risk ratio, difference in means).	8-9
Synthesis of results	14	Describe the methods of handling data and combining results of studies, if done, including measures of consistency (e.g., I^2) for each meta-analysis.	8-9

For peer review only - http://bmjopen.bmj.com/site/about/guidelines.xhtml

PRISMA 2009 Checklist

Section/topic	#	Checklist item	Reported on page #
Risk of bias across studies	15	Specify any assessment of risk of bias that may affect the cumulative evidence (e.g., publication bias, selective reporting within studies).	9
Additional analyses	16	Describe methods of additional analyses (e.g., sensitivity or subgroup analyses, meta-regression), if done, indicating which were pre-specified.	9
**RESULTS**			
Study selection	17	Give numbers of studies screened, assessed for eligibility, and included in the review, with reasons for exclusions at each stage, ideally with a flow diagram.	9-10
Study characteristics	18	For each study, present characteristics for which data were extracted (e.g., study size, PICOS, follow-up period) and provide the citations.	10-12
Risk of bias within studies	19	Present data on risk of bias of each study and, if available, any outcome level assessment (see item 12).	17
Results of individual studies	20	For all outcomes considered (benefits or harms), present, for each study: (a) simple summary data for each intervention group (b) effect estimates and confidence intervals, ideally with a forest plot.	13-16
Synthesis of results	21	Present results of each meta-analysis done, including confidence intervals and measures of consistency.	9-17
Risk of bias across studies	22	Present results of any assessment of risk of bias across studies (see Item 15).	17
Additional analysis	23	Give results of additional analyses, if done (e.g., sensitivity or subgroup analyses, meta-regression [see Item 16]).	17
**DISCUSSION**			
Summary of evidence	24	Summarize the main findings including the strength of evidence for each main outcome; consider their relevance to key groups (e.g., healthcare providers, users, and policy makers).	17-22
Limitations	25	Discuss limitations at study and outcome level (e.g., risk of bias), and at review-level (e.g., incomplete retrieval of identified research, reporting bias).	22
Conclusions	26	Provide a general interpretation of the results in the context of other evidence, and implications for future research.	22
**FUNDING**			
Funding	27	Describe sources of funding for the systematic review and other support (e.g., supply of data); role of funders for the systematic review.	23

From: Moher D, Liberati A, Tetzlaff J, Altman DG, The PRISMA Group (2009). Preferred Reporting Items for Systematic Reviews and Meta-Analyses: The PRISMA Statement. PLoS Med 6(6): e1000097. doi:10.1371/journal.pmed1000097

For more information, visit: **www.prisma-statement.org**.

For peer review only - http://bmjopen.bmj.com/site/about/guidelines.xhtml
